# Burden of 292 causes of death and life expectancy decomposition in Iran, 1990–2023: a systematic analysis for the Global Burden of Disease Study 2023

**DOI:** 10.1016/S2214-109X(26)00031-8

**Published:** 2026-04-23

**Authors:** GBD 2023 Iran Collaborators, Sadaf G Sepanlou, Hossein Rezaei Aliabadi, Madineh Abbasi, Hedayat Abbastabar, Arash Abdollahi, Hassan Abolhassani, Meysam Abolmaali, Dariush Abtahi, Seyed Mohammad Kazem Aghamir, Ali Ahmadi, Marjan Ajami, Mehran Alijanzadeh, Masoud Aman Mohammadi, Sohrab Amiri, Mohammad Hosein Amirzade-Iranaq, Saeid Anvari, Jalal Arabloo, Saeed Asgary, Seyyed Shamsadin Athari, Haleh Ayatollahi, Najmeh Bahmanziari, Mohammad-Mahdi Bastan, Maryam Bemanalizadeh, Milad Bonakdar Hashemi, Omid Dadras, Nicole Davis Weaver, Azizallah Dehghan, Shirin Djalalinia, Saeid Doaei, Ebrahim Eini, Sharareh Eskandarieh, Rana Ezzeddini, Aliasghar Fakhri-Demeshghieh, Majid Fasihi Harandi, Alireza Feizkhah, Behzad Foroutan, Fataneh Ghadirian, Sadegh Ghafarian, Fatemeh Ghaffarifar, Abolfazl Ghahramani, Sulmaz Ghahramani, Mahsa Ghajarzadeh, Amirhossein Ghaseminejad-Raeini, Ahmad Ghashghaee, Mahsa Ghorbani, Pouya Goleij, Mahdi Gouravani, Farrokh Habibzadeh, Zahra Hadian, Dariush Haghmorad, Nasrin Hanifi, Hamidreza Hasani, Ali Hasanpour- Dehkordi, Mahgol Sadat Hassan Zadeh Tabatabaei, Shokoufeh Hassani, Mohammad Heidari, Mojtaba Heydari, Mohammad-Salar Hosseini, Kiavash Hushmandi, Jalil Jaafari, Morteza Jafarinia, Kasra Jahankhani, Sepide Javankiani, Ali Kabir, Leila R Kalankesh, Samad Karkhah, Hengameh Kasraei, Sina Kazemian, Faham Khamesipour, Mohammad Khammarnia, Maryam Khayamzadeh, Sepehr Khosravi, Ali-Asghar Kolahi, Farzad Kompani, Razzagh Mahmoudi, Reza Malekzadeh, Vahid Mansouri, Hamid Reza Marateb, Hossein Masoumi-Asl, Elahe Meftah, Mojgan Mirghafourvand, Noushin Mohammadifard, Ibrahim Mohammadzadeh, Mohammad Mohseni, Ali H Mokdad, AmirAli Moodi Ghalibaf, Yousef Moradi, Mohammad Moradi-Joo, Amin Mousavi Khaneghah, Ayoub Nafei, Noureddin Nakhostin Ansari, Ali Nasrollahizadeh, Amir Nasrollahizadeh, Amir Nasrollahizadeh, Athare Nazri-Panjaki, Jalil Nejati, Ali Nikoobar, Hassan Okati-Aliabad, Reza Pourbabaki, Reza Rabiei, Hadi Raeisi Shahraki, Afarin Rahimi-Movaghar, Vafa Rahimi-Movaghar, Shayan Rahmani, Vahid Rahmanian, Ashkan Rasouli-Saravani, Ramin Ravangard, Nazila Rezaei, Negar Rezaei, Nima Rezaei, Peyman Rezaei Hachesu, Mohsen Rezaeian, Gholamreza Roshandel, Morteza Rostamian, Masoumeh Sadeghi, Maryam Saeedi, Sahar Saeedi Moghaddam, Mehdi Safari, Alireza Saghafi, Amirhossein Sahebkar, Leili Salehi, Amir Salek Farrokhi, Sohrab Salimi, Hossein Samadi Kafil, Mahan Shafie, Hamid R Shahsavari, Alireza Shakeri, Ali Shamekh, Mehran Shams-Beyranvand, Amin Sharifan, Amir Shiani, Reza Shirkoohi, Sina Shool, Seyed Afshin Shorofi, Soroush Soraneh, Seyed-Amir Tabatabaeizadeh, Ramin Tabibi, Alireza Tahamtan, Moslem Taheri Soodejani, Razieh Tavakoli Oliaee, Seyed Mohammad Tavangar, Omid Vakili, Sajad Yaghoubi, Habib Yaribeygi, Hamed Zandian, Alireza Zangeneh, Kourosh Zarea, Mohsen Naghavi

**Affiliations:** A Digestive Diseases Research Institute (DDRI), Tehran University of Medical Sciences, Tehran, Iran; B Department of Biostatistics, Shiraz University of Medical Sciences, Shiraz, Iran; C School of Medicine, Shiraz University of Medical Sciences, Shiraz, Iran; D Infectious and Tropical Research Center, Tabriz University of Medical Sciences, Tabriz, Iran; E Advanced Diagnostic and Interventional Radiology Research Center (ADIR), Tehran University of Medical Sciences, Tehran, Iran; F Minimally Invasive Surgery Research Center, Iran University of Medical Sciences, Tehran, Iran; G Research Center for Immunodeficiencies, Tehran University of Medical Sciences, Tehran, Iran; H Department of Medical Biochemistry and Biophysics, Karolinska Institute, Stockholm, Sweden; I Department of Neurosurgery, Iran University of Medical Sciences, Tehran, Iran; J Khatam Al-anbia Hospital, Shefa Neuroscience Research Center, Tehran, Iran; K Department of Anesthesiology, Shahid Beheshti University of Medical Sciences, Tehran, Iran; L Urology Research Center, Tehran University of Medical Sciences, Tehran, Iran; M Department of Epidemiology and Biostatistics, Shahrekord University of Medical Sciences, Shahrekord, Iran; N Department of Epidemiology, Shahid Beheshti University of Medical Sciences, Tehran, Iran; O National Nutrition and Food Technology Research Institute, Shahid Beheshti University of Medical Sciences, Tehran, Iran; P Social Determinants of Health Research Center, Qazvin University of Medical Sciences, Qazvin, Iran; Q Food and Beverages Safety Research Center, Urmia University of Medical Sciences, Urmia, Iran; R Spiritual Health Research Centre, Baqiyatallah University of Medical Sciences, Tehran, Iran; S Universal Scientific Education and Research Network (USERN), Tehran University of Medical Sciences, Tehran, Iran; T Regenerative Medicine, Organ Procurement and Transplantation Multi-disciplinary Center, Guilan University of Medical Sciences, Rasht, Iran; U Health Management and Economics Research Center, Iran University of Medical Sciences, Tehran, Iran; V Research Institute of Dental Sciences, Shahid Beheshti University of Medical Sciences, Tehran, Iran; W National Agency for Strategic Research in Medical Education (NASRME), Ministry of Health and Medical Education, Tehran, Iran; X Department of Immunology, Zanjan University of Medical Sciences, Zanjan, Iran; Y Department of Health Information Management, Iran University of Medical Sciences, Tehran, Iran; Z School of Public Health, Tehran University of Medical Sciences, Tehran, Iran; AA Non-communicable Diseases Research Center, Tehran University of Medical Sciences, Tehran, Iran; AB School of Medicine, Iran University of Medical Sciences, Tehran, Iran; AC Department of Pediatrics, Isfahan University of Medical Sciences, Isfahan, Iran; AD Department of Pediatric Neurology, Tehran University of Medical Sciences, Tehran, Iran; AE Department of Urology, Shahid Beheshti University of Medical Sciences, Tehran, Iran; AF Department of Health, Northern Territory Government, Darwin, SA, Australia; AG Institute for Health Metrics and Evaluation, University of Washington, Seattle, WA, USA; AH Department of Epidemiology and Community Medicine, Fasa University of Medical Sciences, Fasa, Iran; AI Development of Research and Technology Center, Ministry of Health and Medical Education, Tehran, Iran; AJ School of Health, Guilan University of Medical Sciences, Rasht, Iran; AK Department of Community Nutrition, Shahid Beheshti University of Medical Sciences, Tehran, Iran; AL Independent Consultant, Ahvaz, Iran; AM Multiple Sclerosis Research Center, Tehran University of Medical Sciences, Tehran, Iran; AN Clinical Biochemistry, Tarbiat Modares University, Tehran, Iran; AO Department of Food Hygiene and Quality Control, University of Tehran, Tehran, Iran; AP Department of Medical Parasitology, Kerman University of Medical Sciences, Kerman, Iran; AQ Department of Social Medicine and Epidemiology, Guilan University of Medical Sciences, Rasht, Iran; AR Department of Pharmacology, Iranshahr University of Medical Sciences, Iranshahr, Iran; AS School of Nursing and Midwifery, Shahid Beheshti University of Medical Sciences, Tehran, Iran; AT Department of Ophthalmology, Tehran University of Medical Sciences, Tehran, Iran; AU Department of Parasitology and Entomology, Tarbiat Modares University, Tehran, Iran; AV Department of Occupational Safety and Health, Urmia University of Medical Sciences, Urmia, Iran; AW Health Policy Research Center, Shiraz University of Medical Sciences, Shiraz, Iran; AX Department of Radiology, Washington University in St. Louis, St. Louis, MO, USA; AY Cardiovascular Epidemiology Research Center, Rajaie Cardiovascular Institute, Tehran, Iran; AZ School of Medicine, Tehran University of Medical Sciences, Tehran, Iran; BA School of Public Health, Qazvin University of Medical Sciences, Qazvin, Iran; BB Orthodontics Department, Mashhad University of Medical Sciences, Mashhad, Iran; BC Department of Genetics, Sana Institute of Higher Education, Sari, Iran; BD Universal Scientific Education and Research Network (USERN), Kermanshah University of Medical Sciences, Kermanshah, Iran; BE Global Virus Network, Middle East Region, Shiraz, Iran; BF Department of Immunology, Semnan University of Medical Sciences, Semnan, Iran; BG Cancer Research Center, Semnan University of Medical Sciences, Semnan, Iran; BH Department of Critical Care and Emergency Nursing, Zanjan University of Medical Sciences, Zanjan, Iran; BI Department of Ophthalmology, Alborz University of Medical Sciences, Karaj, Iran; BJ Department of Medical Surgical, Shahroud University of Medical Sciences, Shahrekord, Iran; BK Sina Trauma and Surgery Research Center, Tehran University of Medical Sciences, Tehran, Iran; BL The Institute of Pharmaceutical Sciences (TIPS), Tehran University of Medical Sciences, Tehran, Iran; BM Community-Oriented Nursing Midwifery Research Center, Shahrekord University of Medical Sciences, Shahrekord, Iran; BN Poostchi Ophthalmology Research Center, Shiraz University of Medical Sciences, Shiraz, Iran; BO Research Center for Evidence-Based Medicine, Tabriz University of Medical Sciences, Tabriz, Iran; BP Nephrology and Urology Research Center, Baqiyatallah University of Medical Sciences, Tehran, Iran; BQ Department of Environmental Health Engineering, Guilan University of Medical Sciences, Rasht, Iran; BR Shiraz Neuroscience Research Center, Shiraz University of Medical Sciences, Shiraz, Iran; BS Department of Immunology, Shahid Beheshti University of Medical Sciences, Tehran, Iran; BT Department of General Surgery, Tehran University of Medical Sciences, Tehran, Iran; BU School of Management and Medical Informatics, Tabriz University of Medical Sciences, Tabriz, Iran; BV Department of Medical-Surgical Nursing, Guilan University of Medical Sciences, Rasht, Iran; BW Eye Research Center, Iran University of Medical Sciences, Tehran, Iran; BX Cardiac Primary Prevention Research Center, Tehran University of Medical Sciences, Tehran, Iran; BY Department of Cardiac Electrophysiology, Tehran University of Medical Sciences, Tehran, Iran; BZ Food and Drug Research Center, Iran Food and Drug Administration, Tehran, Iran; CA Health Promotion Research Center, Zahedan University of Medical Sciences, Zahedan, Iran; CB Shahid Beheshti University of Medical Sciences, Tehran, Iran; CC Academy of Medical Science, Tehran, Iran; CD Manchester Centre for Clinical Neurosciences, Northern Care Alliance NHS Foundation Trust, Salford, United Kingdom; CE Department of Clinical Research, Icahn School of Medicine at Mount Sinai, New York City, NY, USA; CF Social Determinants of Health Research Center, Shahid Beheshti University of Medical Sciences, Tehran, Iran; CG Children's Medical Center, Tehran University of Medical Sciences, Tehran, Iran; CH Department of Food Hygiene and Safety, Qazvin University of Medical Sciences, Qazvin, Iran; CI Non-communicable Disease Research Center, Shiraz University of Medical Sciences, Shiraz, Iran; CJ Department of Biomedical Engineering, University of Isfahan, Isfahan, Iran; CK Institute for Research and Innovation in Health (IRIS), Universitat Politècnica de Catalunya (Barcelona Tech - UPC) (Polytechnic University of Catalonia), Barcelona, Spain; CL Department of Pediatrics, Iran University of Medical Sciences, Tehran, Iran; CM Clinical Research Development Center, Shiraz University of Medical Sciences, Shiraz, Iran; CN Professor Alborzi Clinical Microbiology Research Center, Shiraz University of Medical Sciences, Shiraz, Iran; CO Faculty of Nursing and Midwifery, Tabriz University of Medical Sciences, Tabriz, Iran; CP Isfahan Cardiovascular Research Center, Isfahan University of Medical Sciences, Isfahan, Iran; CQ Skull Base Research Center, Shahid Beheshti University of Medical Sciences, Tehran, Iran; CR Department of Health Services Management, Iran University of Medical Sciences, Iran, Iran; CS Department of Health Services Management, Isfahan University of Medical Sciences, Isfahan, Iran; CT Department of Health Metrics Sciences, School of Medicine, University of Washington, Seattle, WA, USA; CU Mashhad University of Medical Sciences, Mashhad, Iran; CV Department of Epidemiology and Biostatistics, Kurdistan University of Medical Sciences, Sanandaj, Iran; CW Social Determinants of Health Research Center, Yasuj University of Medical Sciences, Yasuj, Iran; CX Faculty of Biotechnologies (BioTech), ITMO University, Saint Petersburg, Russia; CY Elderly Health Research Center, Research and Academic Institution, Tehran, Iran; CZ Department of Physiotherapy, Tehran University of Medical Sciences, Tehran, Iran; DA Research Center for War-affected People, Tehran University of Medical Sciences, Tehran, Iran; DB Faculty of Medicine, Isfahan University of Medical Sciences, Isfahan, Iran; DC Tehran Heart Center, Tehran University of Medical Sciences, Tehran, Iran; DD Institute for Health Metrics and Evaluation, Tehran University of Medical Sciences, Tehran, Iran; DE Department of Health Promotion, Zahedan University of Medical Sciences, Zahedan, Iran; DF Department of Occupational Health and Safety Engineering, Kerman University of Medical Sciences, Kerman, Iran; DG Department of Health Information Technology and Management, Shahid Beheshti University of Medical Sciences, Tehran, Iran; DH Iranian National Center for Addiction Studies, Tehran University of Medical Sciences, Tehran, Iran; DI School of Medicine, Shahid Beheshti University of Medical Sciences, Tehran, Iran; DJ Department of Public Health, Torbat Jam Faculty of Medical Sciences, Torbat Jam, Iran; DK Department of Health Services Management, Shiraz University of Medical Sciences, Shiraz, Iran; DL Endocrinology and Metabolism Research Institute, Tehran University of Medical Sciences, Tehran, Iran; DM Network of Immunity in Infection, Malignancy and Autoimmunity (NIIMA), Universal Scientific Education and Research Network (USERN), Tehran, Iran; DN Department of Health Information Technology, Tabriz University of Medical Sciences, Tabriz, Iran; DO Department of Epidemiology and Biostatistics, Rafsanjan University of Medical Sciences, Rafsanjan, Iran; DP Golestan Research Center of Gastroenterology and Hepatology, Golestan University of Medical Sciences, Gorgan, Iran; DQ School of Medicine, Gonabad University of Medical Sciences, Gonabad, Iran; DR Cardiac Rehabilitation Research Center, Isfahan University of Medical Sciences, Isfahan, Iran; DS Department of Nursing and Midwifery, Saveh University of Medical Sciences, Saveh, Iran; DT Global Health Economy Research Team, Kiel Institute for the World Economy, Kiel, Germany; DU Endocrinology and Metabolism Population Sciences Institute, Tehran University of Medical Sciences, Tehran, Iran; DV Research Institute for Health Sciences and Environment, Shahid Beheshti University of Medical Sciences, Tehran, Iran; DW Department of Health in Disaster & Emergency, Shahid Beheshti University of Medical Sciences, Tehran, Iran; DX Clinical Research Development Center (CRDC), Qom University of Medical Sciences, Qom, Iran; DY Non-communicable Diseases Research Center (NCDRC), Tehran University of Medical Sciences, Tehran, Iran; DZ Center for Global Health Research, Saveetha University, Chennai, India; EA Biotechnology Research Center, Mashhad University of Medical Sciences, Mashhad, Iran; EB Department of Health Education & Promotion, A.C.S. Medical College and Hospital, Karaj, Iran; EC Research Center for Health, Safety and Environment, Alborz University of Medical Sciences, Karaj, Iran; ED Department of Immunology, Pasteur Institute of Iran, Tehran, Iran; EE Drug Applied Research Center, Tabriz University of Medical Sciences, Tabriz, Iran; EF Department of Neurology, Tehran University of Medical Sciences, Tehran, Iran; EG Department of Chemistry, Institute for Advanced Studies in Basic Sciences (IASBS), Zanjan, Iran; EH Faculty of Medicine, Tabriz University of Medical Sciences, Tabriz, Iran; EI Aging Research Institute, Tabriz University of Medical Sciences, Tabriz, Iran; EJ School of Medicine, Alborz University of Medical Sciences, Karaj, Iran; EK Department for Evidence-based Medicine and Evaluation, University for Continuing Education Krems, Krems, Austria; EL Department of Speech Therapy, Kermanshah University of Medical Sciences, Kermanshah, Iran; EM Cancer Research Center, Tehran University of Medical Sciences, Tehran, Iran; EN Cancer Biology Research Center, Tehran University of Medical Sciences, Tehran, Iran; EO Center for Technology and Innovation in Cardiovascular Informatics, Iran University of Medical Sciences, Tehran, Iran; EP Department of Medical-Surgical Nursing, Mazandaran University of Medical Sciences, Sari, Iran; EQ Department of Nursing and Health Sciences, Flinders University, Adelaide, SA, Australia; ER School of Medicine, Urmia University of Medical Sciences, Urmia, Iran; ES School of Medicine, Babol University of Medical Sciences, Babol, Iran; ET Department of Basic Medical Sciences, Islamic Azad University, Mashhad, Iran; EU Department of Internal Medicine, Islamic Azad University, Mashhad, Iran; EV Department of Health, Safety, and Environmental Management, Abadan School of Medical Sciences, Abadan, Iran; EW Department of Microbiology, Golestan University of Medical Sciences, Gorgan, Iran; EX Department of Biostatistics and Epidemiology, Shahid Sadoughi University of Medical Sciences, Yazd, Iran; EY Basic Sciences in Infectious Diseases Research Center, Shiraz University of Medical Sciences, Shiraz, Iran; EZ Department of Pathology, Tehran University of Medical Sciences, Tehran, Iran; FA Department of Clinical Biochemistry, Isfahan University of Medical Sciences, Isfahan, Iran; FB Department of Basic Medical Sciences, Neyshabur University of Medical Sciences, Neyshabur, Iran; FC Research Center of Physiology, Semnan University of Medical Sciences, Semnan, Iran; FD Centre for Public Health and Wellbeing, University of the West of England, Bristol, United Kingdom; FE Social Development and Health Promotion Research Center, Kermanshah University of Medical Sciences, Kermanshah, Iran; FF Nursing Care Research Center in Chronic Diseases, Ahvaz Jundishapur University of Medical Sciences, Ahvaz, Iran

## Abstract

**Background:**

Better evaluation of the contribution of the main diseases, injuries, and risk factors for mortality and life expectancy is crucial for more efficient policy making at the national and subnational levels in Iran. The aim of this study is to assess the effect of emerging causes of mortality on health, specifically COVID-19, which can help policy makers implement preventive measures in similar situations.

**Methods:**

In this systematic analysis of the Global Burden of Diseases, Injuries, and Risk Factors Study (GBD) 2023, we present estimates of cause-specific mortality at the national and subnational levels in Iran from 1990 to 2023. New to this iteration of GBD, we present a decomposition analysis of the contribution of specific causes of death to net gain or loss in life expectancy across 31 provinces of Iran. We used an array of data sources including censuses, vital registration, and surveys for national and subnational estimates.

**Findings:**

The two leading causes of death in Iran were ischaemic heart disease and stroke in both 1990 and 2019. However, in 2020 and 2021, the COVID-19 pandemic displaced the leading causes of death, ranking first with age-standardised mortality rates of 286·2 deaths (95% uncertainty interval 267·9–310·5) per 100 000 in 2020 and 250·0 deaths (233·2–272·5) per 100 000 in 2021. COVID-19 ranked second and tenth in 2022 and 2023, respectively. Life expectancy at birth for both sexes combined declined from 78·0 years (77·7–78·1) in 2019 to 74·3 years (74·0–74·4) in 2020. It steadily recovered to 78·8 years (78·5–79·2) in 2023. COVID-19 was the main cause of loss in life expectancy, by 4·19 years, between 2019 and 2020. There was a net gain of 12·4 years in life expectancy in Iran from 1990 to 2023. The net gain at the national level can be mostly attributed to reduced mortality from ischaemic heart disease (2·61 years), stroke (1·63 years), neonatal disorders (1·26 years), transport injuries (0·88 years), and neoplasms (0·64 years). The decline in mortality rates of major causes continued to 2023 despite the pandemic. An exception was Alzheimer's disease, which showed a 4·0% increase in rate between 2019 and 2023 and led to a net loss of 0·04 years in life expectancy since 1990. Diabetes led to a net loss of 0·09 years since 1990. There were variations between provinces in terms of age-standardised rates and the net change in life expectancy before and after the COVID-19 pandemic.

**Interpretation:**

The COVID-19 pandemic disrupted the rising trend of life expectancy in Iran, varying across provinces. Findings show that the health-care infrastructure and policies in Iran were not efficient in controlling the pandemic in 2020 and 2021, mainly due to inadequate vaccination coverage and timeliness, specifically for vulnerable subgroups. Sanctions may have aggravated the effect of COVID-19 on loss in life expectancy of Iranians. Despite the pandemic, the declining trend in age-standardised rates for top causes of mortality has continued to 2023, leading to a full recovery of life expectancy and underscoring the ultimate resilience of Iran's health system.

**Funding:**

Gates Foundation.

## Introduction

Iran, with a population of 87 925 314 in 2023, is the second most populous country in north Africa and the Middle East, after Egypt.^1^ Iran is also the 18th largest country in the world. The majority of Iranians are Muslims, and there are various ethnicities residing in various provinces with different cultures.^2^ After the Islamic revolution in 1979, an extensive primary health-care network was established in the early 1980s, which impressively improved health indicators.^3^ It is worth noting that social and economic developments during the past three decades had considerable contributions to improving health indicators.^1^, ^2^, ^4^ Additionally, the establishment of Universal Rural Health Insurance in 2005 and the Health Transformation Plan in 2014 further contributed to improvements in indicators.^5^, ^6^, ^7^ These plans aimed to achieve universal health coverage, improve financial protection of households and vulnerable populations specifically in rural areas, and ensure equity in access to health services.^5^, ^6^, ^7^ Consequently, Iran has already met the Sustainable Development Goals for neonatal, child, and maternal mortality in all 31 provinces. Iran is now undergoing an epidemiological transition.^8^, ^9^

**Research in context**

**Evidence before this study**

Burden of disease studies in Iran date back to 2009, when the first report on the burden of diseases and injuries for 2003 was published in *Population Health Metrics*. Later, in 2014, a series of papers on the national burden of diseases in Iran was published in *Archives of Iranian Medicine*, with results from the Global Burden of Diseases, Injuries, and Risk Factors Study (GBD) 2010. In 2017, the results of GBD 2015 on national burden in Iran were published in the same journal. A review paper was published in *The Lancet* in 2019 entitled Iran in Transition, in which a comprehensive picture of Iran and the history of its health-care system was presented. In 2022, a systematic analysis of GBD 2019 estimates was done, which was also published in *The Lancet*. The article provides a comprehensive overview of health system performance in Iran, at the national level and for the first time at the subnational level. We also found numerous local studies on the burden of COVID-19 in Iran in 2020 and 2021. Yet, no clear picture of COVID-19 epidemiology at the national and subnational levels in Iran has been published.

**Added value of this study**

The current study is the first report on GBD 2023 estimates of provincial variation and inequalities in life expectancy, cause-specific mortality, and years of life lost in Iran since 1990 using a hierarchy of mutually exclusive and collectively exhaustive causes of death. In this study, we provide a decomposition analysis of the contribution of cause-specific mortality to gains or losses in life expectancy across 31 provinces since 1990. Additionally, we provide a systematic calibration of COVID-19 burden and its contribution to the decrease in life expectancy at the national and subnational level across 31 provinces in 2020 and 2021. This analysis provides policy makers with information on inequality in distribution of the main causes of mortality across provinces of Iran.

**Implications of all the available evidence**

This study provides a full analysis of the changing patterns in cause-specific mortality and life expectancy, providing policy makers with a comprehensive picture of the distribution and contribution of COVID-19 to loss in life expectancy at the subnational level in Iran. This report provides essential information for priority setting in Iran's health system at the subnational level. The results of this study will help to enhance reduction in mortality by providing valuable estimates of gains and losses of life expectancy at the subnational level in Iran since 1990 and between 2020 and 2023.

Iran's performance in improving health outcomes is higher than expected based on a gross domestic product (GDP) per capita of just 18 441·6 purchasing power parity international dollars in 2024, which places Iran in the category of lower-middle-income countries as defined by the World Bank.^10^ Along with stricter sanctions since 2017, there was a continued decline in the current health expenditure per capita from US$464 in 2017 to $237 in 2022; and the percentage of the current health expenditure of the GDP dropped from 8·0% in 2016 to 5·3% in 2022,^11^, ^12^ which might counter the developments in health indicators that have been achieved during the past four decades. The percentage of general government health expenditure of the current health expenditure stayed steady at 49·2%. The share of out-of-pocket expenditure of the current health expenditure increased to 39·1% in 2022, which is considerably higher than the global estimate of 17·2%; this shows that Iran has not been completely successful in achieving universal health coverage as there are still large inequalities in health indicators across provinces with various wealth indices.^11^ Unfortunately, Iran's economy has been under pressure over the past 8 years due to extensive sanctions and the COVID-19 pandemic, which necessitates estimating their effects on the distribution and trends of cause-specific mortality rates and life expectancy.^13^, ^14^, ^15^, ^16^, ^17^, ^18^

The history of burden of disease studies in Iran dates back to 2009, when the first report on the national burden in 2003 was published.^19^, ^20^ The burden of diseases in Iran has been extensively investigated in previous rounds of the Global Burden of Diseases, Injuries, and Risk Factors Study (GBD).^21^, ^22^, ^23^, ^24^, ^25^, ^26^ A systematic analysis on health system performance in Iran was done based on GBD 2019 estimates.^27^ Yet, current gaps in our knowledge of the effects of COVID-19 and sanctions need to be addressed for better priority setting at subnational level. The current study is the first and most comprehensive one exploring the effect of COVID-19 on the health of Iranians and decomposing the separate contribution of cause-specific mortality to changes in life expectancy from 1990 to 2023. This manuscript was produced as part of the GBD Collaborator Network and in accordance with the GBD Protocol.

## Methods

### Overview

GBD 2023 provided estimates of the burden of 292 causes of death by age-sex-location-year for 25 age groups from birth to age 95 years and older; for males, females, and both sexes combined; in 204 countries and territories grouped into seven super-regions and 21 regions; and for every year from 1990 to 2023, with subnational estimates for 20 countries and territories, including Iran. Level 1 of the GBD framework organises causes into communicable, maternal, neonatal, and nutritional diseases (CMNN), non-communicable diseases (NCDs), and injuries. Level 2 disaggregates those categories into 22 clusters of causes, which are further divided into Level 3 and Level 4 causes. The detailed estimation framework of GBD 2023 has been discussed previously.^1^, ^4^

This research is reported in accordance with GATHER recommendations. Software packages used in the cause-of-death analysis for GBD 2023 were Python (version 3.10.4), Stata (version 13.1), and R (version 4.4.0). Statistical code used for GBD 2023 estimation is available upon request. Detailed statistical analytical methods are in appendix 1.

### Cause-specific mortality at national and subnational levels

The methods in this study follow those in GBD 2021.^1^, ^4^ GBD 2023 used several databases from Iran, retrieved from various sources, including censuses and vital registration, which are available on the GBD 2023 Global Health Data Exchange platform.^28^ We report estimates of cause-specific mortality for 2023 in terms of number of deaths and age-standardised death rates per 100 000 population. We calculated the age-standardised rates using the GBD 2023 standard population. We report 95% uncertainty intervals (UIs) for each measure, which were generated using the 2·5th and 97·5th ordered 250 draws of the posterior distribution. Briefly, cause-specific death rates were estimated for 214 causes using the Cause of Death Ensemble model (CODEm), and alternative strategies were used to model causes with few data, unusual epidemiology, or abrupt changes in reporting over the study period.^1^, ^4^ CODEm is a tool that was developed specifically for GBD and evaluates the out-of-sample predictive validity of different statistical models and covariate permutations. It combines the results from those evaluations to estimate cause-specific mortality.

### COVID-19 mortality estimations

Estimates for COVID-19 were made using an analysis of the overall excess mortality due to the pandemic from the beginning of January, 2020, to the end of December, 2023. In short, for GBD 2023, the misclassification of COVID-19 deaths was addressed with data from 188 country-years. Many COVID-19 deaths were incorrectly attributed to other causes, creating artificial mortality spikes. To identify and correct these, a support vector machine algorithm was used to detect deviations from expected pre-pandemic mortality trends. For each cause with a spike, correlations between excess mortality and COVID-19 death rates were analysed to distinguish true increases from misclassified cases. Expected deaths were estimated with two counterfactual models: a linear regression based on 2015–19 data and a global non-COVID-19 death rate model. Excess deaths attributed to misclassified COVID-19 were then reassigned to COVID-19 for more accurate accounting.^1^, ^4^

To estimate COVID-19 as a cause of death, GBD combined the corrected data with provisional vital registration data and surveillance data through the OneMod modelling tool. OneMod integrated multiple covariates, including infection and vaccination rates, variant prevalence, health-care access, and key comorbidities such as obesity, cardiovascular disease, diabetes, and chronic kidney disease. The model produced refined, age-specific and sex-specific COVID-19 mortality estimates across all regions, assuming full infection detection, thereby improving global mortality assessments. Details are provided in previous publications.^1^, ^4^

### Life expectancy decomposition

We used a decomposition analysis to estimate the cause-specific contributions to gain or loss in life expectancy. The aim of decomposing life expectancy is to analyse differences in life expectancy by age and province and to quantify the effect of cause-specific deaths on life expectancy. Detailed steps of the analysis are presented in previous publications.^4^ In the first step, the difference in life expectancy was broken down by age. In the second step, these contributions were further subdivided by age and cause. Using this analysis, the specific causes of death that contributed to the differences in life expectancy were identified within each age group. At the final step, these cause-age-specific contributions were cumulated across age groups to estimate the cause-specific contributions to the overall variation in life expectancy. We investigated the top 20 Level 2 and Level 3 GBD causes that contributed to the observed change in life expectancy.^4^

### Role of the funding source

The funder of this study had no role in study design, data collection, data analysis, data interpretation, or the writing of the report. Coauthors affiliated with the funder had the opportunity to provide feedback on initial maps and drafts of this manuscript.

## Results

### National estimates

The total number of deaths in Iran increased from 360 000 (95% UI 354 000–368 000) in 1990 to 378 000 (373 000–385 000) in 2019. There was an increase in the total number of deaths to 527 000 (519 000–537 000) in 2020. However, the number of deaths steadily declined back to 365 000 (352 000–378 000) in 2023. Almost 45·0% of all deaths in all years between 1990 and 2023 pertained to females.

The age-standardised mortality rate decreased from 1131·3 deaths (95% UI 1112·3–1151·9) per 100 000 in 1990 to 582·5 deaths (574·0–592·5) per 100 000 in 2019. There was also a sharp increase in rate to 835·7 deaths (822·4–851·3) per 100 000 in 2020. The rate again declined to 550·0 deaths (528·7–571·4) per 100 000 in 2023. The pattern was similar between sexes, although the rates were consistently lower in females (appendix 2 pp 5, 25–27). The share of premature deaths (under age 70 years) decreased from 76·2% in 1990 to 45·1% in 2019, 46·5% in 2020, and 46·7% in 2023 in both sexes.

Between 2020 and 2023, the pattern of leading causes of mortality was changed by COVID-19. Ischaemic heart disease and stroke were the first two causes of death in terms of age-standardised rate in both 1990 and 2019. Both causes of death showed a steady decline in rate since 1990. However, in 2020 and 2021, COVID-19 ranked first instead of ischaemic heart disease at the national level in Iran. COVID-19 ranked second in 2022 and tenth in 2023 (table 1). The pattern was similar between sexes (appendix 2 pp 28–32).

**Table 1:** Age-standardised mortality rates for the top 12 causes of death at national level in Iran in 2019, 2020, 2021, 2022, and 2023, in both sexes

	**2019**		**2020**		**2021**		**2022**		**2023**		**Percentage change in rate, 2019–23**
	Cause	Rate	Cause	Rate	Cause	Rate	Cause	Rate	Cause	Rate	
1	Ischaemic heart disease	142·9 (119·3–160·4)	COVID-19	286·2 (267·9–310·5)	COVID-19	250·0 (233·2–272·5)	Ischaemic heart disease	125·6 (102·6–146·6)	Ischaemic heart disease	127·2 (104·5–149·1)	−11·0%
2	Stroke	58·4 (49·0–67·9)	Ischaemic heart disease	140·5 (116·2–162·2)	Ischaemic heart disease	130·1 (106·5–152·1)	COVID-19	57·1 (50·9–65·5)	Stroke	47·1 (37·2–58·5)	−19·3%
3	Hypertensive heart disease	31·1 (24·2–39·3)	Stroke	56·1 (46·7–68·6)	Stroke	50·2 (40·6–61·9)	Stroke	47·6 (38·3–59·3)	Alzheimer's disease	31·0 (8·1–80·2)	4·0%
4	Alzheimer's disease	29·8 (7·6–77·8)	Hypertensive heart disease	31·3 (25·1–39·7)	Hypertensive heart disease	29·1 (22·7–37·3)	Alzheimer's disease	31·0 (8·0–80·8)	Hypertensive heart disease	28·1 (21·5–36·4)	−9·6%
5	COPD	21·7 (16·1–27·3)	Alzheimer's disease	25·1 (6·2–69·0)	Alzheimer's disease	27·1 (6·7–73·0)	Hypertensive heart disease	28·1 (21·8–36·1)	COPD	21·9 (15·1–29·9)	0·9%
6	Diabetes	21·6 (15·7–27·1)	Road injuries	21·4 (15·1–27·0)	Chronic kidney disease	19·9 (15·3–25·6)	COPD	21·6 (15·2–29·1)	Chronic kidney disease	20·5 (14·3–27·3)	−1·9%
7	Road injuries	21·5 (15·7–27·1)	Chronic kidney disease	21·1 (16·6–26·8)	Road injuries	19·8 (14·0–25·2)	Diabetes	21·0 (15·1–26·5)	Diabetes	20·4 (14·8–26·4)	−5·5%
8	Chronic kidney disease	20·9 (16·5–26·5)	Diabetes	18·2 (13·4–22·5)	Diabetes	19·2 (14·0–23·8)	Chronic kidney disease	19·8 (14·5–25·9)	Lower respiratory infections	18·7 (13·7–25·0)	−2·6%
9	Lower respiratory infections	19·2 (15–24·4)	COPD	16·8 (11·7–22·0)	COPD	17·9 (12·4–24·1)	Road injuries	18·4 (13·3–23·8)	Road injuries	18·3 (13·6–23·9)	−14·9%
10	Stomach cancer	15·9 (12·9–19)	Stomach cancer	15·8 (12·7–18·8)	Lower respiratory infections	14·8 (10·6–19·2)	Lower respiratory infections	18·3 (13·3–23·6)	COVID-19	17·3 (14·7–20·5)	..
11	Lung cancer	12·4 (10·5–15·1)	Lower respiratory infections	14·2 (10·5–18·3)	Stomach cancer	14·5 (11·5–17·3)	Stomach cancer	14·2 (10·9–17·4)	Stomach cancer	14·1 (10·7–17·4)	−11·3%
12	Neonatal disorders	11 (10·2–12)	Lung cancer	12·5 (10·8–15·0)	Lung cancer	11·6 (10·0–13·9)	Lung cancer	11·5 (9·6–13·9)	Lung cancer	11·6 (9·6–14·1)	−6·5%

Data are rate (95% uncertainty intervals), unless otherwise specified. COPD=chronic obstructive pulmonary disease.

In 2020 and 2021, ischaemic heart disease ranked second, and stroke ranked third in terms of age-standardised rate. Both causes of death retained their high rank in 2023. Alzheimer's disease, hypertensive heart disease, chronic obstructive pulmonary disease (COPD), chronic kidney disease, diabetes, lower respiratory infections, and road injuries ranked third to ninth in 2023, maintaining the order they had in 2019 (table 1; appendix 2 pp 28–32). The steady decline in rates of all top causes of death continued to 2023 despite the COVID-19 pandemic, with the exception of Alzheimer's disease. Between 2020 and 2023, the largest declines in age-standardised rate were observed for stroke (–19·3%), road injuries (–14·9%), stomach cancer (–11·3%), ischaemic heart disease (–11·0%), hypertensive heart disease (–9·6%), lung cancer (–5·5%), and diabetes (–5·5%). The largest increase in rate was observed for Alzheimer's disease (4·0%; table 1).

Certain differences between females and males were observed in the rank of specific causes of death. Among females, Alzheimer's disease ranked higher whereas road injuries ranked lower, and breast cancer in females displaced lung cancer in males. COPD ranked higher in males. The order of other causes and the percentage changes were almost similar between the two sexes (appendix 2 pp 28–32).

### Life expectancy at birth and decomposition for all causes

**Figure 1:**
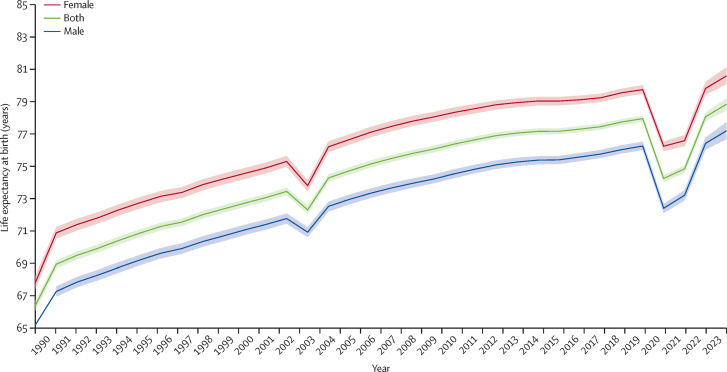
Life expectancy trends at birth at the national level in Iran, 1990–2023 By sex (male, female, both) with uncertainty intervals (shaded areas).

Life expectancy at birth for both sexes combined increased from 66·4 years (95% UI 66·1–66·7) in 1990 to 78·0 years (77·8–78·1) in 2019 (figure 1). There was a dip in life expectancy in 1990 and 2003 due to earthquakes. Yet, life expectancy declined to 74·3 years (74·0–74·4) in 2020 and 74·8 years (74·6–75·1) in 2021, which was observed in all provinces in Iran and was mainly due to COVID-19. However, in 2023, life expectancy showed an increase to 78·8 years (78·5–79·2). Life expectancy at birth across 31 provinces in 2020 and 2023 is in appendix 2 (p 6). In 2020, life expectancy at birth ranged from 70·6 years (70·3–71·0) in the poorest province of Sistan and Baluchistan to 76·1 years (75·9–76·3) in the richest province of Tehran. Respective figures in 2023 were 74·8 years (74·3–75·4) in Zanjan and 81·5 years (81·0–81·9) in Tehran (appendix 2 pp 6, 33–35). Life expectancy was higher in females at the national level and across all provinces between 1990 and 2023 (figure 1).

There was a net gain of 12·4 years in life expectancy in Iran from 1990 to 2023 (appendix 2 p 7). The net gain since 1990 ranged from 6·4 years in Alborz to 16·1 years in Sistan and Baluchistan. Larger gains were observed in Gilan and Zanjan due to the effect of the earthquake on life expectancy in 1990 in these two provinces. The net gain at the national level can be mostly attributed to reduced mortality from ischaemic heart disease (2·61 years), stroke (1·63 years), neonatal disorders (1·26 years), transport injuries (0·88 years), neoplasms (0·64 years), congenital birth defects (0·52 years), lower respiratory infections (0·48 years), digestive diseases (0·36 years), enteric infections (0·30 years), hypertensive heart disease (0·24 years), diarrhoeal diseases (0·24 years), and cirrhosis (0·23 years). There was a net loss in life expectancy attributed to diabetes (–0·09 years) and Alzheimer's disease and other dementias (–0·04 years) between 1990 and 2023. The net gain attributed to ischaemic heart disease, stroke, congenital birth defects, and lower respiratory infections was larger in females, whereas the gain attributed to transport injuries and hypertensive heart disease was larger in males. The net loss due to diabetes was larger in males (–0·17 years) compared with females (–0·02 years). The net loss attributed to Alzheimer's disease was similar between sexes (appendix 2 pp 7–9).

### Effects of COVID-19 on mortality and life expectancy

COVID-19 caused 182 400 (95% UI 171 400–196 000) deaths in 2020, 168 500 (158 100–181 700) in 2021, 35 600 (32 100–40 100) in 2022, and 10 000 (8700–11 600) in 2023. The share of deaths among females out of all deaths due to COVID-19 was 44·4% in 2020, 47·3% in 2021, 50·0% in 2022, and 51·5% in 2023 (appendix 2 pp 36–38).

The age-standardised mortality rate due to COVID-19 in both sexes combined was 286·2 per 100 000 (267·9–310·5) in 2020, 250·0 (233·2–272·5) in 2021, 57·1 (50·9–65·5) in 2022, and 17·3 (14·7–20·5) per 100 000 in 2023. The age-standardised rate ranged from 249·7 per 100 000 (209·4–292·3) in Kurdistan to 330·6 per 100 000 (279·9–391·5) in Golestan in 2020. Respective figures for 2021 were lowest in Chaharmahal and Bakhtiari (213·6 [174·6–255·8]) per 100 000 and highest in Golestan (301·7 [260·7–356·6]) per 100 000 (table 2). In all years and all provinces, the age-standardised rate of death due to COVID-19 was lower in females in comparison with males (appendix 2 pp 39–42).

**Table 2:** The age-standardised rates of COVID-19 in 2020, 2021, 2022, and 2023, and its contribution to change in life expectancy in both sexes

	**SDI in 2019**	**COVID-19 age-standardised mortality rate per 100 000 population**				**Life expectancy**	**Change in life expectancy**		
		2020	2021	2022	2023	2019	2019–20	2020–23	2019–23
Tehran	0·78	268·1 (231·6–301·9)	235·0 (203·1–265·4)	55·3 (43·8–67·2)	17·2 (12·9–22)	80·3 (80·1–80·5)	−4·43	4·46	0·03
Alborz	0·74	293·6 (245·4–354·0)	267·2 (226·9–318·6)	65·0 (49·1–84·5)	20·2 (14·5–28·9)	79·1 (78·9–79·3)	−4·26	4·12	−0·15
Mazandaran	0·73	304·6 (254·9–359·6)	257·2 (216·4–305·3)	61·0 (47·5–79·6)	18·9 (13·8–26·0)	78·9 (78·6–79·1)	−4·14	4·22	0·08
Isfahan	0·72	283·1 (238·5–325·1)	248·4 (208·4–288·4)	58·8 (45·4–74·4)	18·0 (13·8–23·5)	79·1 (78·9–79·4)	−4·22	4·14	−0·08
Fars	0·71	309·8 (265·3–354·7)	259·9 (220·7–297·6)	62·4 (49·5–76·7)	19·4 (13·8–25·7)	78·8 (78·6–79·0)	−4·68	4·71	0·02
Gilan	0·71	313·8 (257·6–365·1)	277·3 (228·1–325·5)	66·6 (51–86·6)	20·8 (14·9–28·5)	78·1 (77·9–78·3)	−4·13	4·22	0·09
Semnan	0·71	306·8 (259·8–355·6)	268·1 (226·5–311·4)	63 (50·6–79·1)	19·3 (14·6–25·4)	78·6 (78·3–78·9)	−4·52	4·41	−0·11
Bushehr	0·70	304·2 (252·1–359·5)	265·7 (225·5–314·7)	61·7 (47·9–77·4)	18·7 (13·7–24·8)	77·6 (77·3–77·8)	−3·97	3·93	−0·03
Yazd	0·70	300·6 (254·1–350·7)	262·2 (221·2–305·0)	61·0 (47·5–76·7)	18·7 (13·8–24·8)	78·5 (78·2–78·8)	−4·43	4·34	−0·08
Ilam	0·69	307·6 (256·1–361·6)	270·3 (222·8–317·2)	66·5 (50·7–85·4)	20·8 (14·8–28·2)	78·7 (78·5–79·0)	−4·89	4·63	−0·26
Iran	0·69	286·2 (267·9–310·5)	250·0 (233·2–272·5)	57·1 (50·9–65·5)	17·3 (14·7–20·5)	77·9 (77·8, 78·1)	−4·19	4·17	−0·02
Qazvin	0·69	296·7 (251·9–349·5)	258·9 (215·8–305·2)	58·9 (45·1–75·5)	17·7 (12·7–23·1)	77·8 (77·5–78·0)	−4·21	4·11	−0·1
Markazi	0·68	265·9 (223·5–309·4)	237·8 (198·8–276·8)	54·2 (41·5–69·0)	16·3 (11·6–21·2)	78·4 (78·1–78·6)	−3·95	3·94	−0·01
Qom	0·68	296·4 (234·7–399·3)	260·4 (211·2–342·6)	56·2 (41·6–84·0)	16·8 (11·9–26·7)	75·7 (75·5–76·0)	−2·78	2·82	0·04
Kerman	0·67	303·9 (259·9–355·4)	266·2 (225·3–310·5)	61·2 (48·3–80·0)	18·6 (13·5–26·2)	77·5 (77·2–77·7)	−4·29	4·24	−0·05
Kermanshah	0·67	294·8 (245·7–346·8)	249·9 (203·6–297·5)	55·2 (41·5–70·4)	16·2 (11·7–22·1)	76·3 (76·1–76·6)	−4·08	4·1	0·02
Khorasan-e-Razavi	0·67	292·8 (247·3–339·3)	261·0 (224·2–305·6)	57·8 (45·4–72·3)	16·9 (12·6–22·4)	77·5 (77·3–77·8)	−4·34	4·35	0·01
Kohgiluyeh and Boyer-Ahmad	0·67	255·2 (217·6–294·5)	226·7 (193·6–265·6)	53·6 (40·4–69·5)	16·3 (11·4–22·3)	79·1 (78·8–79·4)	−4·74	4·59	−0·16
East Azarbayejan	0·66	302·5 (251·2–361·5)	264·5 (219·1–315·9)	58·4 (45·3–74·8)	17·4 (12·5–24·0)	76·5 (76·3–76·7)	−3·64	3·61	−0·03
Hamadan	0·66	288·8 (243·7–331·8)	245·1 (205·3–289·5)	54·0 (42·0–69·2)	15·8 (11·8–21·1)	77·4 (77·1–77·7)	−4·42	4·37	−0·05
Hormozgan	0·66	264·2 (222·2–306·3)	225·7 (186·8–260·0)	52·3 (40·0–66·7)	15·7 (11·8–20·5)	78·6 (78·4–78·9)	−4·8	4·66	−0·14
Khuzestan	0·66	273·5 (231·6–321·3)	240·2 (201·1–279·3)	51·7 (41·0–66·2)	14·9 (11·0–19·6)	76·7 (76·4–77·0)	−4·34	4·27	−0·07
Lorestan	0·66	286·1 (239·6–342·5)	244·1 (203·2–294·9)	55·2 (41·9–71·3)	16·6 (12·1–22·9)	76·6 (76·3–76·8)	−3·74	3·74	0
Ardebil	0·65	295·2 (244·2–350·5)	254·8 (211·5–301·6)	56·4 (43·0–71·0)	16·7 (12·1–21·8)	76·9 (76·7–77·2)	−4·11	4·05	−0·06
Chahar Mahaal and Bakhtiari	0·65	254·1 (209·0–303·6)	213·6 (174·6–255·8)	49·7 (37·9–63·9)	15·3 (11·0–21·1)	78·0 (77·8–78·3)	−3·62	3·59	−0·04
Zanjan	0·65	309·9 (252·2–370·1)	282·5 (233·0–335·8)	60·7 (45·9–78·1)	17·7 (12·6–23·9)	74·4 (74·2–74·7)	−3·36	3·27	−0·09
Golestan	0·64	330·6 (279·9–391·5)	301·7 (260·7–356·6)	67·6 (53·5–86·9)	19·8 (14·8–26·8)	77·1 (76·9–77·4)	−5·09	5·04	−0·04
South Khorasan	0·64	289·5 (245·6–340·1)	251·2 (211·9–293·8)	57·3 (43·5–72·5)	16·9 (12·3–23·1)	78·5 (78·2–78·7)	−4·05	4·03	−0·02
Kurdistan	0·63	249·7 (209·4–292·3)	221·0 (185·8–259·2)	49·2 (36·7–62·8)	14·5 (10·8–19·0)	77·8 (77·5–78·0)	−3·99	3·82	−0·17
North Khorasan	0·63	320·5 (270·3–380·2)	291·8 (247·3–349·3)	62·4 (49·3–78·9)	17·8 (13·4–24·0)	76·3 (76·0–76·5)	−4·32	4·29	−0·03
West Azarbayejan	0·63	270·7 (223·4–330·2)	228·9 (185·7–277·5)	49·0 (37·9–65·0)	14·5 (10·5–20·9)	76·4 (76·1–76·6)	−3·51	3·44	−0·07
Sistan and Baluchistan	0·53	271·6 (226·3–315·0)	243·6 (207·7–281·0)	48·7 (38·5–59·9)	13·4 (10·1–17·7)	74·9 (74·5–75·2)	−4·66	4·78	0·13

Provinces are sorted based on their SDI in 2019. SDI=Socio-demographic Index.

COVID-19 mortality led to a net loss of 4·19 years at the national level in 2020. The net loss was observed in all provinces, ranging from 2·78 years in Qom to 5·09 years in Golestan (table 2). However, life expectancy substantially recovered between 2020 and 2023. The details of life expectancy decomposition for COVID-19 across Iran and the 31 provinces between 2019 and 2023 are shown in table 2. The decomposition by sex showing minimal difference is shown in appendix 2 (pp 39–42).

### Effects of CMNN diseases on mortality and life expectancy

Age-standardised mortality rates for the main childhood diseases and infections are presented in appendix 2 (pp 10–12). The age-standardised mortality rate for lower respiratory infections was 18·7 per 100 000 (95% UI 13·7–25·0) in Iran in 2023, ranging from 8·6 (5·6–12·6) in Ardebil to 36·4 (22·6–58·7) per 100 000 in Qom. Congenital birth defects, with an age-standardised rate of 9·0 per 100 000 (7·6–10·3) at the national level, ranged from 6·2 (5·1–7·3) in Alborz to 11·5 (9·7–14·3) per 100 000 in Kermanshah. The rates for neonatal disorders were 8·9 (7·9–10·2) per 100 000 at the national level, lowest in Mazandaran (5·7 [4·7–6·7]) and highest in Kermanshah (13·7 [11·6–15·7]) per 100 000. The order of diseases was quite similar between females and males (appendix 2 pp 11–12).

**Figure 2:**
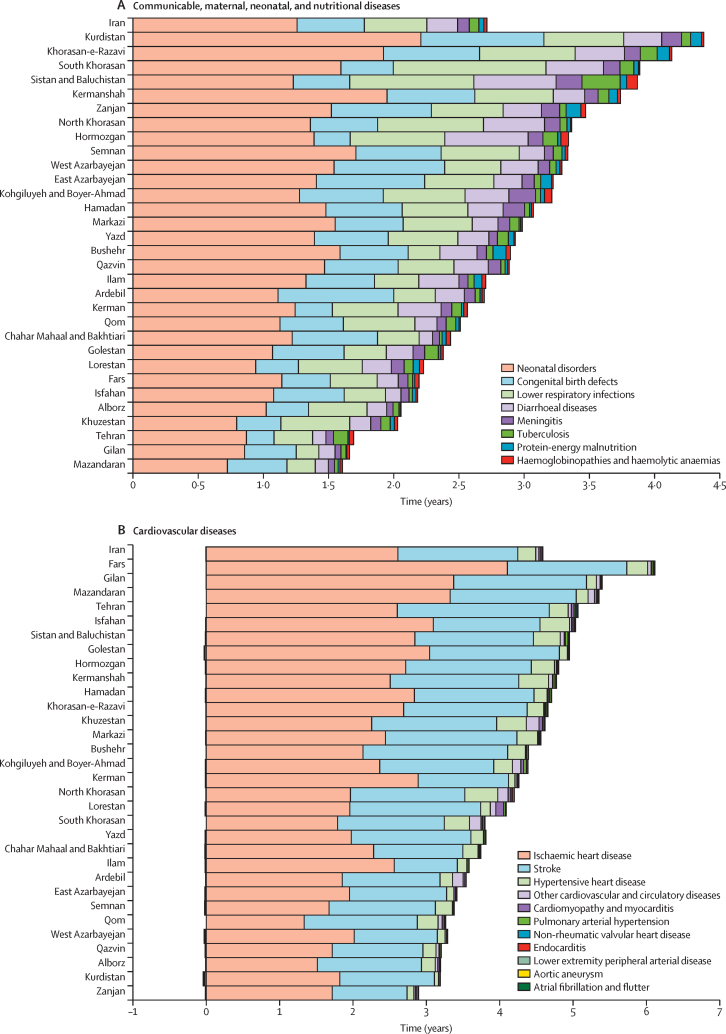

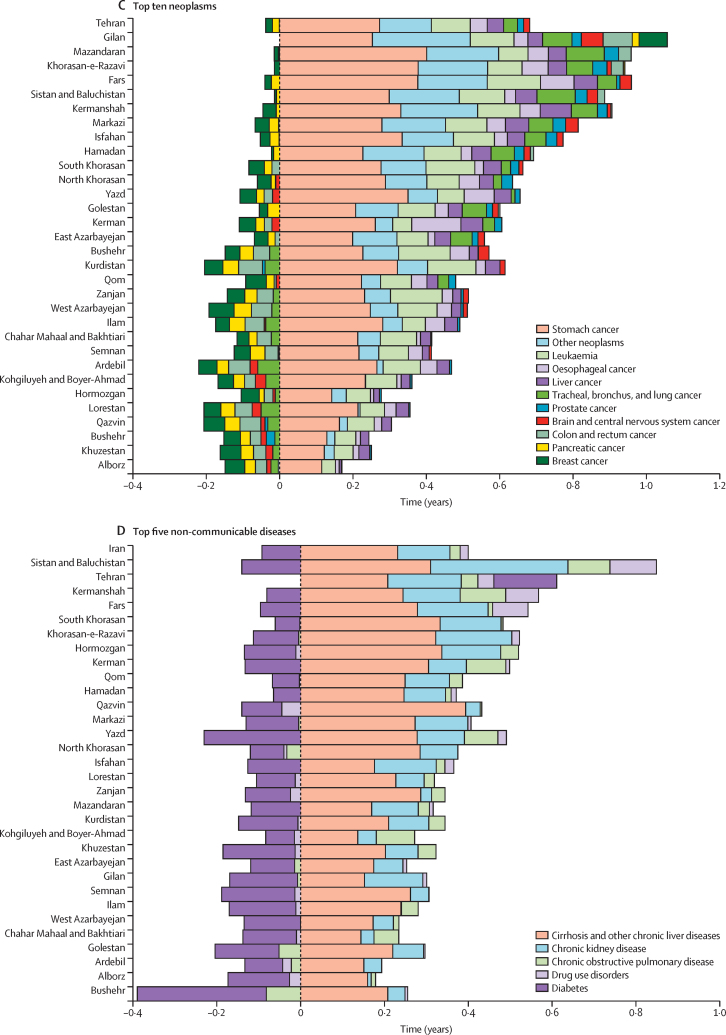

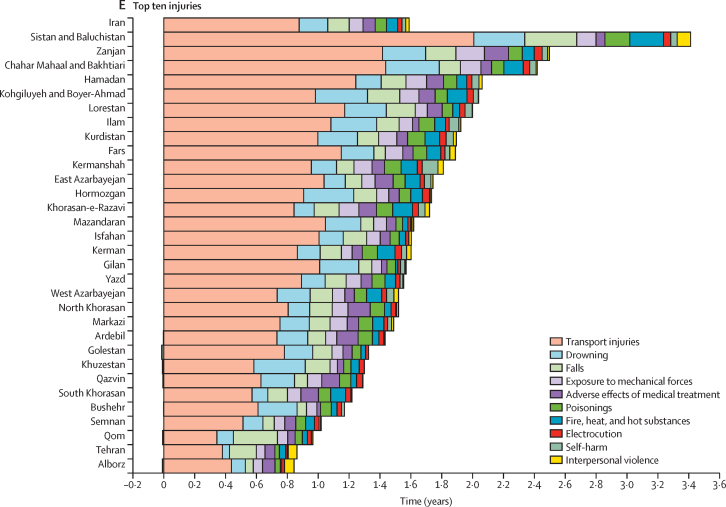
Life expectancy decomposition for all causes across Iran and the 31 provinces, 1990–2023

At the national level, a net gain in life expectancy of 1·26 years was observed, attributed to reduction in mortality rates of the main neonatal disorders from 1990 to 2023, ranging from 0·72 years in Mazandaran to 2·21 years in Kurdistan (figure 2A; appendix 2 pp 43–45). There was a net gain of 0·52 years in life expectancy at the national level, attributed to reductions in mortality due to congenital birth defects, ranging from 0·21 years in Tehran to 0·94 years in Kurdistan. The net gain in life expectancy was attributed to reductions in mortality due to lower respiratory infections (0·48 years), diarrhoeal diseases (0·24 years), meningitis (0·09 years), tuberculosis (0·07 years), protein-energy malnutrition (0·04 years), and haemoglobinopathies and haemolytic anaemias (0·02 years). The net gain attributed to congenital birth defects, lower respiratory infections, and meningitis was larger in females (appendix 2 pp 44–45).

### Effects of NCDs on mortality and life expectancy

At Level 3 causes of death, the estimated age-standardised mortality rate due to ischaemic heart disease was 127·2 per 100 000 (95% UI 104·5–149·1) at the national level in Iran in 2023, ranging from 88·1 (67·2–108·3) in Tehran to 262·8 (213·4–310·5) per 100 000 in Qom. Stroke ranked second with an age-standardised mortality rate of 47·1 (37·2–58·5) per 100 000, ranging from 34·4 (26·4–44·6) in Ilam to 79·1 (54·1–100·7) per 100 000 in Qom (appendix 2 pp 13–15). The age-standardised rates for hypertensive heart disease were 28·1 (21·5–36·4) in Iran, 11·1 (4·5–26·4) in Tehran, and 56·3 (31·8–76·6) per 100 000 in East Azarbaijan. Rheumatic heart disease had higher rank in females while the rank of aortic aneurysm was higher in males (appendix 2 pp 14–15).

There was a net gain of 4·65 years at the national level in Iran since 1990 due to reductions in cardiovascular disease mortality (figure 2B; appendix 2 pp 46–48). The net gain ranged from 2·95 years in Zanjan to 6·18 years in Fars. There was a net gain of 2·61 years in life expectancy attributed to reductions in ischaemic heart disease mortality at the national level, ranging from 1·33 years in Qom to 4·11 years in Fars. A net gain of 1·63 years in life expectancy was estimated, attributed to improvements in stroke mortality at the national level. Reductions in mortality led to a net gain in life expectancy for hypertensive heart disease (0·24 years), rheumatic heart disease (0·07 years), other cardiovascular diseases (0·04 years), and cardiomyopathy and myocarditis (0·02 years). The net gain was observed in all provinces in 2023. All cardiovascular disease subgroups at Level 4 of the cause hierarchy led to gains in life expectancy at the national level, except for atrial fibrillation and flutter and aortic aneurysm. The net gain attributed to ischaemic heart disease and stroke was larger among females, whereas the gain due to hypertensive heart disease was larger among males (appendix 2 pp 47–48).

Neoplasms ranked first to tenth in terms of age-standardised rates at the national level in 2023 include cancers of the stomach, lung, and colon-rectum; leukaemia; and prostate, brain, breast, pancretic, oesophageal, and liver cancer (appendix 2 p 16). Neoplasms showed a high age-standardised rate in Iran (91·6 per 100 000 [95% UI 80·1–102·9]), lowest in Hormozgan and highest in Zanjan. The age-standardised mortality rate due to stomach cancer was lowest in Hormozgan (7·6 [5·0–12·3]) and highest in Zanjan (28·6 [20·1–37·2]). Mortality rates for lung cancer were lowest in Fars (8·4 [7·0–10·5]) and highest in West Azarbaijan (18·9 [13·3–23·0]). As for colorectal cancer, Kohgiluyeh and Boyer-Ahmad had the lowest rate, whereas Qom had the highest rate. Estimates show that prostate cancer shifted down the order of cancers among males and breast cancer showed the same pattern among females. The order of other cancers was almost similar between the two sexes (appendix 2 pp 17–18).

There was a net gain of 0·64 years in life expectancy since 1990 attributed to reductions in overall mortality due to all neoplasms (figure 2C; appendix 2 p 49). The gain ranged from 0·02 years in Alborz to 1·06 years in Tehran. Change in mortality rates due to stomach cancer, leukaemia, oesophageal cancer, and liver cancer led to net gains in life expectancy in all provinces of Iran. Change in mortality rates of lung cancer, prostate cancer, and brain cancer led to gains in life expectancy at the national level as well as several provinces but led to loss in others. Colorectal cancer, breast cancer, and pancreatic cancer led to a net loss in life expectancy at the national level (figure 2C; appendix 2 p 49). There was no significant difference in net gain or loss attributed to cancers between females and males (appendix 2 pp 50–51).

The age-standardised mortality rate due to COPD was 21·9 per 100 000 (95% UI 15·1–29·9) in 2023, ranging from 10·2 (6·6–16·2) in Kohgiluyeh and Boyer-Ahmad to 48·8 (28·7–66·9) per 100 000 in Kerman (appendix 2 p 19).

The age-standardised mortality rate in 2023 at the national level was 20·5 per 100 000 (95% UI 14·3–27·3) for chronic kidney disease, ranging from 16·2 (10·6–23·2) in Tehran to 35·6 (20·2–52·6) in Qom. As for diabetes, rates were 20·4 per 100 000 (14·8–26·4) at the national level, lowest in Kohgiluyeh and Boyer-Ahmad (10·5 [6·8–15·5]) and highest in Khuzestan (28·9 [18·7–40·0]) per 100 000. Among females, chronic kidney disease ranked first while among males, the first rank belonged to COPD (appendix 2 pp 20–21). The age-standardised rates at the national level were 10·1 per 100 000 (7·3–14·1) for cirrhosis and 3·1 per 100 000 (2·4–4·2) for drug use disorders.

The net gain in life expectancy attributed to decline in mortality rates of the five main NCDs since 1990 was 0·31 years at the national level in Iran, ranging from a net loss of 0·13 years in Bushehr to a net gain of 0·71 years in Sistan and Baluchistan (figure 2D; appendix 2 p 52). Declines in cirrhosis mortality led to a net gain of 0·23 years at the national level. Drug use disorders contributed to a net gain of 0·02 years. Declines in mortality rates of chronic kidney disease and COPD led to a net gain of 0·12 and 0·02 years, respectively, since 1990. Declines in chronic kidney disease and cirrhosis mortality rates led to increases in life expectancy across all provinces since 1990. The net gain attributed to chronic kidney disease was larger in males (appendix 2 pp 53–54).

Diabetes led to a net loss of 0·09 years in life expectancy since 1990 at the national level. Patterns varied substantially across provinces (figure 2D; appendix 2 p 52). The loss in life expectancy caused by diabetes was observed across all provinces except for Tehran. The loss due to diabetes was also larger among males (appendix 2 pp 53–54).

### Effects of injuries on mortality and life expectancy

Transport injuries had an age-standardised mortality rate of 18·9 per 100 000 (95% UI 14·1–24·7) in 2023, ranging from 6·5 per 100 000 (3·8–12·7) in Tehran to 42·8 per 100 000 (27·8–56·7) in Sistan and Baluchistan. Details for other types of injury are shown in appendix 2 (p 22). The age-standardised mortality rate due to transport injuries was 27·6 (18·5–38·3) per 100 000 among males and 10·1 (6·5–14·4) per 100 000 among females (appendix 2 pp 23–24).

Reduction in the age-standardised mortality rate of transport injuries contributed to a net gain of 0·88 years to life expectancy at the national level in Iran since 1990. The net gain ranged from 0·34 years in Qom to 2·01 years in Sistan and Baluchistan. A large part of the net gain was attributed to decline in road injuries mortality (figure 2E; appendix 2 p 55). The net gain attributed to transport injuries among males (1·17 years) was much higher than females (0·57 years; appendix 2 pp 56–57).

## Discussion

To our knowledge, this study represents the most comprehensive examination of the effects of COVID-19 on the health of Iranians, as well as the distinct contributions of cause-specific mortality to variations in life expectancy from 1990 to 2023. Our findings show the trend and inequalities in life expectancy and cause-specific mortality at the national and subnational level in Iran before and after the emergence of COVID-19.^30^ The pandemic abruptly reversed three decades of improvement in life expectancy in Iran.

The deaths caused by COVID-19 could have been partly prevented if vaccination coverage had been adequate and timely and had been equally implemented across 31 provinces.^31^ The combination of significant delay in vaccination, inadequate access to vaccination, and the negative attitude towards vaccination at the population level led to consecutive waves of COVID-19 in Iran.^32^, ^33^, ^34^ Evidence shows that the COVID-19 pandemic reduced the utilisation and quality of essential health services in Iran.^35^, ^36^ Sanctions aggravated the effect of the COVID-19 pandemic on the health system and human resources.^37^ Consequently, Iran, with an age-standardised death rate of 250 per 100 000 in 2021 caused by COVID-19, was among the countries with the highest COVID-19 death rates, higher than the rate in north Africa and the Middle East, at 179·9 per 100 000 (95% UI 167·6–191·6), and much higher than the global rate of 110·5 per 100 000 (105·9–113·9).^1^ Despite this disruption, a rapid decline in COVID-19 mortality and sustained reductions in CMNN and NCD deaths contributed to a full recovery in life expectancy by 2023, which underscores the resilience of Iran's health system and the effectiveness of subsequent national mitigation efforts.

Beyond the pandemic, Iran's mortality profile continues to reflect an advanced stage of epidemiological transition. We observed that the declining trend in age-standardised mortality continued for most causes despite the emergence of COVID-19 in almost all provinces of Iran. Deaths due to CMNN diseases have markedly declined, whereas NCDs remain the dominant causes of death. Among the top causes of mortality in 2023, apart from lower respiratory infections and transport injuries, NCDs constituted the rest of the top mortality causes, among which the increase in the rate of Alzheimer's disease is noteworthy.

There was a substantial gain in life expectancy due to decreases in CMNNs. Our findings show the effectiveness of the health-care system in reducing neonatal disorders in most provinces, including the low-income province of Kurdistan,^9^ whereas success was less prominent in high-income provinces such as Tehran and Mazandaran. Kurdistan shows the largest net gain in life expectancy due to reduction in neonatal mortality (2·21 years). Previous studies have shown the inequality in distribution of neonatal mortality across provinces of Iran.^9^, ^30^, ^38^, ^39^, ^40^ It appears that Iran's health-care system has been successful in reducing neonatal mortality; however, inequality still exists.

The rates of lower respiratory infections also showed decline since 1990, which continued to 2023. Lower respiratory infections was the only communicable disease among the top 12 causes of mortality. However, the contribution of reductions in lower respiratory infections to net gain in life expectancy (0·48 years) is not large and is similar across provinces.

The most notable life expectancy gains since 1990 were attributable to reductions in cardiovascular disease mortality—particularly ischaemic heart disease and stroke—which together contributed to over 4 years of life expectancy gain nationally. However, cardiovascular disease mortality remains elevated in many provinces.

It is generally assumed that deaths due to cardiovascular diseases might be more common in urban areas.^41^ However, the high rates observed in rural areas might be due to low access to timely health care.^42^, ^43^ Further investigation is needed to explore whether health-care use for cardiovascular disease prevention and treatment should be enhanced in rural areas of Iran.^44^, ^45^, ^46^ Health literacy and social determinants of health might be other possible predictors of health-care use for cardiovascular disease in rural areas of Iran.^47^, ^48^ Strengthening rural health infrastructure, ensuring equitable access to emergency and preventive care, and expanding screening for hypertension and dyslipidaemia should remain priorities.

Decline in rates of overall neoplasms led to a very modest increase in life expectancy (0·64 years). Neoplasms show declines in their age-standardised rate at the national level. However, the subnational variation likely reflects differences in health-care access, environmental exposures, and referral patterns to major treatment centres. Ambient air pollution and exposure to carcinogens^49^, ^50^ might be the causes of high rates in urban areas, whereas referral to large cities for treatment might be the cause of the low share in low-income provinces.

Reductions in deaths from stomach cancer led to a net gain in life expectancy in all provinces of Iran. This is in agreement with many other countries.^4^ Stomach cancer has higher age-standardised mortality rates in the northwest of Iran, which might be attributable to higher prevalence of *Helicobacter pylori*.^51^, ^52^

Change in mortality rates of lung cancer, prostate cancer, and brain cancer led to gains in life expectancy at the national level as well as several provinces but led to loss in others. Increases in deaths from colorectal cancer, breast cancer, and pancreatic cancer led to a net loss in life expectancy at the national level. These findings show the defect of the health-care system in Iran in prevention and early detection of preventable cancers such as lung cancer, breast cancer, and colorectal cancers.^53^ Effective strategies for prevention include initiatives to raise awareness about cancer risk factors and the importance of screening, to enhance access to health-care services in rural and low-income areas for timely diagnosis and treatment, to exert stricter environmental regulations to reduce air pollution and exposure to carcinogens, along with community-based programmes to promote healthy lifestyles, and to improve data collection and monitoring through robust cancer registries.

Our findings show the rising age-standardised rate of Alzheimer's disease during the pandemic in Iran, although such a rise is observed neither at the regional nor at the global level. There is growing evidence that SARS-CoV-2 infection can increase the risk of longer-term cognitive problems and is associated with a higher incidence of new-onset dementia, especially after severe COVID-19 and hospitalisation, and specifically among older adults.^54^, ^55^, ^56^ However, the causal association is not yet settled. The rise in the rate of Alzheimer's disease in Iran might be attributable to higher rates of COVID-19 and its severity in this country, compared with other regional and global estimates.

The decline in age-standardised mortality rates led to net gains in life expectancy for the top NCDs except for diabetes, which negatively affected life expectancy at birth since 1990.^57^ The high burden might be due to unhealthy lifestyle including low physical activity and unhealthy diet,^58^, ^59^ which could lead to increased prevalence of overweight and obesity.^60^, ^61^ The high rate could also be attributable to air pollution.^50^ Tehran was the only province in which changes in deaths from diabetes has actually led to a net gain in life expectancy (0·15 years).^62^, ^63^ Better access to treatment options and increases in awareness, income, and literacy during the past three decades might be the underlying justifications, but the possible explanations seem to be insufficient. Findings, in any case, show that the health-care system needs effective policies to prevent and correctly treat diabetes in all provinces and specifically in the capital of Iran.

Finally, transport injuries have a specifically high burden in Sistan and Baluchistan and, in general, in provinces with large area and inadequate road infrastructure.^64^ Transport injuries are considerably lower in Tehran.^65^ These findings highlight the necessity of improving road infrastructure and the quality of vehicles as well as seatbelt and helmet use in provinces where the majority of the residents are rural dwellers.^66^, ^67^

### Limitations

This study is the most comprehensive on the burden of main causes of death at the provincial level in Iran from 1990 to 2023. The limitations of this study are similar to those of the overall GBD 2023,^1^, ^4^, ^29^ including data sparsity and unreliability, the low quality of cause of death and verbal autopsy data, the high percentage of garbage coding, sources of uncertainty including covariates not captured in the models, alternative approaches adopted for certain causes of death, the dependence of estimates for recent years on modelling processes, instability in calculating life expectancy decomposition in cases when the difference in all-cause deaths is very small, and finally, the limitations of our methods for calculating the burden of COVID-19, which has been reported in previous publications.

### Conclusions

Despite the achievements made in reducing the main CMNNs and injuries so far at the national level, there is still considerable inequality between provinces. Policies have not been quite successful in controlling NCDs, specifically diabetes, cardiovascular disease, and certain neoplasms. The rising rate of Alzheimer's disease should be noted in the context of the ageing population in Iran. Different policies should be tailored to each province, the capital province of Tehran and other low-income provinces, depending on local health priorities. Investigating the amount, type, and venues of resource allocation at the provincial level can effectively and simultaneously elucidate the health-care system's defects and the possible solutions at the local level. Inter-sectoral collaboration between stakeholders can lead to achievements in reducing poverty and enhancing literacy and health-care access, coverage, and utilisation at the provincial level.

#### GBD 2023 Iran Collaborators

#### Affiliations

#### Contributors

#### Data sharing

To download the data used in these analyses, please visit the Global Health Data Exchange at https://ghdx.healthdata.org/gbd-2023.

## Declaration of interests

Hamid Reza Marateb reports grants or contracts from Universitat Politècnica de Catalunya–BarcelonaTech, outside the submitted work. All other authors declare no competing interests.
