# Supplementary material for: Burden of 292 causes of death and life expectancy decomposition in Iran, 1990–2023: a systematic analysis for the Global Burden of Disease Study 2023
Source: Lancet Glob Health. 2026 Apr 23;14(5):e734–48. doi: 10.1016/S2214-109X(26)00031-8 (PMC13122295; doi:10.1016/S2214-109X(26)00031-8)
Supplement: Supplementary appendix 1 [file mmc1.pdf]

THE LANCET
Global Health

Supplementary appendix 1

This appendix formed part of the original submission and has been peer reviewed. We post it as supplied by the authors.

Supplement to: GBD 2023 Iran Collaborators. Burden of 292 causes of death and life expectancy decomposition in Iran, 1990–2023: a systematic analysis for the Global Burden of Disease Study 2023. *Lancet Glob Health* 2026; **14:** e734–48.

Appendix 1: supplementary methods appendix to:

Burden of 292 causes of death and life expectancy decomposition in Iran, 1990–2023: a systematic analysis for the Global Burden of Disease Study 2023

This appendix provides additional detailed methods for “Burden of 292 causes of death and life expectancy decomposition in Iran, 1990–2023: a systematic analysis for the Global Burden of Disease Study 2023”

Preamble

This appendix provides further methodological detail for “Burden of 292 causes of death and life expectancy decomposition in Iran, 1990–2023: a systematic analysis for the Global Burden of Disease Study 2023.” This study complies with the Guidelines for Accurate and Transparent Health Estimates Reporting (GATHER) recommendations.[1] It includes detailed information on data in an effort to maximise transparency in our estimation processes and provide a comprehensive description of analytical steps. We intend this appendix to be a living document, to be updated with each iteration of the Global Burden of Disease Study.
Portions of this appendix have been reproduced or adapted from appendices for GBD 2021 Causes of Death Collaborators,[2] GBD 2021 Demographics Collaborators,[3] and GBD 2023 Causes of Death Collaborators[4]. References are provided for reproduced or adapted sections.

Table of Contents

Table of Contents 2

Section 1 GBD Overview 3

Section 1.1 Geographical locations and time periods of the analysis 3

Section 1.2 GBD 2023 methodological improvements 3

Section 1.3 The GBD disease and injury hierarchy 4

Section 2 GBD 2023 causes of death database 5

Section 2.1 Data sources, processing, and assessing for completeness 5

Section 2.2 Presentation of cause-specific mortality estimates 5

Section 3 Causes of death modelling methods 6

Section 3.1 CODEm 6

Section 3.1.1 Overview of methods 6

Section 3.1.2 Model pool development 6

Section 3.1.3 Data variance and uncertainty estimation 7

Section 3.1.4 Testing model pool on 15% sample 7

Section 3.1.5 Ensemble development and testing 7

Section 3.1.6 Final estimation 7

Section 3.1.7 Selection of causes for which CODEm is used 8

Section 3.1.8 Model-specific covariates 8

Section 3.2 Causes modelled outside of CODEm 8

Section 3.2.1 Overview 8

Section 3.2.2 Negative binomial models 8

Section 3.2.3 Natural history models 9

Section 3.2.4 Prevalence-based models 9

Section 3.2.5 Sub-cause proportion models 9

Section 4 COVID-19 mortality estimation 10

Section 4.1 Correction for the misclassification of COVID-19 deaths 10

Section 4.2 Estimation of COVID-19 as a cause of death 10

Section 5 Life-expectancy decomposition 12

Section 6 GBD research and reporting practices 13

Section 7 References 14

Section 1 GBD Overview

This methods appendix provides a detailed and extended description of the analytical framework, data sources, statistical models, and decomposition techniques used to estimate cause-specific mortality, COVID-19 mortality, and changes in life expectancy in Iran from 1990 to 2023. All analyses were conducted within the Global Burden of Disease, Injuries, and Risk Factors Study (GBD) 2023 framework.

Section 1.1 Geographical locations and time periods of the analysis

GBD 2023 produced estimates for each epidemiological quantity of interest for 292 causes of death by age-sex-location-year for 25 age groups from birth to 95 years and older; for males, females, and all sexes combined; in 204 countries and territories grouped into 21 regions and seven super-regions; and for every year from 1990 to 2023. This study drew on the expertise of a network of 14 410 international collaborators from more than 160 countries and territories who provide, review, and analyse the available data to generate these metrics.

In addition to national estimates, GBD 2023 produced subnational estimates for 20 countries, including Iran. For Iran, estimates were generated for all 31 provinces, enabling systematic assessment of geographic inequalities in cause-specific mortality and life expectancy. As in all GBD iterations, the entire historical time series from 1990 onwards was re-estimated to ensure internal consistency; therefore, estimates presented here supersede those from earlier GBD rounds.

Section 1.2 GBD 2023 methodological improvements

GBD 2023 produced updated estimates of health loss around the world using the best available data. For each GBD round, newly available data and updated methods are used to update the full time series of estimates from 1990 to the latest year of analysis. The methods used to generate estimates for GBD 2023 closely followed those for GBD 2021. These methods have been extensively peer reviewed over previous rounds of GBD. Here, we provide an overview of the methods with an emphasis on the main methodological changes since GBD 2021.

Methodological improvements for cause-of-death estimates in the current round of estimation focused on several key areas. First, a method for the identification and correction of causes displaying excess mortality spikes due to misclassified COVID-19 deaths was applied to all vital registration data between the years of 2020 and 2023. Second, we added new country-years of vital registration data on cause of death, country-years of surveillance data, country-years of verbal autopsy data, and country-years of other data types. Third, all CODEms were fitted to mortality rates rather than cause fractions. Fourth, we updated the modelling framework for COVID-19 to incorporate pandemic-era vital registration data and preliminary vital registration reporting.

Section 1.3 The GBD disease and injury hierarchy

GBD classifies diseases and injuries into a hierarchy with four Levels that include both fatal and non-fatal causes. Level 1 causes include three broad aggregate categories (communicable, maternal, neonatal, and nutritional [CMNN] diseases; non-communicable diseases [NCDs]; and injuries); Level 2 disaggregates those categories into 22 clusters of causes, which are further disaggregated into Level 3 and Level 4 causes. At the most detailed Level, 292 fatal causes are estimated. For GBD 2023, five causes of death were estimated for the first time: ulcerative colitis; Crohn's disease; thyroid disease; other endocrine, metabolic, blood, and immune disease; and electrocution.

Section 2 GBD 2023 causes of death database

Section 2.1 Data sources, processing, and assessing for completeness

The GBD 2023 cause-of-death database included data sources identified in previous rounds of estimation in addition to 11 474 new sources, for a total of 55 761 data sources. These sources can be accessed through the Global Health Data Exchange (GHDx) website. Multiple data types were included to capture the widest array of information, including vital registration data for all 292 causes, as well as verbal autopsy, survey, census, surveillance, cancer registry, and police record data; open-source databases; and minimally invasive tissue sampling. All raw mortality data underwent a sequence of standardised processing steps. To standardise these data so that they could be compared by cause, age, sex, location, and time, a set of data processing corrections were applied. First, deaths with insufficient or missing age and sex detail underwent a process of distribution via age and sex splitting algorithms based on empirical age–sex patterns observed in high-quality data. In addition, garbage codes, which are non-specific, implausible, or intermediate rather than underlying cause-of-death codes from the ICD, were redistributed to appropriate targets to assign the underlying cause of death using cause- and age-specific redistribution algorithms developed for GBD.[5] Data sources with more than 50% of all deaths assigned to major garbage codes (class 1 or class 2 garbage codes) in any location-year were excluded to mitigate the potential for bias from these sources.

Assessing data completeness illustrates the coverage from a data source on overall mortality for the country. Vital registration and verbal autopsy data completeness—a source-specific estimate of the percentage of total cause-specific deaths that are reported in a given location and year—was assessed by location-year, and sources with less than 50% completeness or with excessive proportions of major garbage codes were excluded. The estimated all-cause mortality for each age-sex-location-year was then multiplied by the cause fraction for the corresponding age-sex-location-year to adjust all included sources to 100% completeness. GBD assesses the quality of all vital registration and verbal autopsy data using a star ranking system of one to five stars, based on the percentage of completeness and percentage of garbage coding.

Section 2.2 Presentation of cause-specific mortality estimates

Cause-specific mortality estimates for GBD 2023 are given in death counts and age-standardised rates per 100 000 population, calculated using the GBD standard population structure.[6] For changes over time, we present percentage changes over the period 1990–2023, and annualised rates of change as the difference in the natural log of the values at the start and end of the time interval divided by the number of years in the interval. 95% uncertainty intervals (UIs) for all metrics are computed using the 2·5th and 97·5th percentiles from a 250-draw distribution for each metric. To reduce computing power and time, the number of computations per process was scaled back from 500 in GBD 2021 to 250 in GBD 2023, as simulation testing revealed that final estimates and their uncertainty were not affected by this reduction.

Section 3 Causes of death modelling methods

Section 3.1 CODEm

Section 3.1.1 Overview of methods

Cause of death ensemble modelling (CODEm) is the framework used to model most cause-specific death rates in the GBD.[7] It relies on four key components: First, all available data are identified and gathered to be used in the modelling process. Although the data may vary in quality, they all contain some signal of the true epidemiological process. Second, a diverse set of plausible models are developed to capture well-documented associations in the estimates. Using a wide variety of individual models to create an ensemble predictive model has been shown to outperform techniques using only a single model both in CoD estimation[7] and in more general prediction applications. Third, the out-of-sample predictive validity is assessed for all individual models, which are then ranked for use in the ensemble modelling stage. Finally, differently weighted combinations of individual models are evaluated to select the ensemble model with the highest out-of-sample predictive validity.

For some causes (eg, lower respiratory infections), evidence exists that the relationship between covariates and death rates might differ between children and adults. Separate models are therefore run for different age ranges, when applicable.

In addition to CoD modelling, we also estimate fatal discontinuities. Fatal discontinuities are events that are stochastic in nature, that cannot be modelled because they do not have a predictable time trend. The fatal discontinuities by cause are aggregated by age and sex and added to the estimated number of deaths in CoD modelling for those causes during CoDCorrect.

Section 3.1.2 Model pool development

Because many factors may co-vary with any given CoD, a range of plausible statistical models are developed for each cause. In the CODEm framework, four families of statistical models are used: linear mixed effects regression (LMER) models of the natural log of the cause-specific death rate, LMER models of the logit of the cause fraction, spatiotemporal Gaussian process regression (ST-GPR) models of the natural logarithm of the cause-specific death rate, and ST-GPR models of the logit of the cause fraction.[7]

For each family of models, all plausible relationships between covariates and the response variable are identified. Because all possible combinations of selected covariates are considered for each family of models, multi-collinearity between covariates may produce implausible signs on coefficients or unstable coefficients. Each combination is therefore tested for statistical significance (covariate coefficients must have a coefficient with p-value $<0{\cdot}05$) and plausibility (the coefficients must have the directions expected on the basis of the literature). Only covariate combinations meeting these criteria are retained. This selection process is run for both cause fractions and death rates, then ST-GPR and LMER-only models are created for each set of covariates.

Section 3.1.3 Data variance and uncertainty estimation

The families of models that go through ST-GPR described above incorporate information about data variance. The main inputs for a Gaussian process regression (GPR) are a mean function, a covariance function, and data variance for each datapoint. For GBD 2019, we updated this calculation to incorporate garbage code redistribution uncertainty.

Three components of data variance are now used in CODEm: sampling variance, non-sampling variance, and garbage code redistribution variance. The computation of sampling variance and non-sampling variance has not changed since previous iterations of the GBD and is also described in Foreman et al.[7] Garbage code redistribution variance is computed in the CoD database process described in this appendix. Since variance is additive, we calculate total data variance as the sum of sampling variance, non-sampling variance, and redistribution variance. Increased data variance in GPR results in the GPR draws not following the datapoint as closely.

Section 3.1.4 Testing model pool on 15% sample

The performance of all models (individual and ensemble) is evaluated by means of out-of-sample predictive validity tests. 30% of the data are randomly excluded from the initial model fits. These individual model fits are evaluated and ranked by using half of the excluded data (15% of the total), then used to construct the ensembles on the basis of their performance. Data are held out from the analysis on the basis of the cause-specific missingness patterns for ages and years across locations. Out-of-sample predictive validity testing is repeated 20 times for each model, which has been shown to produce stable results.[7] These performance tests include the root mean square error (RMSE) for the log of the cause-specific death rate, the direction of the predicted versus actual trend in the data, and the coverage of the predicted 95% UI.

Section 3.1.5 Ensemble development and testing

The component models are weighted on the basis of their predictive validity rank to determine their contribution to the ensemble estimate. The relative weights are determined both by the model ranks and by a parameter ψ, whose value determines how quickly the weights taper off as rank decreases. A set of ensemble models is then created by using the weights constructed from the combinations of ranks and ψ values. These ensembles are tested by using the predictive validity metrics on the remaining 15% of the data, and the ensemble with the best performance in out-of-sample trend and RMSE is chosen as the final model.

Section 3.1.6 Final estimation

Once a weighting scheme has been chosen, 250 draws are created for the final ensemble, and the number of draws contributed by each model is proportional to its weight. The mean of the draws is used as the final estimate for the CODEm process, and a 95% UI is created from the 0·025 and 0·975 quantiles of the draws. The validity of the UI can be checked via its coverage of the out-of-sample data; ideally, the 95% UI would capture 95% of these data. Higher coverage suggests that the UIs are too large, and lower coverage suggests overfitting. To reduce computing power and time, we reduced the number of draws (or computations) per process to 250, from 500 in

GBD 2021. Based on simulation testing, we determined that a change in the number of draws did not impact final mean estimates, nor lead to inappropriately narrow uncertainty estimates.

Section 3.1.7 Selection of causes for which CODEm is used

CODEm is used to model 235 causes. However, it is unsuitable for use in modelling certain causes, including those with very low death counts, those where cause-specific death record availability is inadequate, or those for which there are marked biases or variability for CoD certification over time that cannot be fully accounted for with the current garbage code redistribution algorithms.

Section 3.1.8 Model-specific covariates

Modellers select covariates to be used in CODEm, but those covariates may not be significant or in the direction specified during the covariate selection step of CODEm and will therefore not be used in the model. Covariates may be selected by CODEm but only exist in sub-models that perform poorly and may end up with zero draws included in the final ensemble. Finally, all other covariates are listed with the number of draws in the final ensemble from sub-models that had the covariate.

Section 3.2 Causes modelled outside of CODEm

Section 3.2.1 Overview

A number of causes required alternative modelling strategies to those used for CODEm because they were not compatible with CODEm estimation infrastructure and processes. Such unsuitability included having very low death counts; inadequate availability of cause-specific death records; and marked biases or variability for CoD certification over time that could not be fully accounted for with current garbage code redistribution algorithms. The inclusion of these causes in CODEm often renders its out-of-sample predictive validity testing unstable, but the validity of this type of testing is a key advantage of using CODEm for CoD estimation. Alternately, CODEm simply fails to generate plausible mortality rates in the absence of enough VR or VA data when these causes are included.

For GBD 2023, we used alternative modelling approaches for these causes, including negative binomial models, natural history models, prevalence-based models, and sub-cause proportion models.

Section 3.2.2 Negative binomial models

For 78 rare causes of death, too few observed deaths were included in the CoD database to produce stable estimates. For these causes, we estimated mortality by fitting count models - either negative binomial or Poisson - to available CoD data.

Section 3.2.3 Natural history models

For some causes for which CoD data may be systematically biased either owing to misclassification or because the disease exists in focal communities without VR or VA studies, we have developed natural history models. In natural history models, incidence and case-fatality ratios are modelled separately and then combined to produce estimates of cause-specific mortality.

Section 3.2.4 Prevalence-based models

The modelling strategies for atrial fibrillation and flutter are distinct from those used for other causes modelled as natural history models. These models use prevalence estimates and excess mortality rates (EMR) generated through DisMod-MR 2.1 rather than incidence and case-fatality rates. This approach allows us to adjust estimates to more accurately reflect the number of deaths for which atrial fibrillation was the true underlying cause of death.

Section 3.2.5 Sub-cause proportion models

For certain sub-causes for which accurate diagnoses are known to be very difficult, we first modelled the parent cause in the GBD hierarchy with CODEm and then allocated deaths to specific causes by using proportions of the parent cause for each age-sex-location-year for each sub-cause. For these causes, we identified no significant predictors in negative binomial regressions. This approach was taken because the available data on these specific causes may come from sources other than VR, such as end-stage renal disease registries, or may come from too few places to model the death rates directly.

Section 4 COVID-19 mortality estimation

Section 4.1 Correction for the misclassification of COVID-19 deaths

GBD 2023 received new country-years of vital registration data from 2020, 2021, and 2022. During these years, there is evidence that deaths due to COVID-19 were misclassified as other causes of death.[8,9] Relative to smooth pre-pandemic mortality trends, these misclassified COVID-19 deaths contributed to mortality spikes in the other causes of death. To systematically identify these spikes in other causes, we developed a Support Vector Machine, a machine-learning algorithm for identifying deviations from the established time trends in the years 2020–22. After we identified causes with mortality spikes during the COVID-19 pandemic years, we ascertained, for each cause of interest, whether the spike was a result of COVID-19 misclassification or a true increase in mortality. To do this, we evaluated the correlation between the rate of excess mortality in the cause of interest and the observed mortality rate of COVID-19. Mortality spikes identified to contain COVID-19 misclassification were then considered eligible for correction.

When a mortality spike had been identified as being eligible for correction, we calculated the portion of excess mortality attributable to misclassified COVID-19. We first created an estimate of expected deaths absent of any pandemic effects using the mean of two counterfactual estimates: one calculated by a linear regression of the 5 years before the start of the pandemic (2015–19), and another calculated using a global relative rate of non-COVID-19 deaths, adapted from a previously published method used in the correction of misclassified HIV.[10] Total excess mortality could then be estimated by subtracting the expected death count total from the observed death count total. Finally, total excess mortality was then scaled according to the level of correlation between COVID-19 rate and excess mortality rate to calculate the amount of excess attributable to COVID-19. The total excess attributable to COVID-19 was then subtracted from the cause of interest and reassigned to COVID-19.

Section 4.2 Estimation of COVID-19 as a cause of death

For modelling COVID-19, we supplemented the corrected vital registration data described above with two other sources of data: 9 country-years of provisional vital registration data and 342 country-years of surveillance data that were reported during the pandemic (to 2022). We developed an analysis method using OneMod, a modelling tool that combines robust feature selection, correlated time-series splines, and covariate effect sizes across age groups, in addition to kernel regression for residual smoothing. It included the following candidate covariates: total COVID-19 infections and variant prevalence; COVID-19 vaccinations;[11] COVID-19 infection detection rate;[11] Healthcare Access and Quality Index;[12] and prevalence of risk factors and comorbidities including obesity, smoking, cancer, cardiovascular disease, chronic kidney disease, chronic obstructive pulmonary disease, and diabetes.[13,14] In the first stage of this model pipeline, we used only the corrected vital registration data to estimate age patterns and sex ratios, which were then used to split the provisional vital registration by age and sex, and split the surveillance data that did not contain detailed age and sex information into the 25 granular GBD age groups. We then ran the models using the entire dataset, setting the infection detection rate to

100% for the corrected vital registration data. After fitting these models, we made predictions assuming that the infection detection rate was 100% in all locations.

Section 5 Life-expectancy decomposition

The objective of life-expectancy decomposition is to analyse the difference in life expectancy by age and location, quantifying contributions from specific causes. We examined temporal trends in causes over continuous time periods across different locations. We aimed to identify the effect of causes of death on life expectancy by using three main decomposition steps. For this study, we investigated the top-20 Level 2 and Level 3 GBD causes contributing to change in life expectancy. The remaining causes were then combined as “other communicable and maternal disorders” or “other NCDs”.

The first step involved decomposing the difference in life expectancy by age. We calculated age-specific contributions to understand the variation in life expectancy across different age groups. In the second step, each age-specific contribution was further decomposed into cause-age specific contributions. This analysis allowed for the identification of the specific causes of death that contributed to the differences in life expectancy within each age group.

Finally, we aggregated the cause-age specific contributions across age groups to produce cause-specific contributions to the overall difference in life expectancy. This aggregation provided a comprehensive understanding of how different causes of death contributed to the observed variations in life expectancy. By applying this decomposition approach, we gain insights into the relative effect of different causes of death on changes in life expectancy by age and location.[2]

Section 6 GBD research and reporting practices

This study used de-identified data and was approved by the University of Washington Institutional Review Board (study number 9060). GBD 2023 complies with the Guidelines for Accurate and Transparent Health Estimates Reporting (GATHER) statement. Software packages used in the cause of death analysis for GBD 2023 were Python version 3.10.4, Stata version 13.1, and R version 4.4.0. Statistical code used for GBD estimation is publicly available online at the GHDx website.

Section 7 References

1 Stevens GA, Alkema L, Black RE, et al. Guidelines for Accurate and Transparent Health Estimates Reporting: the GATHER statement. Lancet 2016; 388: e19–23.

2 GBD 2021 Causes of Death Collaborators. Global burden of 288 causes of death and life expectancy decomposition in 204 countries and territories and 811 subnational locations, 1990-2021: a systematic analysis for the Global Burden of Disease Study 2021. Lancet 2024; 403: 2100–32.

3 GBD 2021 Demographics Collaborators. Global age-sex-specific mortality, life expectancy, and population estimates in 204 countries and territories and 811 subnational locations, 1950-2021, and the impact of the COVID-19 pandemic: a comprehensive demographic analysis for the Global Burden of Disease Study 2021. Lancet 2024; 403: 1989–2056.

4 GBD 2023 Causes of Death Collaborators. Global burden of 292 causes of death in 204 countries and territories and 660 subnational locations, 1990-2023: a systematic analysis for the Global Burden of Disease Study 2023. Lancet 2025; 406: 1811–72.

5 Johnson SC, Cunningham M, Dippenaar IN, et al. Public health utility of cause of death data: applying empirical algorithms to improve data quality. BMC Med Inform Decis Mak 2021; 21: 175.

6 GBD 2023 Demographics Collaborators. Global age-sex-specific all-cause mortality and life expectancy estimates for 204 countries and territories and 660 subnational locations, 1950–2023: a demographic analysis for the Global Burden of Disease Study 2023. Lancet 2025; published online Oct 12. https://doi.org/10.1016/S0140- 6736(25)01330-3.

7 Foreman KJ, Lozano R, Lopez AD, Murray CJ. Modeling causes of death: an integrated approach using CODEm. Popul Health Metr 2012; 10: 1.

8 França EB, Ishitani LH, de Abreu DMX, et al. Measuring misclassification of COVID-19 as garbage codes: results of investigating 1365 deaths and implications for vital statistics in Brazil. PLOS Glob Public Health 2022; 2: e0000199.

9 US Centers for Disease Control and Prevention. Excess deaths associated with COVID-19. Sept 28, 2023. https://www.cdc.gov/ nchs/nvss/vsrr/covid19/excess_deaths.htm (accessed Jan 13, 2025).

10 Birnbaum JK, Murray CJ, Lozano R. Exposing misclassified HIV/ AIDS deaths in South Africa. Bull World Health Organ 2011; 89: 278–85.

11 COVID-19 Forecasting Team. Forecasting the trajectory of the COVID-19 pandemic into 2023 under plausible variant and intervention scenarios: a global modelling study. medRxiv 2023; published online March 8. https://doi.org/10.1101/ 2023.03.07.23286952 (preprint).

12 GBD 2019 Healthcare Access and Quality Collaborators. Assessing performance of the Healthcare Access and Quality Index, overall and by select age groups, for 204 countries and territories, 1990–2019: a systematic analysis from the Global Burden of Disease Study 2019. Lancet Glob Health 2022; 10: e1715–43.

13 GBD 2021 Diseases and Injuries Collaborators. Global incidence, prevalence, years lived with disability (YLDs), disability-adjusted life-years (DALYs), and healthy life expectancy (HALE) for 371 diseases and injuries in 204 countries and territories and 811 subnational locations, 1990–2021: a systematic analysis for the Global Burden of Disease Study 2021. Lancet 2024; 403: 2133–61.

14 Institute for Health Metrics and Evaluation. GBD Results. https:// vizhub.healthdata.org/gbd-results (accessed July 14, 2025).
