# Supplementary material for: Burden of 292 causes of death and life expectancy decomposition in Iran, 1990–2023: a systematic analysis for the Global Burden of Disease Study 2023
Source: Lancet Glob Health. 2026 Apr 23;14(5):e734–48. doi: 10.1016/S2214-109X(26)00031-8 (PMC13122295; doi:10.1016/S2214-109X(26)00031-8)
Supplement: Supplementary appendix 2 [file mmc2.pdf]

THE LANCET
Global Health

Supplementary appendix 2

This appendix formed part of the original submission and has been peer reviewed. We post it as supplied by the authors.

Supplement to: GBD 2023 Iran Collaborators. Burden of 292 causes of death and life expectancy decomposition in Iran, 1990–2023: a systematic analysis for the Global Burden of Disease Study 2023. *Lancet Glob Health* 2026; **14:** e734–48.

Appendix 2: supplementary results appendix to:

Burden of 292 causes of death and life expectancy decomposition in Iran, 1990–2023: a systematic analysis for the Global Burden of Disease Study 2023

This appendix provides additional figures and tables containing more detailed results for "Burden of 292 causes of death and life expectancy decomposition in Iran, 1990–2023: a systematic analysis for the Global Burden of Disease Study 2023"

Supplementary figures and tables

Figures

Figure S1. Age-standardised mortality rate in Iran from 1990 to 2023.

Figure S2. Life expectancy at birth across provinces of Iran for both sexes combined

Figure S3. Life expectancy decomposition for all causes across Iran and the 31 provinces in both sexes from 1995 to 2023

Figure S4. Life expectancy decomposition for all causes across Iran and the 31 provinces in females from 1995 to 2023

Figure S5. Life expectancy decomposition for all causes across Iran and the 31 provinces in males from 1995 to 2023

Figure S6. Age-standardised mortality rates per 100 000 for communicable, maternal, neonatal, and nutritional (CMNN) diseases in Iran and 31 provinces in both sexes in 2023.

Figure S7. Age-standardised mortality rates per 100 000 for communicable, maternal, neonatal, and nutritional (CMNN) diseases in Iran and 31 provinces in females in 2023.

Figure S8. Age-standardised mortality rates per 100 000 for communicable, maternal, neonatal, and nutritional (CMNN) diseases in Iran and 31 provinces in males in 2023.

Figure S9. Age-standardised mortality rates per 100 000 for cardiovascular diseases in Iran and 31 provinces in both sexes in 2023.

Figure S10. Age-standardised mortality rates per 100 000 for cardiovascular diseases in Iran and 31 provinces in females in 2023.

Figure S11. Age-standardised mortality rates per 100 000 for cardiovascular diseases in Iran and 31 provinces in males in 2023.

Figure S12. Age-standardised mortality rates per 100 000 for top 10 neoplasms in Iran and 31 provinces in both sexes in 2023.

Figure S13. Age-standardised mortality rates per 100 000 for top 10 neoplasms in Iran and 31 provinces in females in 2023.

Figure S14. Age-standardised mortality rates per 100 000 for top 10 neoplasms in Iran and 31 provinces in males in 2023.

Figure S15. Age-standardised mortality rates per 100 000 for top 5 non-communicable diseases in Iran and 31 provinces in both sexes in 2023

Figure S16. Age-standardised mortality rates per 100 000 for top 5 non-communicable diseases in Iran and 31 provinces in females in 2023

Figure S17. Age-standardised mortality rates per 100 000 for top 5 non-communicable diseases in Iran and 31 provinces in males in 2023

Figure S18. Age-standardised mortality rates per 100 000 for top 10 injuries in Iran and 31 provinces in both sexes in 2023.

Figure S19. Age-standardised mortality rates per 100 000 for top 10 injuries in Iran and 31 provinces in females in 2023.

Figure S20. Age-standardised mortality rates per 100 000 for top 10 injuries in Iran and 31 provinces in males in 2023.

Tables

Table S1. All-cause mortality counts and age-standardised mortality rates per 100 000 across 31 provinces in both sexes combined in 2019, 2020, and 2023

Table S2. All-cause mortality counts and age-standardised mortality rates per 100 000 across 31 provinces in females in 2019, 2020, and 2023

Table S3. All-cause mortality counts and age-standardised mortality rates per 100 000 across 31 provinces in males in 2019, 2020, and 2023

Table S4. Age-standardised mortality rates for top 12 causes of death at national level in Iran in 2019, 2020, 2021, 2022, and 2023 in females.

Table S5. Age-standardised mortality rates for top 12 causes of death at national level in Iran in 2019, 2020, 2021, 2022, and 2023 in Males.

Table S6. Counts of deaths for top 12 causes of death at national level in Iran in 2019, 2020, 2021, 2022, and 2023 in both sexes.

Table S7. Counts of deaths for top 12 causes of death at national level in Iran in 2019, 2020, 2021, 2022, and 2023 in females.

Table S8. Counts of deaths for top 12 causes of death at national level in Iran in 2019, 2020, 2021, 2022, and 2023 in males.

Table S9. Life expectancy at birth across 31 provinces in both sexes combined in 1990, 2019, 2020, 2021, 2022, and 2023

Table S10. Life expectancy at birth across 31 provinces in females in 1990, 2019, 2020, 2021, 2022, and 2023

Table S11. Life expectancy at birth across 31 provinces in males in 1990, 2019, 2020, 2021, 2022, and 2023

Table S12. COVID-19 mortality counts across 31 provinces in both sexes combined in 2020, 2021, 2022, and 2023

Table S13. COVID-19 mortality counts across 31 provinces in females in 2020, 2021, 2022, and 2023

Table S14. COVID-19 mortality counts across 31 provinces in males in 2020, 2021, 2022, and 2023

Table S15. The age-standardised rates of COVID-19 in 2020, 2021, 2022, and 2023 and its contribution to change in life expectancy in females

Table S16. The age-standardised rates of COVID-19 in 2020, 2021, 2022, and 2023 and its contribution to change in life expectancy in males

Table S17. Contribution of communicable, maternal, neonatal, and nutritional (CMNN) diseases to change in life expectancy from 1990 to 2023 in both sexes combined

Table S18. Contribution of communicable, maternal, neonatal, and nutritional (CMNN) diseases to change in life expectancy from 1990 to 2023 in females

Table S19. Contribution of communicable, maternal, neonatal, and nutritional (CMNN) diseases to change in life expectancy from 1990 to 2023 in males

Table S20. Contribution of cardiovascular diseases to change in life expectancy from 1990 to 2023 in both sexes combined

Table S21. Contribution of cardiovascular diseases to change in life expectancy from 1990 to 2023 in females

Table S22. Contribution of cardiovascular diseases to change in life expectancy from 1990 to 2023 in males

Table S23. Contribution of neoplasms to change in life expectancy from 1990 to 2023 in both sexes combined

Table S24. Contribution of neoplasms to change in life expectancy from 1990 to 2023 in females

Table S25. Contribution of neoplasms to change in life expectancy from 1990 to 2023 in males

Table S26. Contribution of major non-communicable diseases to change in life expectancy from 1990 to 2023 in both sexes combined

Table S27. Contribution of major non-communicable diseases to change in life expectancy from 1990 to 2023 in females

Table S28. Contribution of major non-communicable diseases to change in life expectancy from 1990 to 2023 in males

Table S29. Contribution of injuries to change in life expectancy from 1990 to 2023 in both sexes combined

Table S30. Contribution of injuries to change in life expectancy from 1990 to 2023 in females

Table S31. Contribution of injuries to change in life expectancy from 1990 to 2023 in males

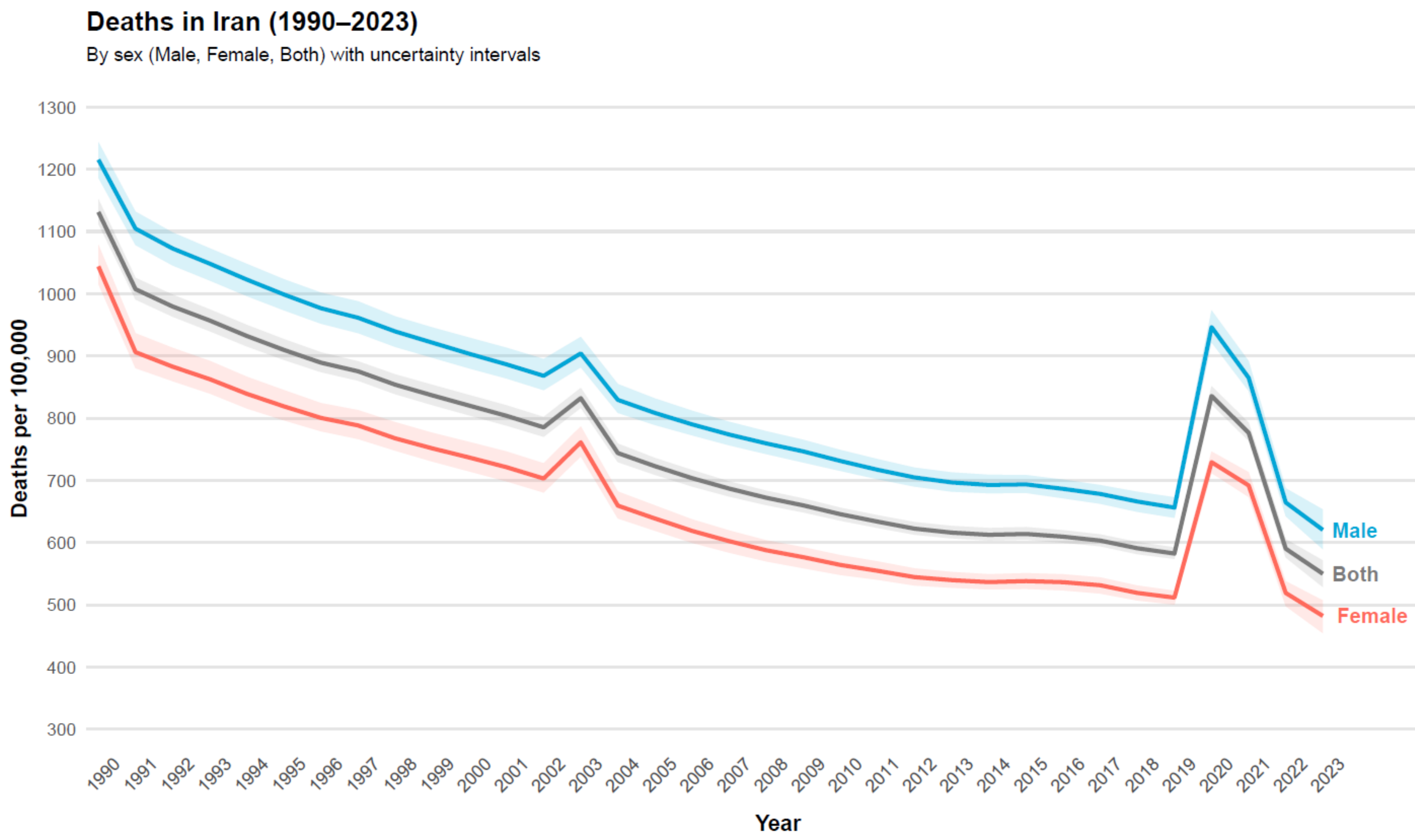

Figure S1. Age-standardised mortality rate in Iran from 1990 to 2023.

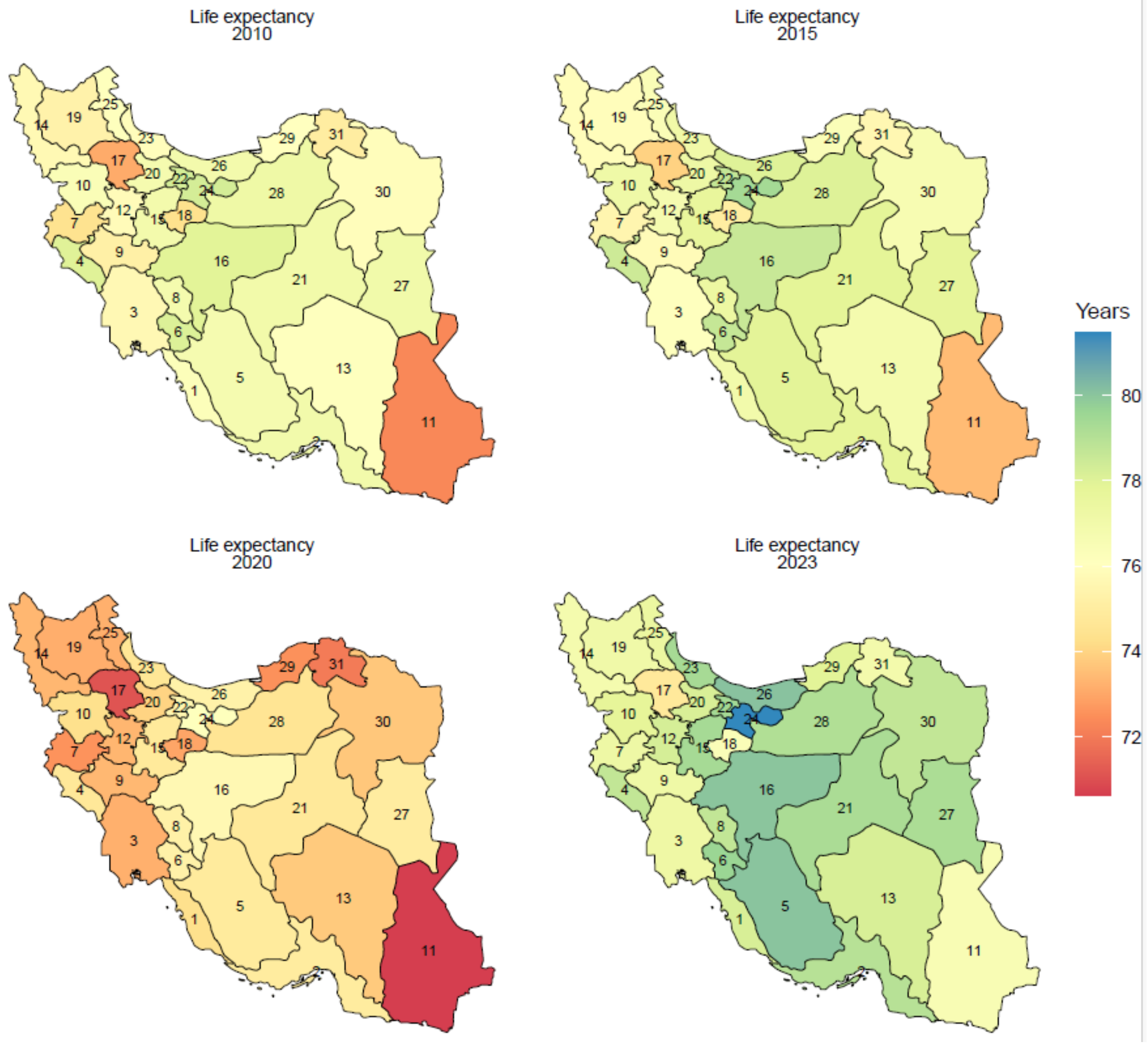

Figure S2. Life expectancy at birth across provinces of Iran for both sexes combined

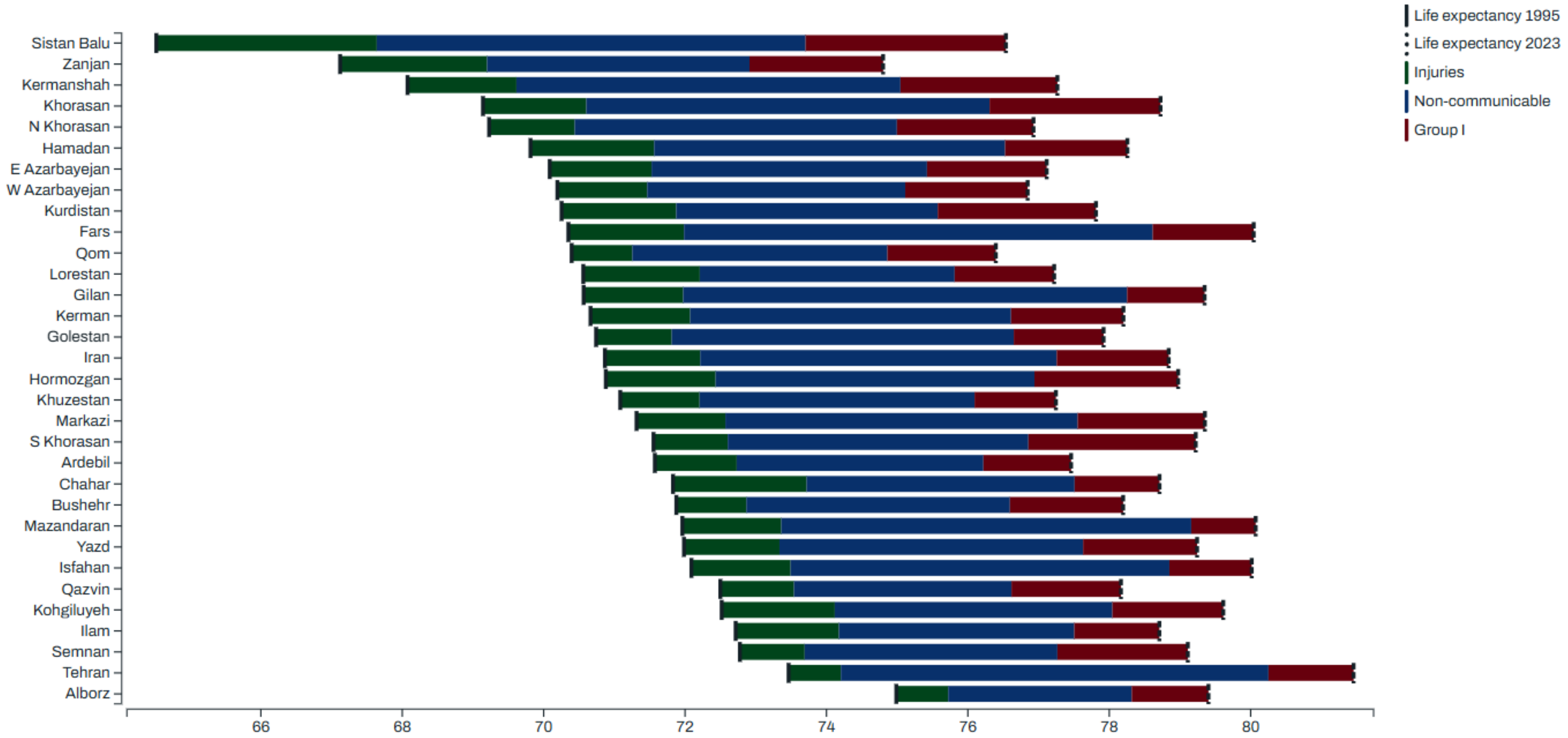

Figure S3. Life expectancy decomposition for all causes across Iran and the 31 provinces in both sexes from 1995 to 2023

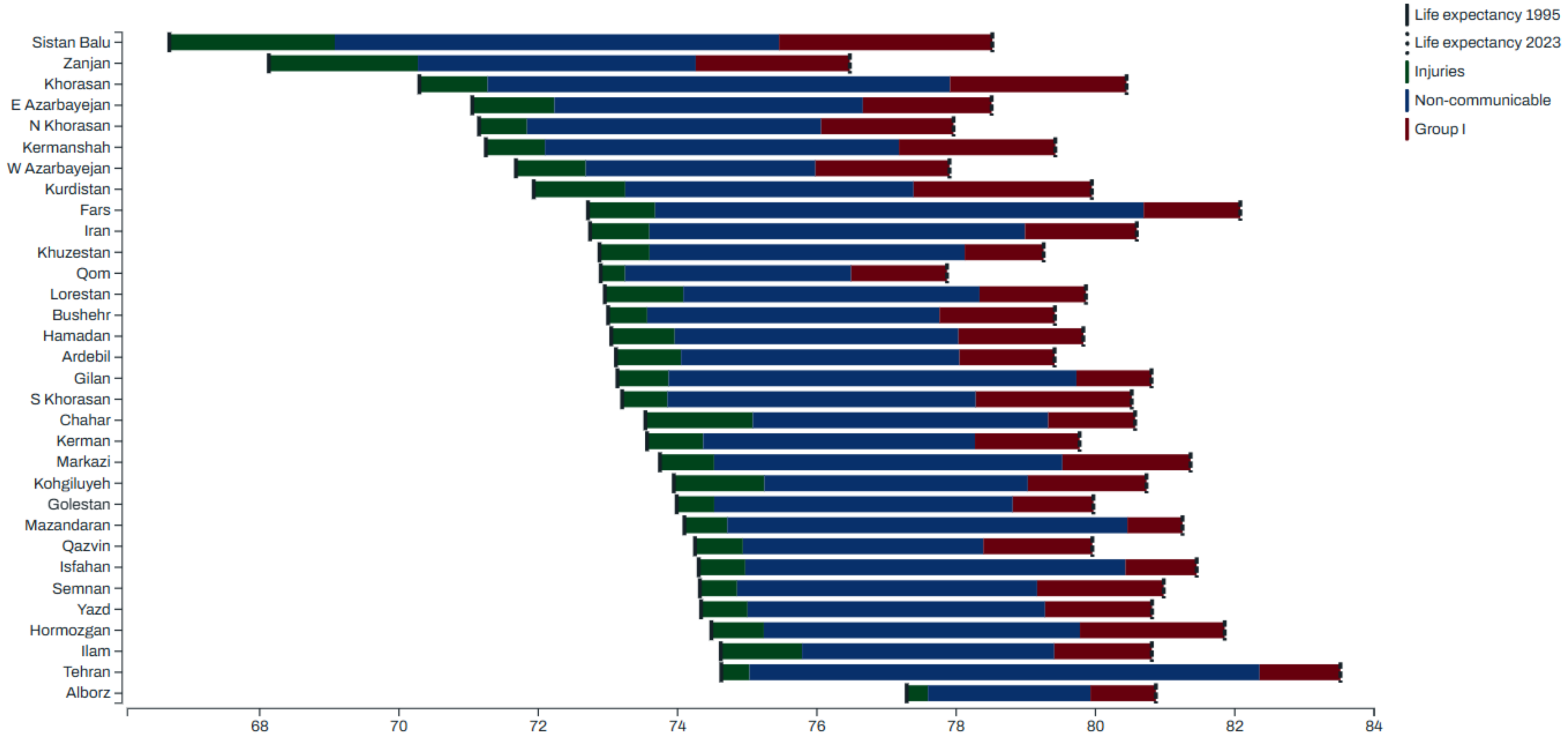

Figure S4. Life expectancy decomposition for all causes across Iran and the 31 provinces in females from 1995 to 2023

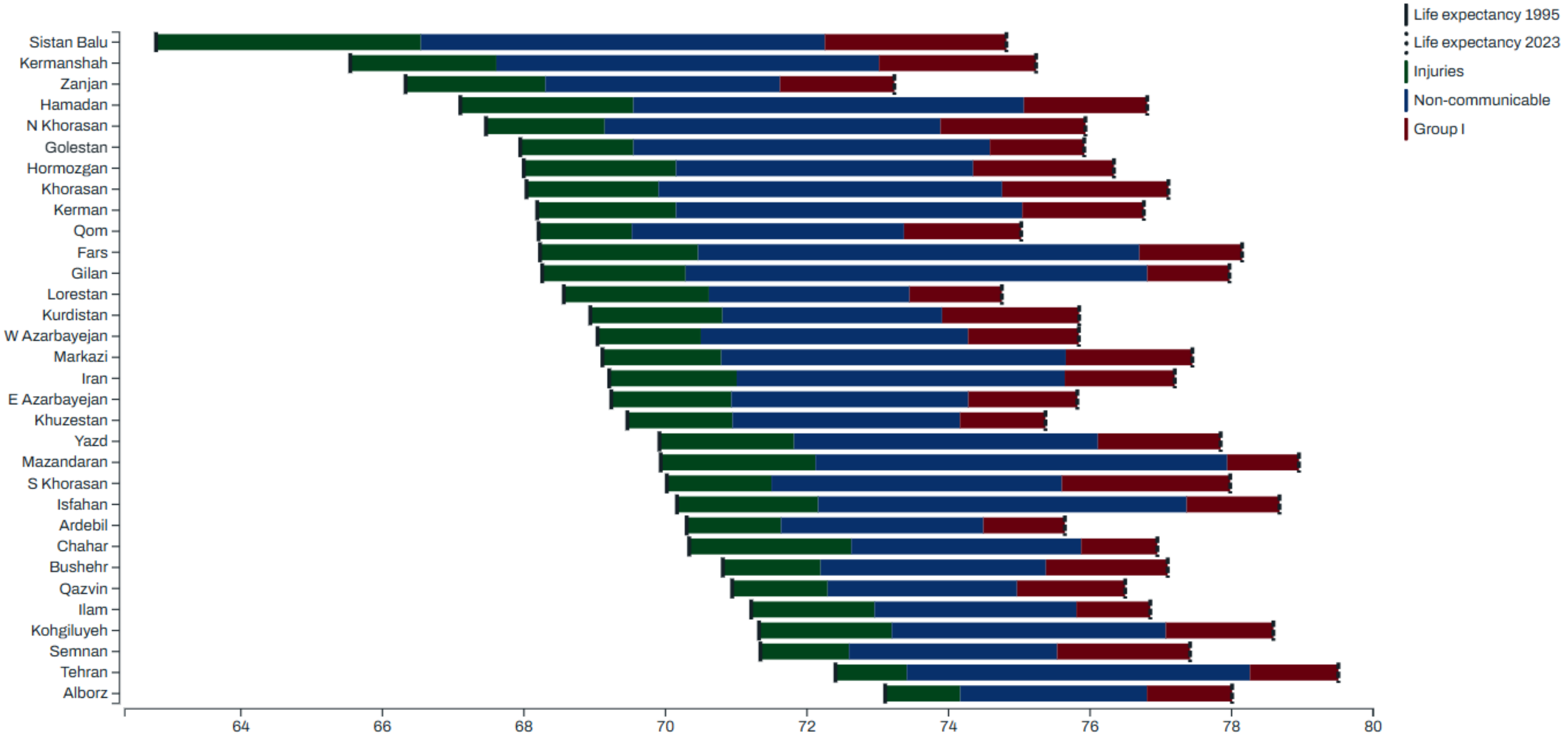

Figure S5. Life expectancy decomposition for all causes across Iran and the 31 provinces in males from 1995 to 2023

Location	Lower respiratory infections	Congenital birth defects	Neonatal disorders	Diarrheal diseases	Tuberculosis	Meningitis	Hemoglobinopathies and hemolytic anemias	Protein energy malnutrition
Iran (Islamic Republic of)	18.69	9.01	8.93	1.25	1.02	0.76	0.60	0.46
Alborz	24.48	6.20	6.35	1.28	0.87	0.60	0.27	0.30
Ardebil	8.60	9.54	6.58	0.98	0.68	0.52	0.34	0.15
Bushehr	18.96	10.22	10.49	1.49	0.86	0.67	0.84	2.05
Chahar Mahaal and Bakhtiari	9.06	9.36	6.73	0.53	0.20	0.34	0.64	0.22
East Azarbayejan	22.20	9.63	6.53	1.31	0.78	0.75	0.22	1.38
Fars	17.38	11.00	11.07	0.93	0.46	0.70	0.72	0.23
Gilan	13.14	6.81	6.08	1.31	0.77	0.58	0.62	0.20
Golestan	14.77	8.16	8.11	1.33	2.40	0.94	0.75	0.35
Hamadan	14.53	9.38	11.24	1.18	0.54	1.19	0.28	0.13
Hormozgan	16.16	9.78	11.85	2.05	1.09	0.77	1.30	0.18
Ilam	11.90	8.68	8.33	1.19	0.54	0.52	0.59	0.51
Isfahan	17.11	9.30	7.74	0.98	0.45	0.60	0.34	0.48
Kerman	24.76	9.47	11.42	1.84	1.00	0.81	0.72	0.29
Kermanshah	17.69	11.51	13.73	1.27	0.97	0.70	0.46	0.76
Khorasan-e-Razavi	25.74	9.91	9.94	1.53	1.60	0.76	0.31	0.84
Khuzestan	19.52	10.18	8.86	1.15	1.38	0.83	0.94	0.48
Kohgiluyeh and Boyer-Ahmad	10.69	9.19	8.42	1.04	0.45	1.34	1.06	0.17
Kurdistan	13.05	9.06	11.83	1.13	0.74	0.77	0.20	0.49
Lorestan	19.94	6.80	6.59	1.24	0.98	0.77	0.78	0.70
Markazi	20.07	9.03	10.92	1.02	0.88	0.66	0.24	0.15
Mazandaran	13.42	7.17	5.70	0.95	0.57	0.53	0.67	0.38
North Khorasan	30.75	8.60	8.89	1.92	0.95	0.89	0.21	0.28
Qazvin	16.39	9.46	8.42	1.22	0.52	0.69	0.28	0.21
Qom	36.43	8.90	7.27	1.66	2.16	0.83	0.34	0.78
Semnan	21.45	11.09	10.71	0.91	0.82	0.47	0.37	0.39
Sistan and Baluchistan	24.73	8.93	11.08	3.03	4.14	2.19	1.72	0.30
South Khorasan	31.37	8.57	11.20	1.40	1.52	0.70	0.17	0.36
Tehran	15.82	7.53	6.98	0.80	1.08	0.61	0.67	0.32
West Azarbayejan	18.36	9.26	8.07	1.42	0.75	0.64	0.26	0.39
Yazd	23.35	10.87	9.32	1.23	1.02	0.55	0.21	0.66
Zanjan	28.86	9.46	9.34	1.81	0.87	1.19	0.66	0.63

Figure S6. Age-standardised mortality rates per 100 000 for communicable, maternal, neonatal, and nutritional (CMNN) diseases in Iran and 31 provinces in both sexes in 2023.

Red and orange colours show higher rates. Yellow and green colours show lower rates.

Location	Lower respiratory infections	Congenital birth defects	Neonatal disorders	Diarrheal diseases	Tuberculosis	Meningitis	Hemoglobinopathies and hemolytic anemias	Protein energy malnutrition
Iran (Islamic Republic of)	16.75	8.55	7.82	1.28	0.94	0.69	0.61	0.49
Alborz	22.86	5.56	5.47	1.49	0.90	0.53	0.28	0.40
Ardebil	6.98	10.40	5.76	0.88	0.65	0.55	0.43	0.16
Bushehr	16.81	9.91	9.43	1.49	0.75	0.63	0.95	2.14
Chahar Mahaal and Bakhtiari	7.36	9.74	5.92	0.47	0.19	0.31	0.73	0.15
East Azarbayejan	20.84	9.68	5.98	1.33	0.78	0.68	0.25	1.56
Fars	14.19	10.09	9.59	0.87	0.39	0.62	0.72	0.25
Gilan	12.09	6.34	5.33	1.35	0.57	0.53	0.65	0.17
Golestan	12.39	7.50	6.83	1.29	2.00	0.86	0.80	0.24
Hamadan	12.86	9.06	9.97	1.14	0.49	1.04	0.23	0.08
Hormozgan	13.74	9.02	9.58	1.91	0.80	0.70	1.37	0.12
Ilam	10.57	9.04	7.14	1.14	0.48	0.37	0.58	0.33
Isfahan	15.27	8.60	6.92	0.96	0.44	0.56	0.35	0.53
Kerman	22.22	8.81	10.01	1.89	0.94	0.76	0.72	0.24
Kermanshah	15.21	11.38	12.47	1.28	0.87	0.64	0.49	0.77
Khorasan-e-Razavi	23.64	9.41	8.92	1.55	1.54	0.71	0.33	1.01
Khuzestan	16.76	9.76	7.91	1.17	1.07	0.76	0.88	0.56
Kohgiluyeh and Boyer-Ahmad	10.33	9.07	7.42	1.10	0.46	1.25	1.20	0.17
Kurdistan	12.09	9.33	10.40	1.20	0.83	0.71	0.25	0.59
Lorestan	15.66	6.99	5.37	1.10	0.78	0.67	0.81	0.82
Markazi	16.69	8.92	10.04	0.96	0.79	0.56	0.22	0.13
Mazandaran	11.65	6.44	4.88	0.95	0.45	0.44	0.63	0.41
North Khorasan	31.12	8.22	7.67	2.07	0.95	0.82	0.12	0.32
Qazvin	13.58	9.37	7.12	1.28	0.50	0.70	0.32	0.24
Qom	33.37	8.45	6.44	1.70	2.12	0.69	0.28	1.03
Semnan	18.22	10.90	9.17	0.92	0.79	0.42	0.39	0.31
Sistan and Baluchistan	21.83	8.64	9.65	3.07	4.07	2.10	2.01	0.17
South Khorasan	29.03	7.42	10.07	1.35	1.37	0.71	0.10	0.47
Tehran	15.11	6.40	5.94	0.93	1.00	0.50	0.62	0.30
West Azarbayejan	16.63	9.72	7.30	1.49	0.67	0.60	0.29	0.31
Yazd	22.07	10.27	8.33	1.26	0.94	0.48	0.23	0.64
Zanjan	24.85	10.09	8.17	1.83	0.73	1.05	0.82	1.00

Figure S7. Age-standardised mortality rates per 100 000 for communicable, maternal, neonatal, and nutritional (CMNN) diseases in Iran and 31 provinces in females in 2023.

Red and orange colours show higher rates. Yellow and green colours show lower rates.

Location	Lower respiratory infections	Neonatal disorders	Congenital birth defects	Diarrheal diseases	Tuberculosis	Meningitis	Hemoglobinopathies and hemolytic anemias	Protein energy malnutrition
Iran (Islamic Republic of)	20.79	9.96	9.45	1.22	1.11	0.84	0.59	0.43
Alborz	26.20	7.17	6.79	1.07	0.84	0.67	0.27	0.20
Ardebil	10.80	7.32	8.75	1.13	0.73	0.49	0.25	0.15
Bushehr	21.40	11.47	10.51	1.49	0.98	0.72	0.72	1.91
Chahar Mahaal and Bakhtiari	11.45	7.49	8.99	0.61	0.22	0.39	0.55	0.32
East Azarbayejan	23.74	7.05	9.58	1.30	0.79	0.83	0.19	1.17
Fars	21.21	12.45	11.85	1.00	0.54	0.80	0.73	0.21
Gilan	14.25	6.79	7.27	1.27	0.98	0.62	0.60	0.23
Golestan	17.98	9.32	8.79	1.38	2.89	1.04	0.70	0.51
Hamadan	16.29	12.42	9.67	1.22	0.59	1.35	0.32	0.17
Hormozgan	19.16	14.00	10.50	2.23	1.43	0.85	1.23	0.26
Ilam	13.25	9.45	8.34	1.24	0.59	0.67	0.61	0.68
Isfahan	19.00	8.51	9.96	0.99	0.45	0.64	0.34	0.43
Kerman	27.65	12.76	10.09	1.77	1.07	0.85	0.72	0.34
Kermanshah	20.40	14.88	11.64	1.25	1.06	0.77	0.44	0.74
Khorasan-e-Razavi	27.97	10.90	10.38	1.52	1.66	0.81	0.28	0.65
Khuzestan	22.60	9.75	10.58	1.13	1.71	0.90	1.00	0.39
Kohgiluyeh and Boyer-Ahmad	11.08	9.37	9.30	0.98	0.44	1.43	0.94	0.16
Kurdistan	14.07	13.17	8.79	1.06	0.64	0.83	0.15	0.40
Lorestan	25.48	7.70	6.63	1.41	1.22	0.88	0.77	0.57
Markazi	24.67	11.75	9.13	1.11	1.00	0.79	0.26	0.17
Mazandaran	15.42	6.48	7.86	0.94	0.71	0.61	0.72	0.33
North Khorasan	30.55	10.04	8.95	1.77	0.96	0.96	0.29	0.25
Qazvin	19.87	9.63	9.54	1.15	0.54	0.69	0.25	0.18
Qom	40.19	8.06	9.33	1.63	2.21	0.99	0.40	0.44
Semnan	25.28	12.17	11.28	0.91	0.86	0.54	0.36	0.48
Sistan and Baluchistan	27.81	12.44	9.21	3.02	4.22	2.30	1.45	0.43
South Khorasan	33.90	12.26	9.65	1.45	1.66	0.69	0.24	0.25
Tehran	16.58	7.96	8.59	0.67	1.16	0.72	0.72	0.34
West Azarbayejan	20.58	8.78	8.83	1.35	0.84	0.68	0.23	0.50
Yazd	24.63	10.27	11.45	1.20	1.09	0.61	0.20	0.67
Zanjan	33.61	10.43	8.87	1.79	1.02	1.35	0.51	0.22

Figure S8. Age-standardised mortality rates per 100 000 for communicable, maternal, neonatal, and nutritional (CMNN) diseases in Iran and 31 provinces in males in 2023.

Red and orange colours show higher rates. Yellow and green colours show lower rates.

Location	Ischemic heart disease	Stroke	Hypertensive heart disease	Other cardiovascular and circulatory	Atrial fibrillation and flutter	Cardiomyopathy and myocarditis	Non rheumatic valvular heart	Rheumatic heart disease	Aortic aneurysm	Pulmonary Arterial Hypertension	Endocarditis	Lower extremity peripheral arterial
Iran (Islamic Republic of)	127.18	47.14	28.14	5.54	4.05	1.51	1.25	1.15	0.54	0.51	0.33	0.16
Alborz	155.99	43.61	19.73	3.06	3.90	1.08	0.83	0.77	0.69	0.38	0.21	0.10
Ardebil	162.28	54.19	33.66	14.97	4.27	1.00	1.59	1.09	0.30	0.52	0.12	0.08
Bushehr	154.89	66.87	28.64	5.18	4.93	1.00	1.15	0.68	0.29	0.65	0.13	0.96
Chahar Mahaal and Bakhtiari	171.20	48.09	33.40	7.13	4.25	1.76	1.68	3.66	1.41	0.50	0.79	0.05
East Azarbayejan	145.17	60.41	56.27	7.61	5.73	2.51	1.76	1.74	1.44	0.58	0.40	0.17
Fars	115.28	47.75	28.79	5.34	3.89	0.96	0.97	0.84	0.21	0.45	0.22	0.09
Gilan	144.70	52.89	32.55	5.71	4.45	0.68	0.83	0.77	0.22	0.49	0.16	0.06
Golestan	145.73	52.22	27.01	8.31	3.38	0.95	1.03	1.16	0.29	0.44	0.20	0.15
Hamadan	149.35	49.09	25.20	5.54	4.00	1.41	1.24	0.92	0.28	0.55	0.37	0.13
Hormozgan	127.94	42.60	30.52	7.93	3.26	1.07	1.35	1.50	0.12	0.43	0.35	0.17
Ilam	123.21	34.39	24.99	5.78	3.06	0.67	1.45	0.81	0.19	0.49	0.23	0.16
Isfahan	124.04	41.59	25.23	5.33	4.10	1.17	1.50	0.81	0.71	0.47	0.57	0.21
Kerman	131.30	43.90	27.79	6.71	4.34	1.26	1.17	1.01	0.18	0.49	0.26	0.12
Kermanshah	154.89	59.74	29.73	7.21	4.37	1.54	1.36	0.82	0.33	0.59	0.44	0.06
Khorasan-e-Razavi	114.17	46.78	33.29	5.92	4.03	1.42	1.25	0.93	0.29	0.50	0.32	0.28
Khuzestan	166.70	58.57	26.46	6.73	3.74	3.09	1.54	0.97	0.49	0.56	0.45	0.17
Kohgiluyeh and Boyer-Ahmad	132.12	39.54	21.24	4.14	3.15	1.20	0.91	0.80	0.24	0.74	0.56	0.01
Kurdistan	122.90	45.69	31.68	6.39	3.69	1.23	1.24	1.18	0.39	0.54	0.25	0.14
Lorestan	162.00	54.65	42.74	4.07	4.45	3.69	1.31	0.71	0.35	0.65	0.32	0.08
Markazi	110.73	45.69	38.71	5.17	4.20	0.88	1.91	1.08	0.40	0.51	0.29	0.14
Mazandaran	125.01	46.53	37.14	4.28	4.61	0.98	0.91	0.74	0.45	0.42	0.22	0.14
North Khorasan	125.97	52.22	41.47	8.81	4.18	1.42	1.47	1.24	0.28	0.63	1.41	0.71
Qazvin	133.22	50.59	27.72	6.23	4.20	1.03	1.30	0.75	0.48	0.66	0.25	0.11
Qom	262.78	79.08	34.69	6.15	8.51	1.64	2.09	1.40	0.90	0.73	0.54	0.02
Semnan	117.54	47.96	32.06	4.32	4.03	0.98	1.23	0.64	0.59	0.40	0.25	0.06
Sistan and Baluchistan	106.21	43.94	30.53	8.06	3.09	1.38	1.55	3.03	0.47	0.78	0.65	0.01
South Khorasan	113.15	44.35	28.90	8.51	4.69	0.82	1.19	1.27	0.13	0.68	0.21	0.25
Tehran	88.14	35.26	11.12	2.30	3.30	1.13	0.98	1.42	0.86	0.40	0.21	0.14
West Azarbayejan	169.87	61.23	39.09	7.88	5.02	4.81	1.42	1.25	0.50	0.60	0.57	0.39
Yazd	94.05	44.22	30.27	4.49	3.94	1.14	1.37	0.78	0.52	0.46	0.20	0.16
Zanjan	197.10	73.02	41.54	9.57	5.84	1.95	1.89	1.08	0.26	0.78	0.38	0.20

Figure S9. Age-standardised mortality rates per 100 000 for cardiovascular diseases in Iran and 31 provinces in both sexes in 2023. Red and orange colours show higher rates. Yellow and green colours show lower rates.

Location	Ischemic heart disease	Stroke	Hypertensive heart disease	Other cardiovascular and circulatory	Atrial fibrillation and flutter	Rheumatic heart disease	Cardiomyopathy and myocarditis	Non rheumatic valvular heart	Pulmonary Arterial Hypertension	Endocarditis	Aortic aneurysm	Lower extremity peripheral arterial
Iran (Islamic Republic of)	105.27	45.15	32.13	5.81	4.79	1.34	1.30	1.29	0.61	0.32	0.27	0.17
Alborz	138.34	44.70	23.04	3.40	4.84	0.90	0.95	0.87	0.41	0.23	0.37	0.06
Ardebil	120.15	47.34	36.37	14.04	4.81	1.21	0.75	1.53	0.63	0.10	0.15	0.01
Bushehr	135.28	65.73	33.63	5.71	5.99	0.89	0.87	1.36	0.90	0.12	0.15	1.15
Chahar Mahaal and Bakhtiari	132.12	41.99	36.65	7.30	4.69	4.14	1.50	1.79	0.59	0.94	0.77	0.01
East Azarbayejan	120.57	59.40	64.58	8.29	6.97	2.09	2.30	1.77	0.76	0.41	0.86	0.21
Fars	91.60	42.62	31.30	5.24	4.46	0.99	0.73	0.99	0.54	0.21	0.10	0.05
Gilan	126.33	52.07	36.54	6.07	5.29	0.92	0.51	0.90	0.61	0.12	0.07	0.01
Golestan	116.92	48.63	28.72	7.93	3.77	1.26	0.73	1.15	0.50	0.16	0.15	0.19
Hamadan	136.02	52.79	30.74	6.29	5.03	1.16	1.23	1.35	0.63	0.39	0.14	0.07
Hormozgan	87.88	34.78	30.90	6.85	3.55	1.59	0.85	1.29	0.43	0.31	0.01	0.25
Ilam	95.24	30.13	27.43	6.12	3.48	0.99	0.48	1.36	0.60	0.24	0.03	0.02
Isfahan	106.78	41.81	29.57	5.51	4.91	0.95	0.94	1.48	0.53	0.57	0.34	0.19
Kerman	114.36	43.83	31.65	6.91	5.23	1.24	1.08	1.30	0.61	0.26	0.08	0.12
Kermanshah	123.73	57.20	33.90	7.58	5.15	0.96	1.13	1.36	0.64	0.44	0.14	0.06
Khorasan-e-Razavi	91.68	44.17	37.67	6.27	4.86	1.09	1.19	1.30	0.61	0.31	0.15	0.34
Khuzestan	138.12	56.55	29.91	6.99	4.56	1.13	2.83	1.65	0.71	0.44	0.23	0.21
Kohgiluyeh and Boyer-Ahmad	123.62	40.38	26.27	4.79	3.78	1.13	0.99	1.04	1.06	0.82	0.28	0.01
Kurdistan	96.88	42.43	35.85	6.63	4.30	1.36	1.01	1.34	0.61	0.23	0.09	0.20
Lorestan	114.48	43.52	43.99	4.06	4.79	0.86	3.38	1.15	0.73	0.26	0.16	0.11
Markazi	81.22	40.42	40.63	5.12	4.42	1.31	0.57	1.80	0.53	0.25	0.23	0.05
Mazandaran	109.52	45.82	42.71	4.70	5.37	0.85	0.85	1.05	0.48	0.19	0.26	0.17
North Khorasan	118.64	56.09	50.68	9.51	5.40	1.52	1.19	1.74	0.87	1.77	0.27	0.55
Qazvin	107.77	48.38	32.63	6.45	4.94	0.95	0.74	1.39	0.81	0.22	0.28	0.11
Qom	228.73	74.27	37.69	6.60	9.55	1.89	1.34	2.21	0.80	0.53	0.43	0.02
Semnan	89.53	43.50	36.66	4.71	4.73	0.85	0.70	1.26	0.47	0.25	0.22	0.01
Sistan and Baluchistan	92.11	41.09	32.19	7.74	3.42	3.21	1.26	1.58	0.97	0.67	0.34	0.02
South Khorasan	94.25	42.65	34.82	8.95	5.60	1.66	0.64	1.46	0.79	0.11	0.11	0.20
Tehran	70.46	33.27	13.02	2.53	3.94	1.59	0.85	0.95	0.51	0.13	0.37	0.12
West Azarbayejan	150.90	62.28	45.64	8.68	5.94	1.55	4.88	1.59	0.74	0.65	0.32	0.48
Yazd	76.97	43.63	35.66	4.88	4.81	0.97	0.99	1.34	0.62	0.13	0.14	0.17
Zanjan	165.20	68.13	48.02	10.08	6.92	1.41	1.53	2.19	0.87	0.32	0.13	0.38

Figure S10. Age-standardised mortality rates per 100 000 for cardiovascular diseases in Iran and 31 provinces in females in 2023. Red and orange colours show higher rates. Yellow and green colours show lower rates.

Location	Ischemic heart disease	Stroke	Hypertensive heart disease	Other cardiovascular and circulatory	Atrial fibrillation and flutter	Cardiomyopathy and myocarditis	Non rheumatic valvular heart	Rheumatic heart disease	Aortic aneurysm	Pulmonary Arterial Hypertension	Endocarditis	Lower extremity peripheral arterial
Iran (Islamic Republic of)	150.70	49.32	23.67	5.24	3.21	1.73	1.20	0.95	0.84	0.39	0.35	0.16
Alborz	174.80	42.79	16.65	2.74	3.01	1.21	0.80	0.64	1.01	0.34	0.18	0.13
Ardebil	217.39	63.92	30.48	16.20	3.66	1.31	1.67	0.97	0.47	0.39	0.15	0.15
Bushehr	178.49	68.88	22.78	4.58	3.67	1.13	0.92	0.44	0.44	0.37	0.15	0.69
Chahar Mahaal and Bakhtiari	223.51	56.78	29.07	6.91	3.67	2.08	1.54	3.09	2.19	0.38	0.61	0.09
East Azarbayejan	171.57	61.48	46.78	6.83	4.30	2.73	1.75	1.36	2.05	0.39	0.39	0.11
Fars	143.35	54.14	25.70	5.44	3.21	1.23	0.95	0.66	0.34	0.35	0.23	0.12
Gilan	165.22	53.88	28.15	5.31	3.52	0.86	0.76	0.60	0.37	0.35	0.19	0.10
Golestan	182.93	57.37	24.76	8.82	2.87	1.21	0.89	1.06	0.45	0.37	0.25	0.09
Hamadan	162.69	45.02	19.19	4.73	2.87	1.59	1.12	0.66	0.43	0.48	0.36	0.19
Hormozgan	175.53	52.46	29.98	9.16	2.90	1.32	1.42	1.40	0.25	0.43	0.39	0.06
Ilam	152.72	38.85	22.26	5.42	2.63	0.86	1.55	0.62	0.35	0.37	0.22	0.31
Isfahan	141.76	41.36	20.72	5.12	3.25	1.40	1.51	0.67	1.08	0.40	0.57	0.22
Kerman	149.86	43.87	23.01	6.46	3.22	1.45	1.03	0.76	0.30	0.36	0.27	0.12
Kermanshah	188.32	62.34	24.83	6.76	3.47	1.98	1.36	0.65	0.53	0.53	0.44	0.06
Khorasan-e-Razavi	138.13	49.66	28.70	5.56	3.15	1.65	1.20	0.75	0.43	0.38	0.33	0.21
Khuzestan	197.26	60.85	22.65	6.42	2.84	3.36	1.43	0.80	0.77	0.40	0.47	0.13
Kohgiluyeh and Boyer-Ahmad	141.08	38.83	16.19	3.49	2.54	1.41	0.78	0.47	0.21	0.41	0.30	0.00
Kurdistan	150.10	49.08	27.26	6.14	3.05	1.47	1.14	0.99	0.71	0.47	0.28	0.07
Lorestan	222.98	69.40	41.10	4.10	4.03	4.07	1.51	0.54	0.57	0.54	0.40	0.04
Markazi	148.74	53.03	36.29	5.25	3.94	1.25	2.06	0.80	0.62	0.49	0.34	0.26
Mazandaran	142.10	47.34	30.66	3.79	3.72	1.12	0.76	0.61	0.65	0.36	0.24	0.11
North Khorasan	133.91	48.72	32.78	8.17	3.06	1.65	1.22	0.96	0.30	0.39	1.05	0.87
Qazvin	162.54	53.38	21.83	5.97	3.26	1.36	1.20	0.52	0.68	0.49	0.29	0.11
Qom	307.07	86.11	31.46	5.68	7.29	2.00	1.99	0.82	1.45	0.65	0.56	0.01
Semnan	147.44	53.10	26.87	3.89	3.19	1.28	1.20	0.41	0.97	0.32	0.26	0.11
Sistan and Baluchistan	120.51	46.76	28.76	8.37	2.75	1.51	1.52	2.84	0.59	0.59	0.64	0.00
South Khorasan	133.40	46.13	22.31	8.00	3.65	1.01	0.88	0.84	0.16	0.55	0.32	0.30
Tehran	106.38	37.41	9.22	2.07	2.65	1.41	1.02	1.26	1.36	0.30	0.28	0.17
West Azarbayejan	191.62	60.21	31.06	6.97	3.85	4.72	1.24	0.91	0.70	0.43	0.48	0.28
Yazd	111.28	44.71	24.15	4.03	2.94	1.28	1.40	0.58	0.91	0.27	0.28	0.13
Zanjan	233.64	79.08	34.23	9.04	4.58	2.42	1.56	0.72	0.40	0.68	0.44	0.01

Figure S11. Age-standardised mortality rates per 100 000 for cardiovascular diseases in Iran and 31 provinces in males in 2023. Red and orange colours show higher rates. Yellow and green colours show lower rates.

Location	Stomach cancer	lung cancer	Colon cancer	Leukemia	Prostate cancer	Brain cancer	Breast cancer	Pancreatic cancer	Esophageal cancer	Liver cancer
Iran (Islamic Republic of)	14.05	11.63	8.32	6.66	6.39	5.75	4.70	4.11	3.90	3.59
Alborz	11.29	9.97	8.85	5.08	6.39	6.13	5.27	4.63	2.90	1.73
Ardebil	27.35	14.90	9.40	7.96	5.93	6.00	3.97	3.76	10.58	6.15
Bushehr	8.56	14.79	6.80	7.45	7.05	5.93	5.30	2.89	1.78	3.75
Chahar Mahaal and Bakhtiari	17.55	11.35	6.72	7.06	5.61	5.76	2.71	2.16	1.70	5.03
East Azarbayejan	22.79	15.57	13.08	7.62	7.98	6.77	5.29	5.09	8.07	4.09
Fars	8.60	8.43	5.21	6.10	5.12	5.01	3.71	2.51	1.25	2.78
Gilan	14.65	10.68	8.77	7.22	6.54	5.29	4.71	3.01	3.04	3.10
Golestan	12.77	9.69	7.39	6.02	4.03	5.17	4.19	2.86	7.89	4.12
Hamadan	14.52	11.36	6.77	6.92	5.56	4.51	4.01	2.97	2.41	4.21
Hormozgan	7.63	9.39	4.78	5.18	4.52	4.10	3.89	1.59	0.98	1.82
Ilam	20.49	11.95	7.95	6.02	5.68	4.98	3.24	3.64	6.65	6.06
Isfahan	9.11	10.04	7.01	6.94	6.88	5.84	4.11	3.91	1.92	3.32
Kerman	10.01	13.55	5.87	6.20	5.73	5.81	4.15	2.82	1.32	3.44
Kermanshah	13.36	13.10	7.57	6.64	6.38	6.16	4.44	4.63	4.07	5.59
Khorasan-e-Razavi	16.67	12.19	7.73	6.80	5.72	5.27	3.59	3.71	6.12	4.12
Khuzestan	9.76	11.75	7.67	6.01	5.26	5.57	5.73	4.30	2.07	3.88
Kohgiluyeh and Boyer-Ahmad	17.41	9.70	3.93	7.00	5.13	5.47	2.82	2.83	1.83	2.95
Kurdistan	26.32	16.71	10.50	7.21	8.34	5.86	3.90	4.01	7.62	4.76
Lorestan	20.07	13.50	9.01	6.73	6.74	7.30	4.01	4.88	6.29	5.50
Markazi	13.54	10.17	7.45	7.15	6.25	6.82	3.91	3.90	2.45	3.39
Mazandaran	16.49	9.24	8.07	6.10	7.08	5.11	5.04	2.93	4.26	3.32
North Khorasan	20.69	11.67	8.72	6.76	5.25	6.06	4.38	3.78	8.80	4.99
Qazvin	17.86	12.23	10.58	6.97	7.07	7.80	5.44	4.79	4.43	2.95
Qom	21.10	17.74	15.73	11.42	11.15	9.67	8.79	8.29	6.70	5.10
Semnan	13.51	11.65	9.73	6.20	6.36	5.22	5.24	4.53	4.34	3.26
Sistan and Baluchistan	11.82	9.72	4.97	5.45	4.33	4.34	3.83	1.72	2.64	4.28
South Khorasan	13.32	10.38	7.40	6.73	6.19	5.46	4.04	2.98	4.99	3.31
Tehran	9.70	10.22	9.16	6.29	6.99	5.43	5.52	5.63	2.41	2.65
West Azarbayejan	27.93	18.89	10.49	7.21	7.30	6.65	5.31	5.21	9.68	5.32
Yazd	8.12	12.57	9.11	7.41	6.84	7.81	5.55	5.53	2.36	2.91
Zanjan	28.65	18.28	12.81	9.52	7.85	7.85	5.14	4.83	6.52	5.34

Figure S12. Age-standardised mortality rates per 100 000 for top 10 neoplasms in Iran and 31 provinces in both sexes in 2023. Red and orange colours show higher rates. Yellow and green colours show lower rates.

Location	Stomach cancer	Breast cancer	lung cancer	Colon cancer	Leukemia	Brain cancer	Pancreatic cancer	Liver cancer	Esophageal cancer
Iran (Islamic Republic of)	9.95	9.17	7.20	7.12	5.22	5.11	3.18	3.13	3.10
Alborz	8.14	10.48	6.50	7.45	4.05	5.62	3.70	1.60	2.46
Ardebil	20.45	7.68	9.05	7.99	6.38	5.31	2.99	5.00	7.97
Bushehr	7.12	10.51	11.34	6.28	6.63	5.68	2.29	3.55	1.74
Chahar Mahaal and Bakhtiari	12.39	5.21	7.38	5.98	5.75	4.96	1.28	3.99	1.41
East Azarbayejan	17.01	10.31	10.22	11.77	6.39	6.22	4.27	3.84	6.60
Fars	5.71	7.19	4.93	4.44	4.60	4.53	1.86	2.30	0.96
Gilan	11.11	9.14	6.07	7.58	5.97	4.83	2.60	2.54	2.16
Golestan	9.03	7.94	6.07	6.26	5.14	4.83	2.14	3.19	6.09
Hamadan	10.44	7.89	6.52	6.41	5.77	4.19	2.48	3.94	2.01
Hormozgan	4.79	7.58	4.99	4.09	3.78	4.08	1.18	1.27	0.87
Ilam	16.00	6.27	7.15	6.75	3.90	4.52	2.98	5.44	5.06
Isfahan	6.46	8.12	6.59	6.19	5.32	5.29	3.08	3.23	1.50
Kerman	7.55	8.17	9.00	5.41	4.96	5.37	2.23	3.25	1.12
Kermanshah	9.91	8.53	7.74	6.75	5.17	5.44	3.59	4.88	3.40
Khorasan-e-Razavi	10.72	7.03	8.64	6.38	5.51	4.61	2.90	3.65	4.52
Khuzestan	6.92	11.32	7.06	6.62	4.83	5.13	3.63	3.41	1.60
Kohgiluyeh and Boyer-Ahmad	13.77	5.60	7.08	3.90	5.70	4.67	2.31	2.30	1.59
Kurdistan	16.92	7.60	9.11	8.14	5.88	5.52	2.77	3.97	6.01
Lorestan	13.74	7.55	8.34	7.25	5.01	6.30	3.34	4.15	4.97
Markazi	9.45	7.49	6.37	6.35	5.23	5.69	3.21	3.03	1.86
Mazandaran	12.34	9.77	5.83	6.96	4.82	4.70	2.33	2.85	3.35
North Khorasan	14.46	8.57	8.07	8.21	5.58	6.06	3.46	4.86	7.20
Qazvin	12.14	10.66	6.83	8.89	5.40	7.15	4.15	2.46	3.53
Qom	16.10	17.00	12.64	13.79	8.60	8.67	6.83	4.35	5.48
Semnan	8.80	10.30	7.15	8.39	4.44	4.69	3.68	2.77	3.58
Sistan and Baluchistan	9.29	7.56	6.71	4.60	4.48	3.96	1.23	3.77	2.45
South Khorasan	9.43	7.78	6.91	6.44	4.94	5.04	2.35	3.36	3.62
Tehran	5.88	10.84	5.45	7.34	4.54	4.34	3.95	2.11	1.90
West Azarbayejan	22.78	10.33	12.15	9.77	6.08	6.04	4.69	5.02	7.87
Yazd	5.49	11.09	8.43	7.91	6.20	6.96	4.73	2.62	1.96
Zanjan	20.59	9.94	12.09	11.86	7.96	7.30	3.75	4.57	5.38

Figure S13. Age-standardised mortality rates per 100 000 for top 10 neoplasms in Iran and 31 provinces in females in 2023.

Red and orange colours show higher rates. Yellow and green colours show lower rates.

Location	Stomach cancer	lung cancer	Prostate cancer	Colon cancer	Leukemia	Brain cancer	Pancreatic cancer	Esophageal cancer	Liver cancer
Iran (Islamic Republic of)	18.45	16.34	13.52	9.60	8.19	6.41	5.09	4.76	4.08
Alborz	14.48	13.47	12.74	10.29	6.11	6.63	5.57	3.36	1.88
Ardebil	35.82	21.71	13.73	11.15	9.95	6.80	4.67	13.80	7.56
Bushehr	10.14	18.54	15.47	7.39	8.38	6.15	3.55	1.84	4.00
Chahar Mahaal and Bakhtiari	23.71	15.98	13.06	7.64	8.69	6.64	3.18	2.04	6.29
East Azarbayejan	29.00	21.28	17.00	14.48	8.98	7.36	5.95	9.68	4.35
Fars	11.89	12.34	11.39	6.09	7.78	5.51	3.22	1.59	3.31
Gilan	18.45	15.60	13.87	10.03	8.55	5.74	3.44	3.99	3.70
Golestan	17.45	14.08	9.56	8.82	7.13	5.57	3.74	10.15	5.24
Hamadan	18.80	16.40	11.59	7.13	8.11	4.83	3.47	2.81	4.48
Hormozgan	10.89	14.32	10.21	5.57	6.77	4.12	2.05	1.10	2.44
Ilam	25.24	17.08	11.88	9.19	8.18	5.43	4.33	8.33	6.71
Isfahan	11.81	13.54	14.07	7.83	8.59	6.38	4.76	2.34	3.40
Kerman	12.78	18.53	12.70	6.37	7.59	6.25	3.47	1.56	3.64
Kermanshah	17.18	19.06	13.69	8.44	8.27	6.93	5.76	4.80	6.35
Khorasan-e-Razavi	22.90	15.90	11.78	9.15	8.15	5.95	4.55	7.80	4.62
Khuzestan	12.79	16.74	11.18	8.81	7.29	6.02	5.02	2.58	4.40
Kohgiluyeh and Boyer-Ahmad	21.14	12.37	10.46	3.97	8.30	6.25	3.35	2.07	3.62
Kurdistan	36.33	24.76	17.18	12.99	8.61	6.22	5.31	9.34	5.61
Lorestan	27.90	19.76	15.74	11.16	8.85	8.42	6.74	7.93	7.16
Markazi	18.61	14.66	14.83	8.81	9.50	8.07	4.72	3.18	3.83
Mazandaran	21.11	13.00	15.33	9.30	7.52	5.54	3.58	5.30	3.84
North Khorasan	26.98	15.35	10.30	9.24	7.93	6.06	4.11	10.42	5.14
Qazvin	24.13	18.06	15.36	12.44	8.71	8.47	5.45	5.42	3.48
Qom	26.87	23.45	25.09	18.02	14.68	10.73	9.92	8.14	5.98
Semnan	18.40	16.29	13.26	11.10	8.04	5.77	5.40	5.13	3.77
Sistan and Baluchistan	14.44	12.87	8.80	5.36	6.46	4.73	2.22	2.82	4.81
South Khorasan	17.62	14.20	13.04	8.44	8.63	5.90	3.67	6.48	3.26
Tehran	13.59	15.09	14.12	11.02	8.06	6.53	7.34	2.93	3.20
West Azarbayejan	33.89	26.51	16.31	11.37	8.59	7.33	5.81	11.82	5.65
Yazd	10.79	16.74	14.34	10.35	8.65	8.62	6.34	2.78	3.20
Zanjan	37.54	25.05	16.83	13.84	11.28	8.44	5.99	7.80	6.20

Figure S14. Age-standardised mortality rates per 100 000 for top 10 neoplasms in Iran and 31 provinces in males in 2023.

Red and orange colours show higher rates. Yellow and green colours show lower rates.

Location	COPD	CKD	Diabetes	Cirrhosis	Drug use disorders
Iran (Islamic Republic of)	21.89	20.45	20.38	10.07	3.13
Alborz	19.11	16.81	21.05	9.86	3.77
Ardebil	12.88	24.99	17.22	8.09	2.35
Bushehr	18.13	20.57	23.06	12.61	2.77
Chahar Mahaal and Bakhtiari	13.14	16.21	14.71	5.71	1.49
East Azarbayejan	34.02	29.79	20.14	12.44	3.17
Fars	19.23	17.55	15.04	10.59	3.96
Gilan	17.39	20.63	19.67	8.46	2.22
Golestan	17.15	21.10	22.09	11.95	1.63
Hamadan	20.00	21.38	11.44	10.34	5.82
Hormozgan	11.29	22.86	14.48	11.34	3.29
Ilam	19.04	30.28	14.21	9.93	1.14
Isfahan	22.43	18.55	22.14	9.65	2.02
Kerman	48.80	23.47	15.48	11.05	2.72
Kermanshah	24.55	22.10	14.46	9.44	6.90
Khorasan-e-Razavi	24.80	19.91	16.58	11.96	3.37
Khuzestan	15.06	18.48	28.91	10.68	3.81
Kohgiluyeh and Boyer-Ahmad	10.16	16.36	10.52	5.69	1.71
Kurdistan	23.29	23.22	14.88	10.54	1.83
Lorestan	19.11	21.57	16.76	10.37	5.87
Markazi	23.29	20.27	16.37	10.20	2.99
Mazandaran	16.39	19.75	24.89	7.37	1.18
North Khorasan	33.11	23.98	15.80	13.28	2.70
Qazvin	23.51	23.06	15.95	16.67	5.75
Qom	42.21	35.63	28.89	17.64	2.40
Semnan	21.95	22.82	21.27	10.22	2.64
Sistan and Baluchistan	22.28	30.81	18.05	11.98	4.76
South Khorasan	29.87	20.85	14.66	13.79	2.78
Tehran	16.82	16.21	26.79	7.36	3.11
West Azarbayejan	28.24	22.65	17.37	11.00	1.37
Yazd	34.47	20.86	28.62	9.84	2.51
Zanjan	35.46	30.86	17.22	17.84	4.26

Figure S15. Age-standardised mortality rates per 100 000 for top 5 non-communicable diseases in Iran and 31 provinces in both sexes in 2023

Red and orange colours show higher rates. Yellow and green colours show lower rates.

Location	CKD	Diabetes	COPD	Cirrhosis	Drug use disorders
Iran (Islamic Republic of)	20.18	18.37	17.55	8.19	1.03
Alborz	17.15	20.77	15.57	8.25	1.12
Ardebil	25.05	16.26	10.18	6.88	0.97
Bushehr	20.81	22.13	15.16	11.77	0.94
Chahar Mahaal and Bakhtiari	15.75	13.36	10.94	4.28	0.52
East Azarbayejan	30.68	19.36	28.36	11.22	1.03
Fars	16.82	14.32	14.67	7.50	1.32
Gilan	21.54	20.06	13.39	7.09	0.77
Golestan	21.20	21.27	12.95	9.22	0.72
Hamadan	21.83	12.77	16.31	8.50	1.74
Hormozgan	21.35	13.38	8.49	8.89	1.15
Ilam	30.09	13.82	14.33	8.49	0.29
Isfahan	18.23	19.68	18.61	8.38	0.50
Kerman	24.53	15.92	37.70	8.65	1.21
Kermanshah	21.55	14.67	18.76	7.56	2.00
Khorasan-e-Razavi	19.52	15.99	22.31	9.87	1.11
Khuzestan	18.05	23.88	11.79	8.48	1.19
Kohgiluyeh and Boyer-Ahmad	16.20	11.79	7.52	4.49	0.79
Kurdistan	22.18	15.65	18.30	9.14	0.53
Lorestan	20.09	15.30	14.96	7.45	1.76
Markazi	19.15	15.65	17.48	8.12	0.87
Mazandaran	20.23	22.63	13.02	6.55	0.46
North Khorasan	24.57	17.76	29.42	12.55	1.05
Qazvin	23.14	16.73	17.61	13.83	1.60
Qom	36.22	26.12	31.83	14.25	0.83
Semnan	22.74	18.47	16.65	7.33	0.89
Sistan and Baluchistan	30.00	17.57	19.77	10.18	2.51
South Khorasan	20.86	14.76	26.55	11.86	1.30
Tehran	15.03	20.57	13.33	5.28	0.87
West Azarbayejan	22.93	18.01	20.39	10.21	0.51
Yazd	20.77	22.99	24.97	8.78	0.74
Zanjan	31.42	17.78	30.78	14.34	1.32

Figure S16. Age-standardised mortality rates per 100 000 for top 5 non-communicable diseases in Iran and 31 provinces in females in 2023

Red and orange colours show higher rates. Yellow and green colours show lower rates.

Location	COPD	Diabetes	CKD	Cirrhosis	Drug use disorders
Iran (Islamic Republic of)	26.67	22.51	20.76	12.01	5.17
Alborz	22.72	21.38	16.54	11.50	6.36
Ardebil	16.32	18.44	25.34	9.51	3.66
Bushehr	21.60	24.02	20.41	13.37	4.47
Chahar Mahaal and Bakhtiari	16.15	16.45	16.87	7.41	2.42
East Azarbayejan	40.40	20.98	28.85	13.67	5.23
Fars	24.67	15.88	18.48	13.98	6.50
Gilan	21.75	19.20	19.62	9.86	3.61
Golestan	22.75	23.11	21.21	15.22	2.53
Hamadan	23.85	9.98	20.90	12.18	9.74
Hormozgan	14.73	15.73	24.68	14.03	5.32
Ilam	23.81	14.46	30.45	11.35	1.96
Isfahan	26.39	24.65	18.89	10.90	3.51
Kerman	61.94	14.92	22.26	13.66	4.10
Kermanshah	30.99	14.16	22.63	11.46	11.71
Khorasan-e-Razavi	27.44	17.18	20.33	14.10	5.58
Khuzestan	18.66	34.26	19.07	12.94	6.37
Kohgiluyeh and Boyer-Ahmad	12.91	9.23	16.65	6.89	2.56
Kurdistan	28.59	14.07	24.33	12.00	3.06
Lorestan	24.42	18.52	23.54	13.87	9.89
Markazi	30.88	17.30	22.02	12.61	5.07
Mazandaran	20.27	27.43	19.24	8.21	1.89
North Khorasan	36.95	13.91	23.32	14.07	4.31
Qazvin	30.50	15.07	22.97	19.77	9.73
Qom	55.04	32.25	35.33	21.35	3.94
Semnan	28.07	24.33	22.86	13.25	4.31
Sistan and Baluchistan	24.94	18.55	31.62	13.86	6.94
South Khorasan	33.52	14.53	20.85	15.89	4.18
Tehran	20.47	33.16	17.44	9.49	5.36
West Azarbayejan	37.99	16.66	22.55	11.85	2.21
Yazd	44.73	34.31	21.04	10.82	4.19
Zanjan	40.87	16.56	30.35	21.63	7.09

Figure S17. Age-standardised mortality rates per 100 000 for top 5 non-communicable diseases in Iran and 31 provinces in males in 2023.

Red and orange colours show higher rates. Yellow and green colours show lower rates.

Location	Transport injuries	Self-harm	Falls	Interpersonal violence	Fire heat and hot substances	Exposure to mechanical	Adverse effects of medical	Drowning	Poisonings	Electrocution
Iran (Islamic Republic of)	18.90	5.41	4.17	2.61	2.58	2.47	1.22	1.01	0.73	0.57
Alborz	13.07	6.00	4.55	2.64	1.96	1.24	0.94	0.89	1.26	0.53
Ardebil	22.16	7.22	3.93	3.15	2.15	1.87	1.63	1.09	1.24	0.64
Bushehr	24.15	4.89	4.63	3.22	2.41	1.24	1.34	1.27	0.34	0.70
Chahar Mahaal and Bakhtiari	26.46	6.43	5.89	3.48	1.97	1.86	1.14	1.46	0.73	0.57
East Azarbayejan	24.15	5.58	4.75	1.67	3.26	1.77	1.29	0.59	1.16	0.49
Fars	19.76	6.32	4.12	3.43	2.77	1.10	1.25	1.06	0.67	0.62
Gilan	18.48	5.92	3.23	1.97	3.73	1.63	1.36	1.95	0.42	0.49
Golestan	23.85	6.53	3.30	2.93	1.92	2.77	1.41	0.87	0.60	0.59
Hamadan	23.32	7.46	5.51	2.38	2.32	2.12	1.34	0.71	0.91	0.62
Hormozgan	26.27	5.81	3.97	2.83	2.04	2.28	1.08	1.69	0.52	0.83
Ilam	26.10	11.66	3.92	5.13	2.91	2.05	1.44	1.45	0.49	0.47
Isfahan	14.83	3.30	4.02	1.73	2.47	2.38	1.20	0.78	0.67	0.39
Kerman	26.64	6.41	3.60	3.51	2.49	2.60	1.47	0.78	0.69	0.82
Kermanshah	21.17	10.50	5.23	4.10	4.01	1.84	1.58	0.94	0.87	0.58
Khorasan-e-Razavi	18.88	4.41	4.66	2.23	2.87	1.93	1.49	0.54	0.86	0.59
Khuzestan	18.45	6.32	2.86	4.04	3.32	3.26	1.11	2.60	0.44	0.96
Kohgiluyeh and Boyer-Ahmad	23.47	9.08	5.16	3.92	2.38	2.80	1.29	1.73	0.79	0.58
Kurdistan	24.27	6.01	5.65	2.82	2.52	2.15	1.35	1.21	0.70	0.60
Lorestan	27.81	11.98	4.06	3.82	2.68	2.71	1.30	1.51	0.87	0.67
Markazi	16.84	4.10	4.99	1.97	2.06	2.11	1.38	0.74	0.72	0.49
Mazandaran	18.13	3.57	4.00	1.82	2.15	1.21	1.03	1.60	0.50	0.48
North Khorasan	24.63	5.74	5.51	3.36	2.06	2.56	1.88	0.81	1.21	0.58
Qazvin	18.93	4.31	6.55	2.28	2.26	1.47	1.37	1.25	1.19	0.74
Qom	13.45	4.94	5.65	2.47	2.54	5.94	1.40	0.61	0.60	0.49
Semnan	15.17	3.31	4.09	1.75	1.60	1.57	1.03	0.63	0.93	0.52
Sistan and Baluchistan	42.77	7.03	3.74	6.94	4.41	4.54	2.38	1.79	0.51	1.10
South Khorasan	17.49	4.03	4.17	1.78	3.22	1.68	1.40	0.53	0.88	0.56
Tehran	6.49	3.16	3.20	0.93	1.74	3.60	0.66	0.28	0.59	0.23
West Azarbayejan	20.98	6.86	4.36	3.38	3.10	2.55	1.29	0.91	0.56	0.54
Yazd	16.40	2.87	4.03	1.22	2.66	1.91	1.27	0.60	0.63	0.48
Zanjan	33.00	6.14	8.63	3.22	3.36	2.80	1.55	1.17	1.83	0.86

Figure S18. Age-standardised mortality rates per 100 000 for top 10 injuries in Iran and 31 provinces in both sexes in 2023. Red and orange colours show higher rates. Yellow and green colours show lower rates.

Location	Transport injuries	Self-harm	Falls	Fire heat and hot substances	Interpersonal violence	Exposure to mechanical	Adverse effects of medical	Poisonings	Drowning	Electrocution
Iran (Islamic Republic of)	10.05	3.16	2.98	2.52	1.13	1.09	0.97	0.51	0.45	0.13
Alborz	6.87	2.82	2.89	1.80	1.11	0.61	0.79	0.82	0.58	0.08
Ardebil	13.85	5.01	2.35	2.36	1.82	1.03	1.23	1.09	0.65	0.20
Bushehr	11.20	3.92	3.67	2.61	1.76	0.59	1.13	0.29	0.61	0.15
Chahar Mahaal and Bakhtiari	16.82	5.32	3.44	1.85	1.42	1.08	0.80	0.55	0.63	0.21
East Azarbayejan	13.46	2.86	3.56	3.27	0.80	0.83	1.08	0.87	0.22	0.12
Fars	9.55	3.32	2.67	2.85	1.21	0.47	0.96	0.46	0.51	0.14
Gilan	8.98	2.87	2.03	3.20	0.82	0.75	1.04	0.30	0.95	0.06
Golestan	11.40	5.02	1.95	1.99	1.58	1.32	1.18	0.41	0.31	0.06
Hamadan	13.47	3.23	3.90	2.33	1.02	1.04	0.97	0.60	0.33	0.18
Hormozgan	13.44	2.62	2.28	1.95	1.14	1.09	0.79	0.51	0.90	0.20
Ilam	13.77	8.48	2.57	3.17	2.88	0.87	1.17	0.36	0.51	0.11
Isfahan	6.98	1.30	3.09	2.34	0.67	1.02	0.97	0.42	0.27	0.08
Kerman	15.62	3.61	2.52	2.54	1.31	1.31	1.12	0.57	0.45	0.21
Kermanshah	10.80	7.03	3.68	4.45	2.18	0.80	1.28	0.61	0.30	0.13
Khorasan-e-Razavi	9.65	2.54	3.15	2.96	0.91	0.97	1.24	0.59	0.22	0.12
Khuzestan	9.08	4.14	1.71	3.40	2.10	1.58	0.88	0.30	1.23	0.24
Kohgiluyeh and Boyer-Ahmad	13.98	7.80	3.51	3.03	1.98	1.47	1.21	0.57	0.86	0.21
Kurdistan	13.97	4.39	3.75	2.94	1.31	1.00	1.02	0.43	0.49	0.18
Lorestan	15.19	10.06	2.68	2.79	1.99	1.34	0.98	0.72	0.50	0.15
Markazi	8.84	1.31	3.49	2.04	0.77	0.94	1.14	0.58	0.41	0.00
Mazandaran	9.80	1.96	2.91	2.05	0.76	0.57	0.86	0.35	0.54	0.05
North Khorasan	13.41	4.56	3.99	2.44	1.42	1.13	1.62	0.81	0.32	0.11
Qazvin	10.04	2.36	4.97	2.08	0.85	0.72	1.16	0.96	0.63	0.17
Qom	6.85	1.02	4.23	2.18	0.81	2.51	1.17	0.39	0.30	0.14
Semnan	7.64	1.18	2.91	1.64	0.62	0.93	0.81	0.62	0.39	0.13
Sistan and Baluchistan	25.75	4.89	2.55	4.39	2.47	2.06	1.73	0.54	0.72	0.46
South Khorasan	9.66	2.41	2.79	3.31	0.66	0.87	1.19	0.80	0.25	0.15
Tehran	2.72	1.30	2.82	1.27	0.27	1.18	0.54	0.29	0.08	0.01
West Azarbayejan	12.15	5.45	3.15	3.52	1.96	1.30	1.03	0.41	0.46	0.15
Yazd	8.50	1.40	2.88	2.39	0.34	0.82	1.04	0.39	0.32	0.05
Zanjan	20.54	3.59	6.21	3.14	1.67	1.49	1.12	1.50	0.61	0.19

Figure S19. Age-standardised mortality rates per 100 000 for top 10 injuries in Iran and 31 provinces in females in 2023.

Red and orange colours show higher rates. Yellow and green colours show lower rates.

Location	Transport injuries	Self-harm	Falls	Interpersonal violence	Exposure to mechanical	Fire heat and hot substances	Drowning	Adverse effects of medical	Electrocution	Poisonings
Iran (Islamic Republic of)	27.58	7.59	5.36	4.04	3.82	2.64	1.54	1.47	0.99	0.94
Alborz	19.12	9.09	6.20	4.13	1.86	2.11	1.20	1.09	0.97	1.69
Ardebil	30.36	9.36	5.67	4.39	2.69	1.95	1.52	2.08	1.06	1.39
Bushehr	36.51	5.78	5.55	4.53	1.85	2.20	1.89	1.55	1.19	0.39
Chahar Mahaal and Bakhtiari	36.13	7.52	8.56	5.43	2.61	2.10	2.27	1.52	0.92	0.92
East Azarbayejan	34.57	8.20	5.94	2.48	2.67	3.26	0.93	1.52	0.84	1.45
Fars	29.75	9.19	5.62	5.52	1.71	2.68	1.57	1.56	1.07	0.88
Gilan	27.83	8.89	4.45	3.08	2.50	4.25	2.91	1.68	0.91	0.53
Golestan	36.50	8.04	4.77	4.24	4.22	1.84	1.42	1.67	1.10	0.79
Hamadan	32.88	11.52	7.09	3.67	3.17	2.30	1.07	1.71	1.03	1.22
Hormozgan	38.85	8.88	5.73	4.43	3.43	2.13	2.45	1.38	1.43	0.54
Ilam	38.12	14.69	5.27	7.25	3.19	2.63	2.35	1.70	0.81	0.62
Isfahan	22.53	5.25	4.93	2.76	3.71	2.60	1.26	1.42	0.69	0.91
Kerman	37.09	9.01	4.64	5.52	3.80	2.43	1.09	1.84	1.38	0.80
Kermanshah	31.38	13.93	6.79	5.95	2.86	3.56	1.56	1.90	1.00	1.13
Khorasan-e-Razavi	28.05	6.25	6.18	3.51	2.88	2.78	0.86	1.74	1.06	1.13
Khuzestan	27.59	8.45	4.02	5.92	4.90	3.22	3.92	1.35	1.66	0.57
Kohgiluyeh and Boyer-Ahmad	32.40	10.29	6.76	5.73	4.06	1.76	2.55	1.37	0.93	1.01
Kurdistan	34.29	7.55	7.54	4.25	3.27	2.09	1.90	1.68	1.00	0.97
Lorestan	40.49	13.89	5.52	5.56	4.06	2.56	2.49	1.65	1.16	1.02
Markazi	24.94	6.85	6.64	3.12	3.28	2.08	1.07	1.67	0.97	0.86
Mazandaran	26.47	5.17	5.11	2.85	1.83	2.24	2.65	1.21	0.90	0.65
North Khorasan	35.54	6.90	7.01	5.23	3.94	1.70	1.27	2.12	1.03	1.60
Qazvin	27.65	6.18	8.10	3.65	2.19	2.45	1.84	1.59	1.29	1.40
Qom	20.20	8.86	7.20	4.10	9.41	2.93	0.92	1.67	0.83	0.81
Semnan	22.50	5.36	5.24	2.82	2.19	1.56	0.87	1.26	0.90	1.24
Sistan and Baluchistan	59.33	9.10	4.91	11.23	6.95	4.44	2.81	3.03	1.72	0.48
South Khorasan	25.03	5.57	5.55	2.83	2.44	3.12	0.80	1.61	0.93	0.97
Tehran	10.26	5.01	3.58	1.60	6.01	2.23	0.48	0.79	0.46	0.90
West Azarbayejan	29.78	8.25	5.64	4.74	3.77	2.68	1.34	1.57	0.90	0.72
Yazd	23.99	4.25	5.15	2.05	2.94	2.92	0.87	1.50	0.89	0.85
Zanjan	45.36	8.61	11.09	4.69	4.08	3.58	1.71	1.99	1.50	2.15

Figure S20. Age-standardised mortality rates per 100 000 for top 10 injuries in Iran and 31 provinces in males in 2023.

Red and orange colours show higher rates. Yellow and green colours show lower rates.

Table S1. All-cause mortality counts and age-standardised mortality rates per 100 000 across 31 provinces in both sexes combined in 2019, 2020, and 2023

	2019		2020		2023	
**Province**	**Counts**	**Rates**	**Counts**	**Rates**	**Counts**	**Rates**
Alborz	10941 (10694, 11188)	548.2 (535.9, 560.8)	16216 (15869, 16576)	804.3 (784.6, 822.9)	12108 (11471, 12822)	540.2 (509.7, 573.2)
Ardebil	6818 (6664, 6971)	635.9 (621.3, 651)	9462 (9263, 9698)	901.3 (879.8, 923.7)	6694 (6343, 7053)	613.8 (578.8, 649.4)
Bushehr	4332 (4230, 4451)	637.9 (619.8, 657.7)	5772 (5626, 5938)	899.5 (874.1, 928.8)	4311 (4130, 4528)	615.1 (583.2, 649.2)
Chahar Mahaal and Bakhtiari	4191 (4095, 4279)	587.9 (574, 602.5)	5742 (5605, 5877)	792.2 (771.6, 811.7)	4360 (4150, 4626)	562.9 (534.9, 599.6)
East Azarbayejan	22642 (22166, 23206)	709.3 (692.6, 727.2)	31564 (30848, 32394)	997.7 (975, 1025.2)	22675 (21539, 24052)	689.1 (651.5, 734.2)
Fars	21545 (21114, 22050)	544 (532.2, 555.9)	30569 (29924, 31320)	805.6 (787.4, 825.8)	19485 (18530, 20413)	500 (473.1, 525.5)
Gilan	16127 (15796, 16509)	605.3 (592.5, 619.3)	20692 (20242, 21260)	875.3 (856.8, 898.3)	13417 (12722, 14086)	557.4 (528.5, 588.5)
Golestan	8655 (8473, 8846)	599.6 (586.7, 614)	12361 (12073, 12675)	887.6 (868.1, 910.6)	8313 (7874, 8729)	568.3 (537.9, 593.2)
Hamadan	10069 (9843, 10322)	591.5 (578.4, 606.3)	14021 (13725, 14398)	853.9 (834.4, 877.1)	9258 (8808, 9786)	559.9 (533.1, 591.9)
Hormozgan	6534 (6386, 6670)	522.6 (510.6, 534.4)	8600 (8386, 8791)	719.7 (701.8, 737.2)	6625 (6348, 6931)	512.2 (487.6, 536.8)
Ilam	2515 (2453, 2577)	536.6 (522.2, 549.3)	3555 (3470, 3640)	787.8 (765.9, 808.3)	2629 (2485, 2775)	543.7 (514.4, 575.2)
Iran (Islamic Republic of)	378408 (372713, 384797)	582.5 (574, 592.4)	527380 (518847, 537262)	835.7 (822.4, 851.3)	365322 (352372, 378198)	550 (528.7, 571.4)
Isfahan	24144 (23666, 24686)	544.9 (533.5, 557.4)	33975 (33263, 34727)	791.4 (773.5, 810.6)	22858 (21784, 24188)	515.6 (488.8, 548.5)
Kerman	13234 (12965, 13503)	614.8 (601.5, 629.3)	18475 (18105, 18890)	879.2 (858, 901.6)	13054 (12438, 13650)	587.6 (554.6, 616)
Kermanshah	10766 (10547, 11001)	649.2 (635.8, 664.2)	15085 (14727, 15447)	914.3 (893.9, 935.9)	10436 (9887, 11005)	613.2 (582.3, 645.8)
Khorasan-e-Razavi	29966 (29390, 30581)	596.6 (585.1, 609.3)	41952 (41074, 42827)	854.1 (836.5, 871.7)	28011 (26741, 29335)	551.6 (525.2, 581.1)
Khuzestan	20929 (20509, 21392)	611.9 (599, 625.5)	27873 (27259, 28554)	829.7 (810.8, 848.2)	21281 (20347, 22306)	592.8 (565.3, 621.7)
Kohgiluyeh and Boyer-Ahmad	2830 (2769, 2908)	500.7 (488.1, 514.7)	3761 (3668, 3864)	704.6 (685.2, 725.5)	2774 (2639, 2924)	486.2 (460.8, 515.2)
Kurdistan	7911 (7740, 8087)	573.6 (560.6, 587.3)	11273 (11008, 11529)	782.4 (762.6, 800.7)	8843 (8404, 9260)	578.6 (550.2, 607.4)
Lorestan	8722 (8555, 8915)	665.5 (651.9, 682.1)	11760 (11512, 12022)	912.6 (891.6, 934.9)	8638 (8257, 9098)	637.2 (606.4, 674.5)
Markazi	7733 (7565, 7909)	571 (558.2, 584.6)	10870 (10622, 11120)	813.5 (794.1, 832.9)	7349 (6956, 7798)	533.1 (502.6, 565.3)
Mazandaran	16504 (16168, 16872)	571.3 (559, 584)	22920 (22456, 23481)	843 (824.2, 863.2)	15034 (14277, 15829)	526.9 (498.3, 557.1)
North Khorasan	4397 (4293, 4505)	660.7 (643.4, 678.7)	6047 (5891, 6216)	969.2 (943.7, 997)	4025 (3825, 4223)	633 (599.4, 667.5)
Qazvin	6072 (5925, 6241)	598.4 (583.3, 615.4)	8767 (8530, 9015)	864.6 (841.1, 890.5)	6212 (5868, 6537)	584.3 (552.5, 619.5)
Qom	6235 (6101, 6389)	934.6 (911.8, 957.6)	8182 (7995, 8405)	1288 (1253.2, 1323.3)	6217 (5890, 6509)	903 (849, 959.1)
Semnan	3490 (3408, 3590)	557.1 (543.2, 573.3)	4906 (4770, 5060)	836.3 (812.7, 862)	3338 (3142, 3511)	540.7 (507.6, 571.1)
Sistan and Baluchistan	12152 (11913, 12421)	649.7 (636.1, 667.3)	15415 (15094, 15774)	877.5 (858, 902.9)	11265 (10845, 11803)	588.7 (565.1, 617.1)
South Khorasan	3826 (3725, 3933)	583.8 (568.4, 600.6)	5096 (4958, 5237)	844.8 (822.3, 869)	3529 (3348, 3702)	559.3 (529.2, 590)
Tehran	57377 (56301, 58573)	477.2 (468.4, 486.5)	83151 (81594, 84973)	722.2 (708.3, 737.3)	53474 (50865, 55994)	436.5 (413.9, 460.1)
West Azarbayejan	16021 (15688, 16352)	690.5 (675.9, 706)	22041 (21611, 22551)	931.5 (911.8, 953.9)	16901 (15958, 17842)	674.9 (634.5, 712.9)
Yazd	5188 (5063, 5332)	562.5 (549.2, 578.7)	7362 (7167, 7578)	818.3 (795.8, 842.9)	4931 (4693, 5211)	536.9 (509.6, 569)
Zanjan	6544 (6388, 6730)	812 (792.5, 835.3)	9916 (9679, 10213)	1131.5 (1104, 1167.1)	7276 (6928, 7666)	802.6 (761.1, 848.9)

Table S2. All-cause mortality counts and age-standardised mortality rates per 100 000 across 31 provinces in females in 2019, 2020, and 2023

	2019		2020		2023	
**Province**	**Counts**	**Rates**	**Counts**	**Rates**	**Counts**	**Rates**
Alborz	4608 (4439, 4777)	501 (481.1, 521.7)	6831 (6574, 7085)	734.8 (704.9, 764.6)	5143 (4775, 5547)	495 (453.6, 537.3)
Ardebil	3015 (2913, 3123)	535.7 (516.7, 555)	4167 (4012, 4332)	757 (729.1, 787)	2981 (2757, 3202)	516.7 (476.4, 558.3)
Bushehr	1897 (1832, 1972)	585.9 (563.5, 611.2)	2561 (2468, 2658)	819.6 (787, 854.5)	1941 (1797, 2095)	565.8 (520.1, 614.6)
Chahar Mahaal and Bakhtiari	1837 (1777, 1914)	493.2 (475, 514.2)	2522 (2428, 2636)	659.4 (634.4, 690.1)	1977 (1834, 2151)	476.8 (440.9, 516.6)
East Azarbayejan	10128 (9802, 10469)	641.7 (621.8, 665.4)	14026 (13610, 14535)	885.2 (858.3, 920.1)	10351 (9525, 11236)	624.7 (576.2, 678.7)
Fars	9220 (8938, 9492)	458.2 (443.5, 471.9)	13012 (12603, 13432)	673 (650.6, 694.5)	8398 (7746, 9053)	421.4 (385.4, 458.3)
Gilan	7415 (7119, 7680)	543.1 (522.1, 561.9)	9420 (9055, 9787)	789.6 (759.1, 819.8)	6108 (5652, 6586)	504 (461.7, 544.7)
Golestan	3855 (3713, 3982)	514.8 (496.5, 533.6)	5576 (5377, 5769)	752.9 (724.5, 778.8)	3818 (3519, 4124)	487.2 (450.5, 528.1)
Hamadan	4439 (4282, 4602)	528.8 (510.8, 548.1)	6089 (5856, 6324)	749 (721.3, 776.6)	4227 (3905, 4617)	510.3 (469.5, 557.1)
Hormozgan	2650 (2568, 2740)	429.7 (413.7, 446.5)	3522 (3393, 3653)	582.7 (559.6, 607.2)	2689 (2523, 2869)	408.8 (376, 440.4)
Ilam	1045 (1003, 1083)	461.5 (442, 479.6)	1458 (1399, 1510)	664.9 (636.3, 692.1)	1093 (1015, 1174)	458.2 (424.4, 495.1)
Iran (Islamic Republic of)	164700 (160981, 168415)	511.7 (500.5, 522.8)	229026 (223149, 234877)	729.3 (711.9, 746.4)	159698 (150424, 167909)	482.3 (454.6, 507.1)
Isfahan	10520 (10158, 10879)	481.7 (464, 498.4)	14619 (14119, 15118)	690.8 (665.8, 717.1)	10050 (9266, 10870)	463 (426.8, 501.3)
Kerman	5645 (5468, 5846)	535.6 (517.8, 555.1)	8106 (7826, 8407)	767.9 (739.6, 796.7)	5881 (5481, 6311)	523.7 (481.9, 564.4)
Kermanshah	4524 (4375, 4676)	557.3 (538.9, 576.9)	6237 (6011, 6497)	761.6 (735.2, 791.7)	4500 (4176, 4920)	524.9 (484.6, 569.7)
Khorasan-e-Razavi	13161 (12821, 13520)	529.2 (514.1, 544.5)	18449 (17881, 18992)	758.5 (735.5, 782)	12164 (11323, 13102)	485.8 (451.5, 528.2)
Khuzestan	9062 (8697, 9354)	535.5 (512.7, 553.5)	11905 (11420, 12318)	716.2 (684.9, 741.2)	9201 (8561, 9871)	516.5 (479.3, 555.1)
Kohgiluyeh and Boyer-Ahmad	1221 (1174, 1264)	463.6 (444.5, 482.1)	1598 (1531, 1657)	623.1 (594.4, 648.1)	1241 (1152, 1326)	451.2 (415.4, 484.5)
Kurdistan	3426 (3296, 3560)	509.7 (490.4, 530.5)	4748 (4571, 4946)	674.7 (647.2, 701.8)	3729 (3436, 4032)	492.5 (452.5, 532.9)
Lorestan	3558 (3449, 3677)	531.6 (513.2, 550.3)	4902 (4738, 5082)	729.4 (701.9, 755.1)	3663 (3375, 3981)	508.7 (467.4, 554)
Markazi	3366 (3242, 3475)	468.8 (451.6, 484.5)	4778 (4604, 4948)	662.3 (638, 686.6)	3356 (3054, 3640)	445.9 (406, 484.4)
Mazandaran	7590 (7345, 7832)	513.3 (496, 530.8)	10529 (10202, 10932)	755.8 (729.5, 784.4)	7039 (6528, 7630)	478 (439.4, 520.8)
North Khorasan	2007 (1928, 2092)	625.8 (600.2, 652.7)	2736 (2627, 2863)	914.7 (876, 955)	1844 (1708, 1993)	605.6 (559.3, 659.2)
Qazvin	2610 (2505, 2717)	518.3 (496.5, 539.9)	3875 (3710, 4033)	753.8 (721.5, 786.4)	2751 (2538, 3017)	509.4 (471, 556.8)
Qom	2723 (2636, 2824)	818.7 (788.1, 852)	3482 (3367, 3609)	1096.5 (1054.8, 1139)	2769 (2550, 2987)	799.7 (725.9, 881.8)
Semnan	1559 (1506, 1623)	479.5 (463.4, 499.1)	2135 (2059, 2217)	724.6 (698.2, 754.8)	1429 (1315, 1552)	462.9 (423.2, 501.5)
Sistan and Baluchistan	5239 (5106, 5412)	567.2 (550.5, 589.6)	6534 (6336, 6755)	741.7 (717.6, 774.3)	4873 (4610, 5160)	517.2 (485.1, 553.8)
South Khorasan	1767 (1698, 1836)	522.8 (502.3, 543.3)	2289 (2201, 2381)	751.9 (722.7, 781.9)	1619 (1497, 1749)	505.2 (462.9, 550.3)
Tehran	24054 (23422, 24776)	415.7 (404.3, 428.7)	35258 (34266, 36402)	642 (625.3, 663.1)	21265 (19811, 22732)	370.9 (341, 398.5)
West Azarbayejan	7343 (7107, 7576)	621.4 (599.5, 641.3)	9995 (9650, 10346)	814.6 (785.6, 844.3)	8083 (7458, 8774)	618.7 (565, 670.6)
Yazd	2294 (2209, 2391)	493.7 (474.4, 514.3)	3265 (3134, 3403)	718.9 (689, 750.5)	2199 (2031, 2393)	477.7 (441.2, 520.6)
Zanjan	2924 (2817, 3028)	716.1 (689, 741.5)	4405 (4238, 4571)	990.8 (952.8, 1028.3)	3314 (3060, 3584)	712.3 (657.5, 771.8)

Table S3. All-cause mortality counts and age-standardised mortality rates per 100 000 across 31 provinces in males in 2019, 2020, and 2023

	2019		2020		2023	
**Province**	**Counts**	**Rates**	**Counts**	**Rates**	**Counts**	**Rates**
Alborz	6334 (6157, 6532)	597 (578.5, 616)	9386 (9099, 9665)	875.1 (847, 902.5)	6965 (6545, 7496)	587.3 (549.6, 632.8)
Ardebil	3802 (3688, 3914)	756.9 (732.8, 779.8)	5295 (5122, 5460)	1074.2 (1037.3, 1108.7)	3713 (3454, 3999)	735.6 (681, 795.5)
Bushehr	2435 (2352, 2518)	689.3 (664, 714.8)	3212 (3105, 3329)	983.2 (945.9, 1022.2)	2370 (2224, 2531)	667.9 (621.6, 718.2)
Chahar Mahaal and Bakhtiari	2353 (2278, 2437)	698.3 (673.2, 724.2)	3219 (3107, 3340)	950.2 (915.4, 988.3)	2383 (2218, 2573)	670.4 (617.5, 723.9)
East Azarbayejan	12514 (12159, 12888)	780 (757.2, 804.1)	17538 (17039, 18092)	1114.9 (1082.1, 1153.7)	12324 (11469, 13221)	756 (698.4, 809.2)
Fars	12325 (12000, 12683)	639.2 (621, 657.1)	17557 (17052, 18115)	951.3 (919, 983.7)	11087 (10462, 11737)	587.3 (548.5, 625.6)
Gilan	8712 (8477, 8963)	671.9 (653.2, 690.6)	11272 (10945, 11626)	965.9 (937.5, 993.2)	7309 (6847, 7757)	613 (574.2, 649.2)
Golestan	4799 (4644, 4958)	695.5 (670.9, 718.4)	6785 (6565, 7045)	1049.8 (1014.3, 1088.6)	4494 (4195, 4788)	667.5 (623.2, 709.5)
Hamadan	5630 (5458, 5807)	656 (635.5, 676.9)	7932 (7682, 8180)	961.7 (929.9, 994.5)	5032 (4727, 5384)	608.4 (570.7, 653.6)
Hormozgan	3884 (3771, 3993)	621 (602.1, 639.8)	5078 (4919, 5233)	871.3 (843.4, 902.1)	3935 (3722, 4165)	629.4 (591.7, 675.6)
Ilam	1470 (1423, 1519)	615.7 (593.9, 636.9)	2097 (2026, 2173)	911.7 (878.5, 945.4)	1537 (1438, 1639)	630.2 (593.1, 674.3)
Iran (Islamic Republic of)	213708 (208571, 219393)	656.4 (639.9, 672.9)	298353 (290734, 306696)	946.4 (921.9, 973.2)	205624 (196379, 215055)	620.5 (589.4, 653.3)
Isfahan	13624 (13239, 14053)	609.1 (591.9, 628.6)	19356 (18792, 19973)	893.4 (864.7, 921.1)	12808 (11993, 13654)	568.5 (530.6, 607.2)
Kerman	7589 (7384, 7828)	696.5 (676.9, 718.6)	10369 (10088, 10714)	997.2 (968.9, 1031.1)	7173 (6770, 7647)	654.6 (611.4, 700.4)
Kermanshah	6242 (6042, 6421)	745.6 (721.5, 767.2)	8848 (8557, 9132)	1073.3 (1037.8, 1108.2)	5936 (5527, 6360)	705.7 (660.8, 752.6)
Khorasan-e-Razavi	16805 (16387, 17322)	666.5 (648.5, 686.7)	23504 (22894, 24221)	952.5 (926.7, 982.8)	15847 (14890, 16847)	619.8 (582.4, 663.1)
Khuzestan	11867 (11531, 12200)	693.2 (671.9, 715.6)	15969 (15491, 16470)	949.2 (918.5, 981.1)	12080 (11339, 12791)	672.8 (627.1, 715.9)
Kohgiluyeh and Boyer-Ahmad	1610 (1563, 1669)	540.4 (523.8, 561.4)	2163 (2094, 2240)	786.4 (759.6, 818.4)	1534 (1431, 1622)	521.2 (485, 556.1)
Kurdistan	4485 (4350, 4633)	638.7 (619.3, 660.5)	6525 (6336, 6756)	891.5 (863, 923)	5114 (4789, 5424)	667.8 (624.4, 712.9)
Lorestan	5164 (5016, 5331)	819.5 (793.5, 847)	6858 (6661, 7080)	1123.9 (1087.8, 1164.4)	4974 (4647, 5255)	793 (738.6, 849.5)
Markazi	4367 (4232, 4502)	688 (664.8, 711.2)	6092 (5899, 6285)	997.3 (960.9, 1030.7)	3993 (3709, 4256)	641.2 (596.5, 686.8)
Mazandaran	8914 (8652, 9150)	633.7 (612.1, 653)	12391 (12005, 12740)	936.1 (904.3, 966.6)	7995 (7464, 8496)	579.1 (540.5, 624.3)
North Khorasan	2390 (2310, 2459)	700 (676.3, 722.7)	3311 (3200, 3420)	1026.8 (991.5, 1062.3)	2182 (2055, 2338)	662.1 (620.9, 712.6)
Qazvin	3461 (3349, 3585)	682.3 (660.8, 707)	4891 (4745, 5068)	984.4 (953.2, 1023)	3460 (3235, 3681)	666 (620.6, 713)
Qom	3512 (3406, 3642)	1067.9 (1026.1, 1110.4)	4700 (4536, 4900)	1511.9 (1450.7, 1574.6)	3448 (3224, 3663)	1028.9 (951.4, 1112.9)
Semnan	1931 (1869, 2009)	641.2 (619, 666.8)	2770 (2672, 2877)	952.7 (919.9, 990.8)	1909 (1772, 2034)	622.8 (577.1, 665.7)
Sistan and Baluchistan	6913 (6711, 7102)	731.2 (708.6, 752.4)	8881 (8614, 9155)	1011.7 (977.5, 1045.6)	6392 (6103, 6756)	660.5 (621, 703.2)
South Khorasan	2059 (1992, 2135)	649.6 (628.3, 673.3)	2807 (2718, 2912)	940 (906.5, 976.9)	1910 (1793, 2039)	615.5 (575.1, 659.9)
Tehran	33323 (32396, 34245)	540 (525.1, 555.2)	47893 (46543, 49348)	803.8 (782, 829)	32210 (29959, 34194)	503.6 (469.1, 538.1)
West Azarbayejan	8678 (8455, 8931)	765.4 (745.2, 789.7)	12045 (11730, 12430)	1063.4 (1031.6, 1096.5)	8818 (8270, 9492)	739 (692.3, 801.4)
Yazd	2894 (2800, 2991)	631.2 (607.9, 654)	4098 (3959, 4247)	918.5 (882.6, 951.9)	2732 (2550, 2927)	595.3 (551.9, 638.4)
Zanjan	3619 (3502, 3747)	917.5 (886.7, 951.4)	5510 (5332, 5731)	1281.7 (1238.6, 1330.9)	3962 (3716, 4256)	901.5 (845.3, 972.8)

Table S4. Age-standardised mortality rates for top 12 causes of death at national level in Iran in 2019, 2020, 2021, 2022, and 2023 in females.

2019			2020			2021			2022			2023			Percent change in rate
	Cause	Rate		Cause	Rate		Cause	Rate		Cause	Rate		Cause	Rate	2019 -2023
1	Ischemic heart disease	122 (96, 145.6)	1	COVID-19	250.4 (220.4, 291.5)	1	COVID-19	232.2 (204.4, 268)	1	Ischemic heart disease	104 (77, 128.5)	1	Ischemic heart disease	105.3 (78.5, 131.4)	-13.7%
2	Stroke	55.8 (42.9, 66.4)	2	Ischemic heart disease	118.3 (88.8, 145.2)	2	Ischemic heart disease	108.9 (80.5, 134)	2	COVID-19	54.3 (45.2, 68.9)	2	Stroke	45.1 (31.5, 63.6)	-19.1%
3	Alzheimer's disease	35.1 (8.7, 90.8)	3	Stroke	53.6 (39.5, 70.2)	3	Stroke	48.1 (34.4, 65.9)	3	Stroke	45.4 (31.9, 61.2)	3	Alzheimer’s disease	36 (9.3, 89.9)	2.7%
4	Hypertensive heart disease	34.2 (24.2, 46.2)	4	Hypertensive heart disease	34.9 (23.6, 48.2)	4	Hypertensive heart disease	32.8 (22.2, 44.5)	4	Alzheimer’s disease	36.1 (9.2, 90.8)	4	Hypertensive heart disease	32.1 (21.7, 46.5)	-6.2%
5	CKD	20.2 (13.3, 27.9)	5	Alzheimer's disease	29.3 (7.1, 79.8)	5	Alzheimer's disease	31.5 (7.7, 83.7)	5	Hypertensive heart disease	31.9 (21.7, 44.5)	5	CKD	20.2 (11.4, 29)	-0.1%
6	Diabetes mellitus	19.9 (11, 29.2)	6	CKD	20.6 (13.3, 29.1)	6	CKD	19.6 (12.3, 28.1)	6	CKD	19.5 (11.7, 28.5)	6	Diabetes mellitus	18.4 (10.6, 28.4)	-7.9%
7	LRI	17.3 (12.1, 25.5)	7	Diabetes mellitus	16.3 (9.2, 24.3)	7	Diabetes mellitus	17 (9.6, 26)	7	Diabetes mellitus	18.9 (10.8, 28.5)	7	COPD	17.6 (7.4, 27.5)	5.4%
8	COPD	16.6 (7.7, 25)	8	COPD	13 (5.4, 20.2)	8	COPD	14.1 (5.5, 22.1)	8	COPD	17.1 (7.5, 26.6)	8	LRI	16.8 (10.1, 27.3)	-3%
9	Road injuries	11.7 (7.5, 16.3)	9	LRI	12.8 (8.1, 18.6)	9	LRI	13.5 (8, 20.6)	9	LRI	16.5 (10.1, 26.4)	9	COVID-19	16.5 (13.2, 22.2)	-
10	Stomach cancer	11.4 (8.6, 15.4)	10	Road injuries	11.8 (7.5, 16.8)	10	Road injuries	11 (6.9, 15.4)	10	Stomach cancer	10 (7.1, 13.8)	10	Stomach cancer	9.9 (6.9, 14)	-12.9%
11	Breast cancer	10.5 (8.1, 12.1)	11	Stomach cancer	11.2 (8.3, 14.9)	11	Stomach cancer	10.4 (7.4, 14.1)	11	Road injuries	9.9 (6.4, 14.1)	11	Road injuries	9.8 (6.3, 14)	-15.8%
12	Neonatal disorders	9.6 (8.4, 10.7)	12	Breast cancer	10.3 (7.9, 12.1)	12	Breast cancer	9.4 (7.4, 11.1)	12	Breast cancer	9.1 (7.1, 11)	12	Breast cancer	9.2 (7.1, 11.2)	-12.7%

Table S5. Age-standardised mortality rates for top 12 causes of death at national level in Iran in 2019, 2020, 2021, 2022, and 2023 in Males.

2019			2020			2021			2022			2023			Percent change in rate
	Cause	Rate		Cause	Rate		Cause	Rate		Cause	Rate		Cause	Rate	2019 -2023
1	Ischemic heart disease	165.3 (139, 191.7)	1	COVID-19	323.8 (296.8, 355.4)	1	COVID-19	268.7 (243, 297.2)	1	Ischemic heart disease	148.8 (116.4, 181.2)	1	Ischemic heart disease	150.7 (118.3, 181.8)	-8.8%
2	Stroke	61.3 (50.5, 73.6)	2	Ischemic heart disease	163.9 (132.1, 198.9)	2	Ischemic heart disease	152.7 (119.4, 187.5)	2	COVID-19	60.2 (51.5, 69.6)	2	Stroke	49.3 (37, 65.7)	-19.6%
3	Road injuries	31.2 (21.4, 41.2)	3	Stroke	58.9 (46.1, 73.6)	3	Stroke	52.5 (39.4, 67.3)	3	Stroke	49.9 (38.3, 65.6)	3	COPD	26.7 (18.3, 38.8)	-2%
4	Hypertensive heart disease	27.9 (21, 37.4)	4	Road injuries	30.9 (20.6, 42)	4	Road injuries	28.5 (18.9, 39.3)	4	Road injuries	26.7 (17.9, 37.1)	4	Road injuries	26.5 (18.1, 36.9)	-15%
5	COPD	27.2 (20.3, 35.9)	5	Hypertensive heart disease	27.6 (20, 37.1)	5	Hypertensive heart disease	25 (17.5, 35)	5	COPD	26.6 (18.6, 37)	5	Alzheimer’s disease	25.2 (5.4, 66.4)	5.5%
6	Alzheimer’s disease	23.9 (5.2, 65)	6	CKD	21.8 (15.4, 29.1)	6	Alzheimer’s disease	22.2 (4.7, 61.4)	6	Alzheimer’s disease	25.2 (5.4, 67.9)	6	Hypertensive heart disease	23.7 (16.6, 33.7)	-15.3%
7	Diabetes mellitus	23.3 (18.4, 28.1)	7	COPD	20.8 (14.7, 27.8)	7	COPD	22.1 (14.9, 30.4)	7	Hypertensive heart disease	24 (16.8, 33.9)	7	Diabetes mellitus	22.5 (16.3, 31.6)	-3.3%
8	CKD	21.6 (15.8, 28.5)	8	Stomach cancer	20.7 (13.8, 25.7)	8	Diabetes mellitus	21.5 (17, 27.7)	8	Diabetes mellitus	23.2 (17.7, 31.8)	8	LRI	20.8 (15.4, 27.3)	-2.6%
9	LRI	21.3 (16.7, 26.1)	9	Alzheimer’s disease	20.5 (4.4, 58.7)	9	CKD	20.3 (14.2, 27.4)	9	LRI	20.3 (15.4, 25.9)	9	CKD	20.8 (13.1, 29.7)	-4%
10	Stomach cancer	20.6 (14.1, 25.4)	10	Diabetes mellitus	20.1 (16.2, 25)	10	Stomach cancer	19 (12.3, 24)	10	CKD	20.2 (13.4, 28.5)	10	Stomach cancer	18.5 (11.6, 25.3)	-10.4%
11	Lung cancer	17.3 (14.4, 21.7)	11	Lung cancer	17.5 (14.9, 22.3)	11	LRI	16.3 (12.4, 21.5)	11	Stomach cancer	18.6 (11.8, 24.4)	11	COVID-19	18 (14.7, 21.6)	-
12	Prostate cancer	14.4 (10.8, 18.3)	12	LRI	15.6 (12, 20.1)	12	Lung cancer	16.3 (13.9, 20.4)	12	Lung cancer	16.2 (13.2, 20.2)	12	Lung cancer	16.3 (13, 20.4)	-5.6%

Table S6· Counts of deaths for top 12 causes of death at national level in Iran in 2019, 2020, 2021, 2022, and 2023 in both sexes

2019			2020			2021			2022			2023		
	Cause	Count		Cause	Count		Cause	Count		Cause	Count		Cause	Count
1	Ischemic heart disease	88,650 (74,871-99,475)	1	COVID-19	182,401 (171,404-196,018)	1	COVID-19	168,502 (158,076-181,720)	1	Ischemic heart disease	77,058 (63,820-89,289)	1	Ischemic heart disease	80,939 (67,379-93,776)
2	Stroke	35,665 (30,237-41,448)	2	Ischemic heart disease	83,225 (69,634-95,705)	2	Ischemic heart disease	77,417 (63,947-90,218)	2	COVID-19	35,644 (32,080-40,128)	2	Stroke	29,144 (23,413-36,328)
3	Hypertensive heart disease	18,542 (14,542-23,502)	3	Stroke	32,549 (27,074-39,624)	3	Stroke	29,158 (23,573-35,947)	3	Stroke	28,377 (22,820-35,342)	3	Alzheimer's disease	16,625 (4,248-42,508)
4	Alzheimer’s disease	16,441 (4,128-42,781)	4	Hypertensive heart disease	17,562 (14,227-22,273)	4	Hypertensive heart disease	16,274 (12,841-20,668)	4	Alzheimer’s disease	15,997 (4,019-41,586)	4	Hypertensive heart disease	16,768 (12,874-21,257)
5	COPD	13,174 (9,809-16,541)	5	Alzheimer’s disease	12,584 (3,016-34,125)	5	Alzheimer’s disease	13,546 (3,290-36,258)	5	Hypertensive heart disease	16,180 (12,663-20,639)	5	COPD	13,593 (9,644-18,335)
6	Diabetes mellitus	14,027 (10,188-17,449)	6	Road injuries	17,680 (12,612-22,378)	6	CKD	12,128 (9,315-15,532)	6	COPD	12,879 (9,305-17,128)	6	CKD	13,283 (9,267-17,556)
7	Road injuries	17,691 (12,984-22,262)	7	CKD	12,815 (10,134-16,125)	7	Road injuries	16,423 (11,715-21,069)	7	Diabetes mellitus	14,085 (10,200-17,754)	7	Diabetes mellitus	14,243 (10,376-18,362)
8	CKD	13,127 (10,376-16,611)	8	Diabetes mellitus	11,594 (8,586-14,334)	8	Diabetes mellitus	12,519 (9,207-15,597)	8	CKD	12,419 (9,078-16,097)	8	LRI	11,592 (8,517-15,442)
9	LRI	11,887 (9,366-14,891)	9	COPD	9,509 (6,724-12,411)	9	COPD	10,228 (7,190-13,810)	9	Road injuries	15,331 (11,253-19,863)	9	Road injuries	15,660 (11,730-20,383)
10	Stomach cancer	10,767 (8,857-12,801)	10	Stomach cancer	10,622 (8,594-12,591)	10	LRI	8,630 (6,208-11,011)	10	LRI	10,986 (8,046-14,154)	10	COVID-19	10,029 (8,651-11,597)
11	lung cancer	8,635 (7,367-10,558)	11	LRI	8,264 (6,164-10,403)	11	Stomach cancer	9,913 (7,852-11,763)	11	Stomach cancer	9,984 (7,733-12,172)	11	Stomach cancer	10,315 (7,867-12,800)
12	Neonatal disorders	6,445 (5,972-7,002)	12	lung cancer	8,692 (7,487-10,548)	12	lung cancer	8,300 (7,150-10,042)	12	lung cancer	8,503 (7,199-10,319)	12	lung cancer	8,938 (7,467-10,962)

Table S7. Counts of deaths for top 12 causes of death at national level in Iran in 2019, 2020, 2021, 2022, and 2023 in females

2019			2020			2021			2022			2023		
	Cause	Count		Cause	Count		Cause	Count		Cause	Count		Cause	Count
1	Ischemic heart disease	37511 (29745, 44851)	1	COVID-19	80970 (72238, 92089)	1	COVID-19	79734 (71331, 89753)	1	Ischemic heart disease	31438 (23640, 38623)	1	Ischemic heart disease	33118 (24886, 41373)
2	Stroke	17277 (13377, 20561)	2	Ischemic heart disease	34356 (26198, 42333)	2	Ischemic heart disease	31895 (23804, 39201)	2	COVID-19	17654 (15002, 21312)	2	Stroke	14329 (10087, 20194)
3	Hypertensive heart disease	10397 (7356, 14129)	3	Stroke	15735 (11700, 20669)	3	Stroke	14260 (10276, 19544)	3	Stroke	13863 (9837, 18646)	3	Alzheimer's disease	10212 (2617, 26012)
4	Alzheimer's disease	10042 (2487, 26009)	4	Hypertensive heart disease	9966 (6728, 13757)	4	Hypertensive heart disease	9439 (6337, 12929)	4	Alzheimer's disease	9795 (2458, 25047)	4	Hypertensive heart disease	9919 (6681, 14327)
5	Diabetes mellitus	6524 (3593, 9504)	5	Alzheimer's disease	7641 (1832, 20806)	5	Alzheimer's disease	8241 (1992, 22059)	5	Hypertensive heart disease	9460 (6463, 13231)	5	CKD	6757 (3855, 9629)
6	CKD	6476 (4333, 8877)	6	CKD	6363 (4155, 8998)	6	CKD	6120 (3829, 8723)	6	Diabetes mellitus	6454 (3681, 9708)	6	Diabetes mellitus	6546 (3792, 10107)
7	LRI	5337 (3793, 7756)	7	Diabetes mellitus	5237 (2917, 7747)	7	Diabetes mellitus	5604 (3140, 8503)	7	CKD	6280 (3771, 9108)	7	COPD	5555 (2457, 8568)
8	COPD	5076 (2375, 7634)	8	Road injuries	4679 (2984, 6667)	8	Road injuries	4373 (2735, 6103)	8	COPD	5164 (2334, 7854)	8	LRI	5251 (3196, 8469)
9	Road injuries	4614 (2989, 6451)	9	Breast cancer	4255 (3177, 4972)	9	COPD	4062 (1651, 6350)	9	LRI	4970 (3082, 7767)	9	COVID-19	5058 (4135, 6575)
10	Breast cancer	4261 (3206, 4910)	10	Stomach cancer	3797 (2819, 5046)	10	Breast cancer	3990 (3016, 4656)	10	Road injuries	3996 (2574, 5642)	10	Breast cancer	4167 (3210, 5079)
11	Stomach cancer	3911 (2990, 5192)	11	LRI	3738 (2394, 5311)	11	LRI	3923 (2338, 5823)	11	Breast cancer	3973 (3083, 4752)	11	Road injuries	4051 (2627, 5726)
12	Cirrhosis and other chronic liver diseases	2811 (1844, 4573)	12	COPD	3727 (1564, 5722)	12	Stomach cancer	3561 (2590, 4811)	12	Stomach cancer	3547 (2558, 4964)	12	Stomach cancer	3679 (2574, 5228)

Table S8· Counts of deaths for top 12 causes of death at national level in Iran in 2019, 2020, 2021, 2022, and 2023 in males

2019			2020			2021			2022			2023		
	Cause	Count		Cause	Count		Cause	Count		Cause	Count		Cause	Count
1	Ischemic heart disease	51139 (43007, 59424)	1	COVID-19	101431 (93512, 109806)	1	COVID-19	88768 (80979, 96544)	1	Ischemic heart disease	45620 (36413, 55391)	1	Ischemic heart disease	47820 (38276, 57200)
2	Stroke	18388 (15280, 22026)	2	Ischemic heart disease	48869 (39595, 59106)	2	Ischemic heart disease	45521 (35833, 55791)	2	COVID-19	17990 (15591, 20328)	2	Stroke	14814 (11315, 19579)
3	Road injuries	13077 (9019, 17206)	3	Stroke	16814 (13314, 20990)	3	Stroke	14898 (11345, 19037)	3	Stroke	14514 (11272, 19086)	3	Road injuries	11609 (7914, 16035)
4	Hypertensive heart disease	8144 (6200, 10881)	4	Road injuries	13000 (8588, 17556)	4	Road injuries	12051 (8003, 16564)	4	Road injuries	11335 (7527, 15645)	4	COPD	8038 (5609, 11488)
5	COPD	8097 (6089, 10677)	5	Hypertensive heart disease	7597 (5481, 10251)	5	Diabetes mellitus	6915 (5477, 8848)	5	COPD	7716 (5412, 10707)	5	Diabetes mellitus	7697 (5566, 10736)
6	Diabetes mellitus	7503 (5954, 9111)	6	Stomach cancer	6825 (4625, 8533)	6	Hypertensive heart disease	6835 (4789, 9608)	6	Diabetes mellitus	7631 (5900, 10419)	6	Hypertensive heart disease	6849 (4792, 9792)
7	Stomach cancer	6857 (4724, 8463)	7	CKD	6452 (4607, 8623)	7	Stomach cancer	6352 (4193, 8032)	7	Hypertensive heart disease	6720 (4725, 9523)	7	Stomach cancer	6635 (4273, 9023)
8	CKD	6651 (4818, 8761)	8	Diabetes mellitus	6357 (5125, 7883)	8	COPD	6166 (4189, 8445)	8	Stomach cancer	6438 (4157, 8449)	8	CKD	6526 (4112, 9322)
9	LRI	6551 (5100, 8017)	9	Lung cancer	5996 (5116, 7739)	9	CKD	6007 (4250, 8104)	9	Alzheimer’s disease	6202 (1338, 16273)	9	Alzheimer’s disease	6413 (1380, 16483)
10	Alzheimer’s disease	6400 (1421, 17147)	10	COPD	5782 (4101, 7724)	10	Lung cancer	5714 (4862, 7348)	10	CKD	6139 (4105, 8642)	10	LRI	6341 (4698, 8296)
11	Lung cancer	5937 (4946, 7528)	11	Alzheimer’s disease	4943 (1088, 13817)	11	Alzheimer’s disease	5305 (1137, 14430)	11	LRI	6016 (4555, 7568)	11	Lung cancer	6163 (4954, 7768)
12	Cirrhosis and other chronic liver diseases	4224 (2907, 5928)	12	LRI	4526 (3496, 5813)	12	LRI	4708 (3581, 6126)	12	Lung cancer	5879 (4821, 7403)	12	COVID-19	4971 (4107, 5860)

Table S9. Life expectancy at birth across 31 provinces in both sexes combined in 1990, 2019, 2020, 2021, 2022, and 2023

	1990	2019	2020	2021	2022	2023
Alborz	73 (72.7, 73.3)	79.1 (78.9, 79.3)	75.5 (75.2, 75.7)	75.9 (75.7, 76.2)	78.9 (78.5, 79.2)	79.4 (78.9, 79.9)
Ardebil	68.5 (68.1, 68.9)	76.9 (76.7, 77.2)	73.2 (72.9, 73.5)	73.6 (73.3, 74)	76.8 (76.4, 77.2)	77.5 (76.9, 78)
Bushehr	69.3 (68.9, 69.6)	77.6 (77.3, 77.8)	74.3 (74, 74.6)	74.7 (74.4, 75)	77.6 (77.2, 78)	78.2 (77.7, 78.7)
Chahar Mahaal and Bakhtiari	68.6 (68.3, 69)	78 (77.8, 78.3)	75.2 (74.9, 75.5)	75.7 (75.3, 76)	78.2 (77.8, 78.5)	78.7 (78.2, 79.2)
East Azarbayejan	67.2 (66.9, 67.5)	76.5 (76.3, 76.7)	73.1 (72.9, 73.4)	73.6 (73.3, 73.9)	76.5 (76.2, 76.9)	77.1 (76.6, 77.6)
Fars	67.6 (67.3, 68)	78.8 (78.6, 79)	74.9 (74.6, 75.1)	75.6 (75.3, 75.8)	79.1 (78.8, 79.4)	80 (79.6, 80.5)
Gilan	44.8 (42.9, 46.5)	78.1 (77.9, 78.3)	74.6 (74.4, 74.9)	75.3 (75, 75.6)	78.5 (78.1, 78.8)	79.3 (78.9, 79.8)
Golestan	68 (67.6, 68.4)	77.1 (76.9, 77.4)	72.5 (72.2, 72.8)	73.2 (72.8, 73.5)	77 (76.6, 77.5)	77.9 (77.4, 78.5)
Hamadan	66.5 (66.2, 66.9)	77.4 (77.1, 77.7)	73.3 (73, 73.6)	73.9 (73.6, 74.2)	77.5 (77.1, 77.8)	78.3 (77.7, 78.8)
Hormozgan	67.6 (67.2, 68)	78.6 (78.4, 78.9)	74.9 (74.6, 75.2)	75.4 (75.1, 75.8)	78.3 (77.9, 78.7)	79 (78.4, 79.5)
Ilam	69.3 (69, 69.7)	78.7 (78.5, 79)	74.7 (74.4, 75)	75.1 (74.8, 75.4)	78.1 (77.7, 78.5)	78.7 (78.2, 79.3)
Iran (Islamic Republic of)	66.4 (66.1, 66.7)	77.9 (77.8, 78.1)	74.2 (74, 74.4)	74.8 (74.6, 75.1)	78.1 (77.8, 78.3)	78.8 (78.5, 79.2)
Isfahan	69.7 (69.4, 70.1)	79.1 (78.9, 79.4)	75.6 (75.4, 75.9)	76.2 (76, 76.5)	79.3 (78.9, 79.6)	80 (79.5, 80.5)
Kerman	67.9 (67.6, 68.3)	77.5 (77.2, 77.7)	73.7 (73.5, 74)	74.3 (74, 74.5)	77.5 (77.2, 77.8)	78.2 (77.7, 78.7)
Kermanshah	64.8 (64.5, 65.2)	76.3 (76.1, 76.6)	72.6 (72.3, 72.9)	73.1 (72.8, 73.5)	76.4 (76.1, 76.8)	77.3 (76.7, 77.8)
Khorasan-e-Razavi	65.9 (65.6, 66.3)	77.5 (77.3, 77.8)	73.7 (73.4, 73.9)	74.4 (74.1, 74.7)	77.9 (77.5, 78.2)	78.7 (78.2, 79.2)
Khuzestan	68.4 (68, 68.7)	76.7 (76.4, 77)	73.2 (72.9, 73.5)	73.6 (73.2, 73.9)	76.6 (76.2, 77)	77.2 (76.7, 77.8)
Kohgiluyeh and Boyer-Ahmad	68.7 (68.3, 69.1)	79.1 (78.8, 79.4)	75.3 (74.9, 75.6)	75.8 (75.4, 76.1)	78.9 (78.5, 79.4)	79.6 (79, 80.2)
Kurdistan	66.8 (66.4, 67.2)	77.8 (77.5, 78)	74.3 (74, 74.6)	74.8 (74.5, 75.1)	77.2 (76.8, 77.6)	77.8 (77.2, 78.4)
Lorestan	67.5 (67.1, 67.8)	76.6 (76.3, 76.8)	73.4 (73.1, 73.6)	73.8 (73.5, 74.1)	76.7 (76.3, 77)	77.2 (76.7, 77.7)
Markazi	68.5 (68.1, 68.8)	78.4 (78.1, 78.6)	74.8 (74.5, 75.1)	75.4 (75.2, 75.7)	78.6 (78.2, 78.9)	79.4 (78.9, 79.9)
Mazandaran	69.6 (69.3, 69.9)	78.9 (78.6, 79.1)	75.3 (75.1, 75.5)	76 (75.7, 76.2)	79.2 (78.8, 79.5)	80.1 (79.6, 80.5)
North Khorasan	66.1 (65.8, 66.5)	76.3 (76, 76.5)	72 (71.7, 72.3)	72.5 (72.2, 72.9)	76.2 (75.8, 76.6)	76.9 (76.3, 77.5)
Qazvin	69.7 (69.4, 70)	77.8 (77.5, 78)	74 (73.7, 74.2)	74.4 (74.1, 74.8)	77.6 (77.2, 77.9)	78.2 (77.6, 78.7)
Qom	68.1 (67.8, 68.4)	75.7 (75.5, 76)	73 (72.7, 73.2)	73.4 (73.1, 73.6)	75.9 (75.5, 76.2)	76.4 (76, 76.8)
Semnan	70.3 (70, 70.7)	78.6 (78.3, 78.9)	74.5 (74.2, 74.9)	75.1 (74.8, 75.5)	78.4 (78, 78.8)	79.1 (78.6, 79.7)
Sistan and Baluchistan	60.4 (60, 60.8)	74.9 (74.5, 75.2)	70.6 (70.3, 71)	71.3 (70.9, 71.6)	75.4 (75, 75.9)	76.5 (75.9, 77.2)
South Khorasan	68.2 (67.8, 68.5)	78.5 (78.2, 78.7)	75 (74.7, 75.3)	75.5 (75.2, 75.8)	78.5 (78.2, 78.9)	79.2 (78.7, 79.7)
Tehran	71.6 (71.3, 71.9)	80.3 (80.1, 80.5)	76.1 (75.9, 76.3)	76.9 (76.7, 77.2)	80.5 (80.2, 80.8)	81.5 (81, 81.9)
West Azarbayejan	67.4 (67, 67.7)	76.4 (76.1, 76.6)	73.3 (73.1, 73.6)	73.7 (73.4, 74)	76.3 (76, 76.7)	76.8 (76.3, 77.4)
Yazd	69.2 (68.9, 69.6)	78.5 (78.2, 78.8)	74.8 (74.5, 75.1)	75.3 (75, 75.7)	78.5 (78.2, 78.9)	79.2 (78.7, 79.8)
Zanjan	42.8 (41.2, 44.2)	74.4 (74.2, 74.7)	71.1 (70.8, 71.4)	71.5 (71.1, 71.8)	74.3 (73.9, 74.7)	74.8 (74.3, 75.4)

Table S10. Life expectancy at birth across 31 provinces in females in 1990, 2019, 2020, 2021, 2022, and 2023

	1990	2019	2020	2021	2022	2023
Alborz	75.6 (75.1, 76)	80.6 (80.3, 81)	77.3 (77, 77.7)	77.5 (77.1, 78)	80.3 (79.9, 80.8)	80.9 (80.2, 81.5)
Ardebil	69.9 (69.4, 70.4)	78.9 (78.4, 79.3)	75.1 (74.6, 75.5)	75.3 (74.8, 75.8)	78.7 (78.1, 79.3)	79.4 (78.6, 80.3)
Bushehr	70.5 (70, 71)	78.8 (78.4, 79.2)	75.7 (75.2, 76.1)	75.9 (75.4, 76.3)	78.8 (78.3, 79.4)	79.4 (78.7, 80.1)
Chahar Mahaal and Bakhtiari	70.3 (69.7, 70.8)	80 (79.6, 80.4)	77.3 (76.9, 77.7)	77.5 (77, 78)	80 (79.5, 80.6)	80.6 (79.8, 81.3)
East Azarbayejan	67.9 (67.4, 68.4)	77.9 (77.6, 78.2)	74.8 (74.5, 75.2)	75 (74.6, 75.4)	77.9 (77.4, 78.4)	78.5 (77.8, 79.2)
Fars	70.2 (69.7, 70.6)	81 (80.6, 81.3)	77.3 (76.9, 77.6)	77.7 (77.3, 78.1)	81.2 (80.7, 81.6)	82.1 (81.4, 82.7)
Gilan	42.3 (40.2, 44.2)	79.7 (79.4, 80.1)	76.4 (76.1, 76.8)	76.8 (76.4, 77.3)	80 (79.5, 80.5)	80.8 (80.1, 81.5)
Golestan	71.5 (70.9, 72)	79.2 (78.8, 79.7)	74.7 (74.3, 75.2)	75 (74.5, 75.5)	79.1 (78.5, 79.7)	80 (79.1, 80.9)
Hamadan	70 (69.5, 70.5)	79.3 (78.9, 79.6)	75.7 (75.3, 76.1)	76 (75.5, 76.4)	79.2 (78.6, 79.7)	79.8 (79, 80.6)
Hormozgan	71.4 (70.8, 71.9)	81.2 (80.8, 81.7)	77.8 (77.3, 78.2)	78 (77.5, 78.6)	81.2 (80.5, 81.8)	81.9 (81, 82.7)
Ilam	71.1 (70.5, 71.6)	80.6 (80.2, 81)	76.8 (76.4, 77.3)	77 (76.5, 77.5)	80.2 (79.6, 80.7)	80.8 (80.1, 81.6)
Iran (Islamic Republic of)	67.8 (67.4, 68.2)	79.7 (79.5, 80)	76.2 (75.9, 76.5)	76.6 (76.2, 76.9)	79.8 (79.4, 80.2)	80.6 (80.1, 81.1)
Isfahan	72.1 (71.6, 72.6)	80.8 (80.6, 81.2)	77.6 (77.3, 78)	78 (77.6, 78.3)	80.8 (80.3, 81.3)	81.5 (80.8, 82.2)
Kerman	71.2 (70.7, 71.6)	79.4 (79, 79.7)	75.8 (75.4, 76.2)	75.9 (75.5, 76.4)	79.1 (78.6, 79.7)	79.8 (79.1, 80.5)
Kermanshah	68.1 (67.6, 68.6)	78.5 (78.1, 78.9)	75.3 (74.9, 75.7)	75.6 (75.1, 76)	78.7 (78.1, 79.2)	79.4 (78.6, 80.2)
Khorasan-e-Razavi	67.1 (66.6, 67.6)	79.2 (78.9, 79.6)	75.5 (75.1, 75.8)	75.9 (75.5, 76.3)	79.5 (79, 80)	80.4 (79.7, 81.2)
Khuzestan	70.3 (69.8, 70.8)	78.7 (78.3, 79.1)	75.4 (75, 75.8)	75.6 (75.1, 76)	78.5 (78, 79.1)	79.3 (78.5, 80.1)
Kohgiluyeh and Boyer-Ahmad	70.1 (69.5, 70.6)	80.3 (79.8, 80.7)	77 (76.5, 77.5)	77.2 (76.7, 77.8)	80.1 (79.5, 80.7)	80.7 (79.9, 81.6)
Kurdistan	68.3 (67.7, 68.8)	79.4 (79, 79.8)	76.4 (76, 76.8)	76.6 (76.1, 77.1)	79.3 (78.7, 80)	79.9 (79.1, 80.8)
Lorestan	69.9 (69.3, 70.4)	79.2 (78.9, 79.6)	76 (75.7, 76.4)	76.3 (75.9, 76.8)	79.2 (78.7, 79.8)	79.9 (79.1, 80.6)
Markazi	71 (70.5, 71.5)	80.7 (80.3, 81)	77.2 (76.9, 77.6)	77.6 (77.2, 78)	80.6 (80.1, 81.2)	81.4 (80.6, 82.1)
Mazandaran	72 (71.5, 72.5)	80.3 (79.9, 80.6)	76.8 (76.4, 77.1)	77.2 (76.8, 77.6)	80.4 (79.9, 80.9)	81.2 (80.6, 81.9)
North Khorasan	68.3 (67.8, 68.8)	77.4 (77, 77.9)	73.4 (72.9, 73.9)	73.6 (73.1, 74.1)	77.2 (76.6, 77.8)	78 (77.1, 78.8)
Qazvin	71.5 (71, 72)	79.6 (79.2, 80)	75.9 (75.5, 76.3)	76.1 (75.6, 76.6)	79.3 (78.8, 79.9)	80 (79.1, 80.7)
Qom	71 (70.5, 71.4)	77.4 (77.1, 77.7)	75 (74.6, 75.4)	75.1 (74.7, 75.6)	77.4 (76.9, 77.9)	77.9 (77.2, 78.5)
Semnan	71.9 (71.4, 72.5)	80.4 (80, 80.9)	76.4 (75.9, 76.8)	76.7 (76.2, 77.2)	80.2 (79.6, 80.8)	81 (80.2, 81.8)
Sistan and Baluchistan	62.5 (61.8, 63.1)	77 (76.5, 77.5)	73.1 (72.6, 73.6)	73.4 (72.7, 73.9)	77.4 (76.8, 78.1)	78.5 (77.6, 79.4)
South Khorasan	70 (69.5, 70.5)	79.9 (79.6, 80.3)	76.5 (76.1, 76.9)	76.7 (76.3, 77.2)	79.8 (79.3, 80.4)	80.5 (79.8, 81.2)
Tehran	72.7 (72.2, 73.2)	82.2 (81.9, 82.4)	78.2 (77.9, 78.4)	78.8 (78.4, 79.1)	82.5 (82.1, 82.9)	83.5 (82.9, 84.2)
West Azarbayejan	68.7 (68.1, 69.2)	77.7 (77.3, 78)	74.9 (74.6, 75.3)	75 (74.6, 75.5)	77.5 (76.9, 78.1)	77.9 (77.1, 78.7)
Yazd	71.9 (71.3, 72.4)	80.3 (79.9, 80.7)	76.7 (76.3, 77.1)	77 (76.5, 77.4)	80.1 (79.6, 80.7)	80.8 (80.1, 81.6)
Zanjan	38.9 (37.1, 40.6)	76.2 (75.7, 76.5)	72.9 (72.5, 73.3)	73.1 (72.6, 73.5)	76 (75.4, 76.5)	76.5 (75.7, 77.2)

Table S11. Life expectancy at birth across 31 provinces in males in 1990, 2019, 2020, 2021, 2022, and 2023

	1990	2019	2020	2021	2022	2023
Alborz	71 (70.6, 71.4)	77.6 (77.3, 78)	73.8 (73.4, 74.1)	74.4 (74, 74.8)	77.4 (76.9, 77.9)	78 (77.3, 78.7)
Ardebil	67.5 (67.1, 68)	75.1 (74.8, 75.5)	71.5 (71.1, 71.9)	72.1 (71.7, 72.6)	75.1 (74.5, 75.6)	75.6 (74.9, 76.4)
Bushehr	68.1 (67.7, 68.6)	76.5 (76.2, 76.8)	73.1 (72.7, 73.5)	73.7 (73.3, 74.1)	76.5 (76, 77)	77.1 (76.4, 77.8)
Chahar Mahaal and Bakhtiari	67.4 (66.9, 67.8)	76.2 (75.8, 76.6)	73.4 (73, 73.8)	74 (73.5, 74.4)	76.4 (75.9, 76.9)	77 (76.2, 77.7)
East Azarbayejan	66.7 (66.2, 67.1)	75.2 (74.9, 75.5)	71.6 (71.2, 71.9)	72.2 (71.9, 72.6)	75.2 (74.8, 75.7)	75.8 (75.2, 76.5)
Fars	65.4 (65, 65.9)	76.8 (76.5, 77)	72.7 (72.4, 73)	73.6 (73.2, 74)	77.2 (76.7, 77.6)	78.1 (77.5, 78.8)
Gilan	47.5 (45.8, 48.9)	76.5 (76.2, 76.8)	73 (72.6, 73.3)	73.8 (73.5, 74.2)	77 (76.5, 77.5)	78 (77.4, 78.7)
Golestan	65.2 (64.7, 65.6)	75.2 (74.8, 75.5)	70.5 (70.1, 70.9)	71.4 (70.9, 71.8)	75 (74.4, 75.5)	75.9 (75.2, 76.7)
Hamadan	63.7 (63.3, 64.2)	75.6 (75.2, 76)	71.1 (70.7, 71.5)	72 (71.6, 72.5)	75.8 (75.3, 76.4)	76.8 (76, 77.6)
Hormozgan	64.7 (64.2, 65.2)	76.3 (75.9, 76.7)	72.4 (72, 72.8)	73.1 (72.6, 73.5)	75.6 (75.1, 76.1)	76.3 (75.6, 77.1)
Ilam	68.1 (67.7, 68.6)	77 (76.6, 77.4)	72.8 (72.4, 73.2)	73.4 (72.9, 73.9)	76.3 (75.7, 76.8)	76.9 (76.1, 77.6)
Iran (Islamic Republic of)	65.2 (64.9, 65.5)	76.3 (76, 76.5)	72.4 (72.1, 72.7)	73.2 (72.9, 73.5)	76.4 (76, 76.8)	77.2 (76.7, 77.7)
Isfahan	67.7 (67.3, 68.2)	77.6 (77.3, 77.9)	73.8 (73.5, 74.2)	74.7 (74.3, 75)	77.9 (77.4, 78.3)	78.7 (78, 79.3)
Kerman	65.2 (64.8, 65.7)	75.7 (75.3, 76)	71.9 (71.5, 72.3)	72.7 (72.3, 73.1)	76 (75.5, 76.4)	76.8 (76.1, 77.4)
Kermanshah	62.3 (61.8, 62.8)	74.2 (73.9, 74.6)	70.1 (69.7, 70.5)	70.8 (70.4, 71.3)	74.3 (73.8, 74.9)	75.2 (74.5, 76)
Khorasan-e-Razavi	64.8 (64.4, 65.3)	75.9 (75.6, 76.2)	72 (71.7, 72.4)	72.9 (72.6, 73.3)	76.3 (75.8, 76.8)	77.1 (76.5, 77.8)
Khuzestan	66.6 (66.1, 67.1)	74.8 (74.5, 75.2)	71.2 (70.8, 71.5)	71.7 (71.3, 72.2)	74.8 (74.2, 75.3)	75.4 (74.6, 76.2)
Kohgiluyeh and Boyer-Ahmad	67.6 (67.1, 68.1)	78 (77.6, 78.4)	73.7 (73.2, 74.1)	74.4 (73.9, 74.9)	77.9 (77.3, 78.5)	78.6 (77.9, 79.4)
Kurdistan	65.8 (65.3, 66.3)	76.2 (75.8, 76.6)	72.5 (72, 72.8)	73.1 (72.6, 73.5)	75.3 (74.7, 75.8)	75.8 (75.1, 76.6)
Lorestan	65.5 (65.1, 66)	74.1 (73.8, 74.5)	70.9 (70.5, 71.3)	71.5 (71.1, 71.9)	74.3 (73.7, 74.8)	74.8 (74, 75.5)
Markazi	66.3 (65.8, 66.7)	76.3 (75.9, 76.6)	72.6 (72.2, 72.9)	73.4 (73, 73.8)	76.6 (76.1, 77.1)	77.4 (76.8, 78.1)
Mazandaran	67.3 (66.9, 67.8)	77.5 (77.2, 77.8)	73.9 (73.6, 74.2)	74.8 (74.4, 75.1)	78 (77.5, 78.4)	79 (78.3, 79.6)
North Khorasan	64.3 (63.8, 64.8)	75.1 (74.7, 75.5)	70.7 (70.3, 71.1)	71.5 (71, 72)	75.1 (74.6, 75.7)	75.9 (75.1, 76.7)
Qazvin	68.1 (67.7, 68.6)	76 (75.7, 76.4)	72.2 (71.9, 72.6)	72.9 (72.5, 73.4)	75.9 (75.4, 76.4)	76.5 (75.8, 77.2)
Qom	65.7 (65.3, 66.1)	74.2 (73.9, 74.5)	71.1 (70.8, 71.5)	71.7 (71.3, 72.1)	74.4 (74, 74.9)	75 (74.4, 75.6)
Semnan	68.9 (68.4, 69.3)	76.9 (76.5, 77.2)	72.9 (72.5, 73.3)	73.7 (73.2, 74.1)	76.8 (76.2, 77.3)	77.4 (76.7, 78.1)
Sistan and Baluchistan	58.8 (58.3, 59.4)	73 (72.6, 73.4)	68.5 (68.1, 69)	69.4 (68.9, 69.9)	73.7 (73, 74.2)	74.8 (74, 75.6)
South Khorasan	66.6 (66.1, 67)	77.1 (76.7, 77.4)	73.5 (73.1, 73.9)	74.3 (73.8, 74.7)	77.3 (76.8, 77.8)	78 (77.3, 78.7)
Tehran	70.6 (70.1, 71)	78.5 (78.2, 78.8)	74.2 (73.9, 74.5)	75.2 (74.9, 75.6)	78.6 (78.2, 79.1)	79.5 (78.9, 80.2)
West Azarbayejan	66.6 (66.1, 67)	75.2 (74.8, 75.5)	71.8 (71.5, 72.2)	72.5 (72, 72.9)	75.2 (74.7, 75.7)	75.8 (75.1, 76.5)
Yazd	66.9 (66.5, 67.4)	76.9 (76.5, 77.2)	73.1 (72.7, 73.5)	73.9 (73.4, 74.4)	77.1 (76.6, 77.7)	77.8 (77.1, 78.6)
Zanjan	47.1 (45.8, 48.3)	72.8 (72.4, 73.2)	69.4 (69, 69.8)	69.9 (69.5, 70.4)	72.7 (72.2, 73.3)	73.2 (72.5, 74)

Table S12. COVID-19 mortality counts across 31 provinces in both sexes combined in 2020, 2021, 2022, and 2023

	COVID-19 mortality counts			
	**2020**	**2021**	**2022**	**2023**
Alborz	6070 (5244, 7180)	6033 (5269, 6976)	1334 (1066, 1702)	376 (283, 495)
Ardebil	3122 (2600, 3671)	2805 (2359, 3289)	579 (451, 721)	162 (117, 210)
Bushehr	1986 (1701, 2338)	1918 (1633, 2242)	392 (299, 487)	103 (79, 131)
Chahar Mahaal and Bakhtiari	1834 (1522, 2160)	1625 (1347, 1928)	354 (273, 447)	103 (75, 141)
East Azarbayejan	9990 (8453, 11813)	9202 (7749, 10877)	1860 (1481, 2364)	511 (381, 675)
Fars	12030 (10486, 13662)	10646 (9221, 12016)	2360 (1894, 2894)	681 (526, 868)
Gilan	7748 (6419, 8905)	7178 (6086, 8264)	1568 (1240, 1943)	444 (332, 582)
Golestan	4574 (3887, 5383)	4436 (3854, 5213)	907 (725, 1169)	241 (185, 324)
Hamadan	4831 (4098, 5538)	4184 (3535, 4876)	880 (683, 1123)	248 (185, 332)
Hormozgan	2939 (2545, 3375)	2689 (2275, 3080)	588 (467, 738)	168 (129, 213)
Ilam	1375 (1152, 1585)	1269 (1054, 1465)	289 (220, 360)	85 (62, 111)
Iran (Islamic Republic of)	182401 (171404, 196018)	168502 (158076, 181720)	35644 (32080, 40128)	10029 (8651, 11597)
Isfahan	12601 (10656, 14319)	11579 (9759, 13159)	2560 (2001, 3194)	732 (567, 941)
Kerman	6364 (5505, 7380)	6005 (5199, 6952)	1268 (1023, 1588)	353 (273, 463)
Kermanshah	4949 (4187, 5766)	4411 (3643, 5155)	898 (693, 1125)	246 (186, 323)
Khorasan-e-Razavi	14401 (12316, 16618)	13434 (11652, 15519)	2802 (2219, 3446)	774 (592, 1033)
Khuzestan	8917 (7661, 10466)	8318 (6972, 9642)	1661 (1316, 2078)	446 (342, 585)
Kohgiluyeh and Boyer-Ahmad	1295 (1115, 1474)	1212 (1040, 1399)	270 (210, 341)	79 (57, 108)
Kurdistan	3616 (3092, 4178)	3322 (2810, 3871)	722 (559, 904)	209 (157, 269)
Lorestan	3681 (3133, 4367)	3310 (2763, 3950)	685 (532, 870)	191 (142, 258)
Markazi	3654 (3109, 4237)	3379 (2850, 3905)	740 (575, 920)	215 (157, 278)
Mazandaran	8669 (7348, 10220)	7832 (6635, 9084)	1686 (1317, 2103)	475 (358, 652)
North Khorasan	2003 (1692, 2348)	1905 (1616, 2250)	376 (302, 465)	99 (76, 130)
Qazvin	3046 (2601, 3541)	2778 (2354, 3213)	589 (458, 729)	167 (123, 216)
Qom	2131 (1779, 2665)	2104 (1771, 2584)	393 (307, 535)	101 (75, 143)
Semnan	1841 (1571, 2124)	1681 (1437, 1943)	369 (294, 454)	106 (81, 137)
Sistan and Baluchistan	3846 (3293, 4404)	3727 (3201, 4311)	700 (564, 845)	184 (146, 232)
South Khorasan	1749 (1499, 2035)	1585 (1361, 1838)	345 (268, 428)	98 (73, 129)
Tehran	31158 (27234, 34722)	29062 (25412, 32333)	6252 (5066, 7483)	1811 (1391, 2266)
West Azarbayejan	6520 (5500, 7794)	5849 (4865, 6946)	1151 (918, 1501)	315 (239, 431)
Yazd	2702 (2313, 3124)	2408 (2047, 2795)	537 (423, 670)	159 (122, 207)
Zanjan	2762 (2262, 3268)	2617 (2173, 3069)	527 (405, 661)	145 (108, 195)

Table S13. COVID-19 mortality counts across 31 provinces in females in 2020, 2021, 2022, and 2023

	COVID-19 mortality counts			
	2020	2021	2022	2023
Alborz	2718 (2232, 3426)	2879 (2406, 3510)	686 (528, 932)	197 (137, 309)
Ardebil	1388 (1145, 1668)	1338 (1097, 1604)	289 (220, 370)	82 (59, 113)
Bushehr	859 (714, 1019)	888 (725, 1044)	187 (141, 244)	50 (36, 70)
Chahar Mahaal and Bakhtiari	820 (658, 989)	776 (613, 946)	180 (130, 240)	54 (38, 77)
East Azarbayejan	4306 (3523, 5342)	4260 (3580, 5214)	890 (685, 1222)	248 (179, 362)
Fars	5400 (4585, 6261)	5057 (4326, 5907)	1191 (939, 1532)	354 (255, 483)
Gilan	3730 (3062, 4547)	3633 (3028, 4378)	841 (646, 1126)	244 (170, 361)
Golestan	2111 (1770, 2577)	2206 (1834, 2651)	467 (358, 626)	126 (92, 175)
Hamadan	2068 (1703, 2469)	1897 (1568, 2260)	415 (314, 533)	119 (83, 165)
Hormozgan	1289 (1067, 1501)	1254 (1035, 1447)	294 (227, 368)	88 (63, 118)
Ilam	584 (481, 678)	573 (469, 667)	132 (99, 167)	39 (28, 53)
Iran (Islamic Republic of)	80970 (72238, 92089)	79734 (71331, 89753)	17654 (15002, 21312)	5058 (4135, 6575)
Isfahan	5448 (4595, 6624)	5315 (4439, 6462)	1234 (938, 1649)	360 (270, 512)
Kerman	2924 (2509, 3499)	2945 (2502, 3530)	665 (524, 865)	190 (139, 266)
Kermanshah	2091 (1737, 2464)	1999 (1653, 2361)	424 (316, 547)	117 (84, 159)
Khorasan-e-Razavi	6391 (5345, 7540)	6420 (5566, 7443)	1391 (1087, 1771)	389 (287, 527)
Khuzestan	3898 (3291, 4635)	3899 (3213, 4620)	817 (641, 1066)	224 (167, 300)
Kohgiluyeh and Boyer-Ahmad	523 (432, 623)	524 (430, 622)	119 (90, 155)	35 (24, 49)
Kurdistan	1466 (1213, 1759)	1455 (1162, 1748)	320 (232, 415)	94 (65, 128)
Lorestan	1654 (1365, 2002)	1591 (1307, 1920)	350 (255, 461)	100 (70, 145)
Markazi	1654 (1382, 1966)	1628 (1363, 1895)	381 (292, 485)	115 (84, 153)
Mazandaran	4134 (3441, 5095)	3974 (3297, 4824)	895 (658, 1206)	255 (185, 361)
North Khorasan	905 (747, 1080)	930 (775, 1113)	187 (144, 238)	49 (36, 63)
Qazvin	1359 (1097, 1614)	1315 (1081, 1551)	292 (220, 375)	85 (61, 116)
Qom	892 (711, 1250)	947 (769, 1274)	188 (141, 289)	50 (35, 85)
Semnan	819 (684, 981)	791 (664, 933)	181 (138, 236)	53 (38, 73)
Sistan and Baluchistan	1479 (1221, 1721)	1574 (1301, 1799)	294 (229, 367)	77 (56, 100)
South Khorasan	783 (655, 952)	766 (639, 910)	171 (128, 220)	49 (36, 65)
Tehran	14045 (12176, 16066)	13823 (11875, 15628)	3097 (2433, 3826)	909 (667, 1229)
West Azarbayejan	2802 (2244, 3532)	2679 (2147, 3283)	543 (413, 740)	152 (105, 238)
Yazd	1223 (1019, 1486)	1156 (972, 1368)	274 (209, 363)	85 (60, 118)
Zanjan	1209 (967, 1446)	1242 (1016, 1476)	258 (195, 329)	72 (51, 96)

Table S14. COVID-19 mortality counts across 31 provinces in males in 2020, 2021, 2022, and 2023

	COVID-19 mortality counts			
	2020	2021	2022	2023
Alborz	3351 (2861, 3904)	3154 (2696, 3649)	648 (505, 820)	179 (131, 227)
Ardebil	1734 (1428, 2095)	1467 (1218, 1795)	291 (221, 373)	80 (56, 110)
Bushehr	1127 (967, 1320)	1030 (871, 1215)	205 (156, 260)	53 (38, 70)
Chahar Mahaal and Bakhtiari	1014 (834, 1205)	849 (692, 1027)	175 (129, 229)	49 (35, 67)
East Azarbayejan	5684 (4770, 6757)	4942 (4105, 5926)	970 (741, 1247)	263 (191, 364)
Fars	6630 (5673, 7638)	5589 (4720, 6470)	1169 (911, 1486)	327 (246, 440)
Gilan	4017 (3334, 4660)	3545 (2927, 4161)	727 (550, 935)	200 (145, 267)
Golestan	2463 (2105, 2935)	2230 (1913, 2648)	440 (345, 566)	114 (84, 157)
Hamadan	2763 (2357, 3191)	2286 (1913, 2685)	466 (350, 599)	129 (92, 176)
Hormozgan	1650 (1387, 1929)	1435 (1183, 1678)	294 (222, 374)	80 (59, 105)
Ilam	791 (665, 920)	696 (577, 806)	157 (116, 199)	47 (33, 61)
Iran (Islamic Republic of)	101431 (93512, 109806)	88768 (80979, 96544)	17990 (15591, 20328)	4971 (4107, 5860)
Isfahan	7153 (6049, 8173)	6264 (5237, 7192)	1326 (996, 1659)	373 (279, 482)
Kerman	3440 (2959, 4007)	3060 (2622, 3618)	604 (481, 760)	163 (123, 216)
Kermanshah	2858 (2396, 3416)	2412 (1984, 2859)	474 (363, 617)	129 (93, 173)
Khorasan-e-Razavi	8011 (6815, 9261)	7013 (5997, 8178)	1411 (1085, 1784)	386 (291, 510)
Khuzestan	5019 (4207, 6002)	4419 (3643, 5271)	844 (620, 1100)	223 (159, 301)
Kohgiluyeh and Boyer-Ahmad	772 (660, 886)	688 (590, 801)	151 (117, 195)	44 (32, 59)
Kurdistan	2150 (1831, 2465)	1868 (1557, 2148)	402 (311, 503)	115 (86, 152)
Lorestan	2027 (1721, 2407)	1719 (1421, 2074)	335 (252, 440)	91 (68, 126)
Markazi	2000 (1658, 2321)	1750 (1442, 2031)	359 (270, 449)	100 (71, 131)
Mazandaran	4535 (3803, 5349)	3858 (3175, 4451)	791 (608, 987)	220 (164, 292)
North Khorasan	1098 (914, 1279)	975 (809, 1141)	189 (147, 234)	50 (36, 66)
Qazvin	1687 (1433, 1995)	1463 (1237, 1734)	297 (230, 375)	82 (61, 108)
Qom	1239 (1038, 1474)	1157 (966, 1373)	205 (162, 264)	51 (38, 68)
Semnan	1023 (855, 1183)	889 (734, 1037)	188 (143, 239)	53 (39, 70)
Sistan and Baluchistan	2367 (2037, 2721)	2153 (1820, 2491)	406 (316, 495)	107 (83, 136)
South Khorasan	967 (809, 1142)	819 (688, 951)	174 (134, 222)	49 (35, 67)
Tehran	17113 (14412, 19667)	15239 (13010, 17661)	3155 (2505, 3867)	903 (694, 1201)
West Azarbayejan	3718 (3123, 4420)	3170 (2605, 3844)	608 (464, 790)	163 (121, 220)
Yazd	1478 (1249, 1741)	1253 (1052, 1466)	262 (204, 339)	75 (57, 105)
Zanjan	1552 (1260, 1866)	1375 (1142, 1654)	269 (200, 347)	74 (53, 99)

Table S15. The age-standardised rates of COVID-19 in 2020, 2021, 2022, and 2023 and its contribution to change in life expectancy in females

		COVID-19 age-standardised mortality rate				Life expectancy	Change in life expectancy		
**Province**	**SDI in 2019**	**2020**	**2021**	**2022**	**2023**	**2019**	**2019-2020**	**2020-2023**	**2019-2023**
Tehran	0·78	245.3 (209.4, 289.2)	225.8 (191, 262.7)	54.4 (42.4, 70.2)	17.2 (12.3, 24.4)	82.2	-4.31	4.35	0.04
Alborz	0·74	274.7 (211.9, 371.5)	263.2 (207.3, 343.2)	67.3 (47.5, 103.6)	21.4 (13.8, 37.2)	80.6	-4.09	3.91	-0.18
Mazandaran	0·73	279.9 (229.3, 347.3)	250.6 (204.2, 313)	60.8 (44.2, 85)	19 (13, 27)	80.3	-4.14	4.16	0.01
Isfahan	0·72	245.1 (204, 305.9)	228.6 (187, 287)	55.9 (42.2, 77.5)	17.4 (12.9, 25.5)	80.8	-3.96	3.83	-0.13
Fars	0·71	267.8 (222.6, 318.4)	237.2 (200, 283.5)	58.8 (44.9, 78.6)	18.5 (12.6, 25.5)	81	-4.64	4.59	-0.05
Gilan	0·71	295.8 (236, 372.7)	274.3 (224.7, 342.6)	68.6 (51.6, 95.4)	21.8 (14.7, 33.7)	79.7	-4.13	4.16	0.03
Semnan	0·71	268.5 (221.8, 326.2)	247.1 (207.1, 297.1)	59.1 (44.7, 78.3)	18 (13, 25.3)	80.4	-4.46	4.35	-0.12
Bushehr	0·7	262.3 (212.2, 328.2)	244.3 (197.8, 302.1)	56.4 (42.1, 79.1)	17.1 (11.6, 25.8)	78.8	-3.63	3.62	-0.01
Yazd	0·7	268.6 (221.4, 326.9)	250 (208.1, 297.6)	60.3 (45.3, 80.8)	18.8 (13.1, 26.7)	80.3	-4.32	4.19	-0.13
Ilam	0·69	263 (216.1, 311.9)	240.4 (194.7, 284.6)	59 (43.6, 76.7)	18.4 (13, 25.6)	80.6	-4.79	4.6	-0.19
Iran (Islamic Republic of)	0·69	250.4 (220.4, 291.5)	232.2 (204.4, 268)	54.3 (45.2, 68.9)	16.5 (13.2, 22.2)	79.7	-4.04	4	-0.04
Qazvin	0·69	257.5 (205.8, 310.2)	237.8 (191.9, 283.6)	54.7 (40.3, 71.9)	16.5 (11.5, 22.8)	79.6	-4.09	3.97	-0.12
Markazi	0·68	221.5 (184.4, 263.8)	212.1 (175.8, 249)	50 (38.5, 64.4)	15.1 (11, 20.4)	80.7	-3.93	3.85	-0.08
Qom	0·68	248.9 (186.7, 379.1)	233.9 (177.5, 339.9)	51.9 (35, 88.7)	15.8 (10.1, 31.2)	77.4	-2.53	2.52	-0.01
Kerman	0·67	271.8 (227.5, 332.3)	254.1 (209.3, 311.9)	60.5 (45.9, 83.7)	18.5 (13.1, 27.5)	79.4	-4.22	4.11	-0.12
Kermanshah	0·67	243.1 (198.2, 296.9)	218.7 (175.7, 264.8)	49.4 (35.7, 66)	14.6 (10, 20.9)	78.5	-3.83	3.81	-0.01
Khorasan-e-Razavi	0·67	257.7 (213.1, 306.2)	246.2 (209.8, 290.7)	55.7 (42.6, 73.5)	16.4 (11.7, 22.3)	79.2	-4.19	4.2	0.01
Kohgiluyeh and Boyer-Ahmad	0·67	209.9 (171, 251.4)	198.4 (161.9, 238.6)	46.6 (35, 60.8)	14.1 (9.5, 19.9)	80.3	-4.22	4.06	-0.16
East Azarbayejan	0·66	257.7 (206.6, 330.9)	240.6 (198.4, 311.1)	54 (40.7, 77.4)	16.3 (11.4, 25.6)	77.9	-3.37	3.31	-0.06
Hamadan	0·66	245.8 (200.6, 293)	219.8 (180.3, 265.1)	49.6 (37.1, 64)	14.7 (10.1, 20.2)	79.3	-4.04	3.93	-0.11

Hormozgan	0·66	221.6 (177.6, 263.2)	201.5 (164, 239)	47.7 (35.9, 61.4)	14.5 (10.2, 20)	81.2	-4.85	4.72	-0.13
Khuzestan	0·66	235.7 (197.6, 285.4)	221.2 (181, 268.4)	48.5 (37.9, 64.6)	14 (10.4, 19.3)	78.7	-4.2	4.15	-0.06
Lorestan	0·66	241.4 (194, 295.8)	218.2 (174.6, 265)	50.8 (36.4, 67.6)	15.4 (10.4, 22.6)	79.2	-3.8	3.81	0
Ardebil	0·65	248.2 (203.7, 300.2)	229.2 (186, 279.6)	51.4 (38.5, 66.9)	15.2 (10.8, 21.2)	78.9	-4.13	4.06	-0.07
Chahar Mahaal and Bakhtiari	0·65	212.9 (169.8, 260.1)	191 (150, 236.1)	45.4 (32.6, 61.5)	14 (9.6, 20.4)	80	-3.61	3.53	-0.08
Zanjan	0·65	263.7 (211.7, 318.4)	259.5 (207.8, 315.4)	56.3 (42.2, 73.6)	16.4 (11.2, 22.6)	76.2	-3.21	3.11	-0.11
Golestan	0·64	282.5 (234.3, 348.5)	277.6 (231, 336)	62.3 (47, 84)	18.1 (12.6, 25.5)	79.2	-5.12	5.05	-0.07
South Khorasan	0·64	252.4 (208.1, 308)	234.7 (192.1, 282)	54.3 (40.4, 70.7)	16.1 (11.7, 21.7)	79.9	-3.87	3.82	-0.05
Kurdistan	0·63	204.1 (166.7, 247.6)	192.8 (150.8, 234.8)	42.9 (30.1, 56.4)	12.7 (8.9, 17.5)	79.4	-3.65	3.56	-0.09
North Khorasan	0·63	293.8 (239.3, 357.3)	285.7 (239.9, 348.1)	62 (47.8, 80.1)	17.7 (13.1, 23.8)	77.4	-4.06	4.01	-0.04
West Azarbayejan	0·63	222.9 (173.6, 289.6)	200.3 (156.2, 258.1)	43 (31.4, 62)	12.7 (8.4, 21.2)	77.7	-3.21	3.11	-0.1
Sistan and Baluchistan	0·53	206.1 (166.4, 247.7)	201.2 (160.3, 238.3)	39.5 (29.1, 51)	10.8 (7.6, 14.7)	77	-4.31	4.34	0.04

SDI=Socio-Demographic Index

Provinces are sorted based on their socio-demographic index in 2019·

Table S16. The age-standardised rates of COVID-19 in 2020, 2021, 2022, and 2023 and its contribution to change in life expectancy in males

		COVID-19 age-standardised mortality rate				Life expectancy	Change in life expectancy		
**Province**	**SDI in 2019**	**2020**	**2021**	**2022**	**2023**	**2019**	**2019-2020**	**2020-2023**	**2019-2023**
Tehran	0·78	291 (247.5, 333.7)	244.4 (206.5, 285.1)	56.1 (44.3, 69.9)	17.3 (12.9, 23.1)	78.5	-4.52	4.54	0.02
Alborz	0·74	312 (267.1, 363.7)	271 (229.4, 319.7)	62.4 (47, 77.8)	18.9 (13.5, 25.1)	77.6	-4.4	4.28	-0.12
Mazandaran	0·73	331.7 (271.4, 391.8)	264.4 (214.5, 310.7)	61.3 (46.7, 78.8)	18.8 (13.6, 26.1)	77.5	-4.16	4.28	0.13
Isfahan	0·72	321.9 (268.7, 372.2)	268.4 (220.5, 314.7)	61.8 (47.6, 78.6)	18.6 (13.9, 24.9)	77.6	-4.37	4.4	0.02
Fars	0·71	356.1 (299.1, 415.6)	285.2 (235.6, 337.6)	66.7 (51.5, 85.2)	20.5 (14.6, 27.9)	76.8	-4.7	4.78	0.08
Gilan	0·71	332.5 (270.8, 388.1)	279.6 (228.2, 331.8)	64.1 (47.7, 81.7)	19.4 (13.7, 26.2)	76.5	-4.08	4.27	0.19
Semnan	0·71	347 (292.1, 404.9)	290.9 (240, 340.5)	67.7 (52.1, 85.8)	20.7 (15.2, 27.4)	76.9	-4.55	4.45	-0.1
Bushehr	0·7	347 (286.2, 413.4)	287.4 (234.8, 344.6)	67.2 (50.2, 86.1)	20.2 (14.1, 27.2)	76.5	-4.21	4.2	-0.01
Yazd	0·7	333.6 (278.2, 391.3)	274.4 (227.5, 323)	61.8 (47.5, 81.1)	18.6 (13.8, 26)	76.9	-4.45	4.46	0.01
Ilam	0·69	351.9 (296.3, 410)	300 (243.9, 353.1)	73.9 (55, 95.8)	23.2 (16.7, 31.4)	77	-5.01	4.72	-0.29
Iran (Islamic Republic of)	0·69	323.8 (296.8, 355.4)	268.7 (243, 297.2)	60.2 (51.5, 69.6)	18 (14.7, 21.6)	76.3	-4.31	4.31	0
Qazvin	0·69	340 (284.7, 403.5)	282.6 (233.6, 335.5)	64 (49.1, 81.1)	19.3 (14, 25.7)	76	-4.28	4.21	-0.07
Markazi	0·68	320.6 (264.9, 374)	269.4 (220.8, 315.9)	60 (44.3, 76.9)	17.9 (12.2, 24.1)	76.3	-3.9	3.99	0.08
Qom	0·68	348.7 (285.1, 438.4)	289.4 (237.8, 370)	61 (46.1, 82.3)	17.9 (13, 25.3)	74.2	-3	3.07	0.07
Kerman	0·67	339.1 (288.8, 402.3)	279.4 (233.5, 335.5)	62.2 (47.9, 81.8)	18.7 (13.7, 25.9)	75.7	-4.31	4.35	0.04
Kermanshah	0·67	349.5 (291.1, 417.3)	283.5 (232.6, 341.9)	61.8 (46.4, 81.2)	18.1 (12.8, 24.7)	74.2	-4.26	4.33	0.07
Khorasan-e-Razavi	0·67	328.9 (276.3, 380.9)	276 (233.2, 325.2)	60 (46.3, 76.4)	17.5 (13, 23.4)	75.9	-4.42	4.48	0.06
Kohgiluyeh and Boyer-Ahmad	0·67	300.1 (253.6, 348.8)	254.7 (213.9, 300.2)	60.6 (46.5, 79.6)	18.4 (12.9, 25.2)	78	-5.09	5.03	-0.06
East Azarbayejan	0·66	349.8 (287.2, 416.8)	289.8 (234.8, 349.8)	63.3 (47.5, 82.2)	18.7 (13, 25.9)	75.2	-3.89	3.87	-0.02
Hamadan	0·66	333.4 (283.5, 387.4)	271.3 (225.4, 320.9)	58.7 (44.5, 76.4)	17.1 (12.2, 23.5)	75.6	-4.69	4.76	0.07

Hormozgan	0·66	312.4 (260.6, 371.2)	253.6 (202.4, 302.1)	58.2 (42.2, 75.5)	17.3 (12.3, 23.9)	76.3	-4.7	4.56	-0.14
Khuzestan	0·66	313.5 (257.5, 376.2)	260.4 (212.3, 316.1)	55.2 (40.7, 73.9)	15.9 (11.3, 21.3)	74.8	-4.4	4.34	-0.06
Lorestan	0·66	337.2 (282, 409.9)	274.2 (224.3, 336.5)	60.7 (43.7, 81.9)	18.1 (13, 26.2)	74.1	-3.67	3.68	0.01
Ardebil	0·65	349.6 (286.6, 424.6)	284.8 (232.5, 349.1)	62.6 (46.6, 83.4)	18.6 (13, 26.3)	75.1	-4.02	4	-0.02
Chahar Mahaal and Bakhtiari	0·65	303.5 (247.1, 372.4)	241.2 (192.4, 300.8)	55.7 (40.5, 75)	17.1 (12.2, 24.1)	76.2	-3.64	3.6	-0.04
Zanjan	0·65	359.5 (288.4, 434.1)	307.4 (249.7, 373.8)	65.6 (47.8, 87.3)	19.2 (13.3, 26.2)	72.8	-3.44	3.41	-0.03
Golestan	0·64	390.5 (329, 466.4)	333.2 (278.9, 400.4)	75.4 (58.4, 99.5)	22.3 (16.3, 32.1)	75.2	-5.04	5	-0.04
South Khorasan	0·64	328.2 (273.9, 390.4)	268.5 (223.3, 317.1)	60.6 (45.9, 78.2)	17.8 (12.6, 24.9)	77.1	-4.23	4.23	0
Kurdistan	0·63	295.3 (250, 340.7)	249.6 (207.4, 290.5)	55.6 (42.8, 70.7)	16.4 (11.9, 22)	76.2	-4.25	4.01	-0.24
North Khorasan	0·63	346.8 (287.4, 405.1)	297.4 (243.3, 347.5)	62.6 (47.7, 79.2)	17.9 (12.7, 23.7)	75.1	-4.49	4.55	0.06
West Azarbayejan	0·63	325.3 (268.8, 395.2)	262.2 (209.9, 322.1)	56.4 (42.1, 74.9)	16.7 (12, 22.9)	75.2	-3.75	3.73	-0.01
Sistan and Baluchistan	0·53	337.1 (281.6, 387.2)	286.9 (239.1, 333.5)	58.3 (45.1, 73.5)	16.1 (11.8, 21.1)	73	-4.97	5.17	0.19

SDI=Socio-Demographic Index

Provinces are sorted based on their socio-demographic index in 2019·

Table S17. Contribution of communicable, maternal, neonatal, and nutritional (CMNN) diseases to change in life expectancy from 1990 to 2023 in both sexes combined

Province	LRI	Congenital Birth Defects	Neonatal Disorders	Diarrheal Diseases	Tuberculosis	Meningitis	Hemoglobinopathies and hemolytic anemias	Protein energy malnutrition
Alborz	0.45	0.33	1.02	0.15	0.05	0.05	0.01	0.01
Ardebil	0.32	0.89	1.11	0.22	0.04	0.08	0.02	0.02
Bushehr	0.24	0.52	1.59	0.28	0.05	0.07	0.03	0.1
Chahar Mahaal and Bakhtiari	0.32	0.65	1.22	0.1	0.02	0.05	0.03	0.03
East Azarbayejan	0.53	0.83	1.41	0.22	0.05	0.09	0.01	0.08
Fars	0.36	0.37	1.14	0.16	0.04	0.07	0.03	0.02
Gilan	0.17	0.4	0.86	0.12	0.04	0.04	0.02	0.01
Golestan	0.32	0.55	1.07	0.21	0.11	0.09	0.02	0.02
Hamadan	0.5	0.58	1.48	0.27	0.04	0.16	0.02	0.01
Hormozgan	0.73	0.28	1.39	0.64	0.11	0.11	0.06	0.02
Ilam	0.34	0.52	1.33	0.31	0.05	0.07	0.03	0.06
Iran (Islamic Republic of)	0.48	0.52	1.26	0.24	0.07	0.09	0.02	0.04
Isfahan	0.32	0.54	1.08	0.12	0.02	0.06	0.01	0.03
Kerman	0.5	0.29	1.24	0.33	0.07	0.08	0.03	0.02
Kermanshah	0.6	0.67	1.95	0.24	0.08	0.1	0.02	0.07
Khorasan-e-Razavi	0.73	0.74	1.92	0.38	0.13	0.12	0.02	0.09
Khuzestan	0.53	0.34	0.8	0.16	0.07	0.08	0.03	0.03
Kohgiluyeh and Boyer-Ahmad	0.63	0.64	1.28	0.34	0.04	0.21	0.06	0.03
Kurdistan	0.61	0.94	2.21	0.29	0.07	0.15	0.02	0.08
Lorestan	0.49	0.33	0.94	0.22	0.07	0.1	0.03	0.05
Markazi	0.53	0.52	1.55	0.2	0.07	0.09	0.01	0.01
Mazandaran	0.22	0.46	0.72	0.1	0.03	0.05	0.02	0.01
North Khorasan	0.81	0.52	1.36	0.47	0.05	0.12	0.01	0.03
Qazvin	0.43	0.56	1.47	0.26	0.03	0.1	0.01	0.02
Qom	0.55	0.49	1.13	0.17	0.07	0.07	0.01	0.02
Semnan	0.6	0.65	1.71	0.19	0.07	0.07	0.02	0.02
Sistan and Baluchistan	0.95	0.43	1.23	0.63	0.29	0.2	0.08	0.05
South Khorasan	1.17	0.4	1.59	0.44	0.11	0.13	0.01	0.04
Tehran	0.3	0.21	0.87	0.1	0.11	0.06	0.03	0.01
West Azarbayejan	0.43	0.85	1.54	0.29	0.05	0.09	0.01	0.03
Yazd	0.53	0.57	1.39	0.24	0.08	0.07	0.01	0.04
Zanjan	0.55	0.77	1.52	0.29	0.05	0.14	0.04	0.11

LRI=Lower respiratory infections

Table S18. Contribution of communicable, maternal, neonatal, and nutritional (CMNN) diseases to change in life expectancy from 1990 to 2023 in females

Province	LRI	Congenital Birth Defects	Neonatal Disorders	Diarrheal Diseases	Tuberculosis	Meningitis	Hemoglobinopathies and hemolytic anemias	Protein energy malnutrition
Alborz	0.46	0.36	0.86	0.18	0.04	0.05	0.01	0.01
Ardebil	0.42	1.24	1.07	0.27	0.04	0.12	0.03	0.02
Bushehr	0.35	0.64	1.45	0.3	0.04	0.08	0.04	0.12
Chahar Mahaal and Bakhtiari	0.42	0.94	1.22	0.13	0.02	0.07	0.05	0.03
East Azarbayejan	0.72	1.17	1.39	0.27	0.05	0.12	0.02	0.13
Fars	0.42	0.43	1.08	0.18	0.03	0.09	0.04	0.02
Gilan	0.22	0.49	0.83	0.15	0.03	0.05	0.03	0.01
Golestan	0.36	0.69	0.93	0.23	0.08	0.1	0.02	0.02
Hamadan	0.62	0.77	1.47	0.32	0.03	0.2	0.01	0.01
Hormozgan	0.87	0.36	1.25	0.67	0.08	0.13	0.06	0.02
Ilam	0.5	0.73	1.32	0.39	0.04	0.08	0.03	0.08
Iran (Islamic Republic of)	0.57	0.67	1.17	0.27	0.06	0.1	0.03	0.06
Isfahan	0.34	0.65	0.95	0.13	0.02	0.07	0.01	0.04
Kerman	0.58	0.33	1.12	0.36	0.07	0.09	0.02	0.02
Kermanshah	0.71	0.87	1.85	0.28	0.07	0.12	0.03	0.09
Khorasan-e-Razavi	0.91	0.93	1.8	0.41	0.12	0.14	0.02	0.15
Khuzestan	0.57	0.41	0.68	0.18	0.06	0.09	0.02	0.04
Kohgiluyeh and Boyer-Ahmad	0.8	0.83	1.18	0.42	0.04	0.24	0.07	0.04
Kurdistan	0.79	1.31	2.22	0.38	0.08	0.19	0.02	0.14
Lorestan	0.64	0.48	0.9	0.24	0.05	0.12	0.04	0.08
Markazi	0.66	0.72	1.55	0.24	0.06	0.11	0.01	0.02
Mazandaran	0.24	0.49	0.62	0.11	0.02	0.05	0.02	0.02
North Khorasan	0.98	0.6	1.16	0.53	0.05	0.13	0	0.03
Qazvin	0.51	0.75	1.35	0.32	0.03	0.13	0.02	0.03
Qom	0.59	0.58	0.95	0.18	0.06	0.07	0.01	0.04
Semnan	0.72	0.85	1.56	0.22	0.06	0.08	0.02	0.03
Sistan and Baluchistan	1.1	0.52	1.08	0.74	0.31	0.24	0.11	0.05
South Khorasan	1.26	0.45	1.44	0.46	0.07	0.14	0	0.05
Tehran	0.34	0.23	0.76	0.12	0.09	0.06	0.03	0.01
West Azarbayejan	0.58	1.23	1.61	0.39	0.04	0.11	0.02	0.04
Yazd	0.64	0.7	1.25	0.26	0.07	0.07	0.01	0.05
Zanjan	0.77	1.13	1.55	0.38	0.04	0.18	0.05	0.22

LRI=Lower respiratory infections

Table S19. Contribution of communicable, maternal, neonatal, and nutritional (CMNN) diseases to change in life expectancy from 1990 to 2023 in males

Province	LRI	Congenital Birth Defects	Neonatal Disorders	Diarrheal Diseases	Tuberculosis	Meningitis	Hemoglobinopathies and hemolytic anemias	Protein energy malnutrition
Alborz	0.45	0.29	1.17	0.13	0.05	0.05	0.01	0.01
Ardebil	0.23	0.56	1.15	0.18	0.03	0.05	0.01	0.01
Bushehr	0.26	0.41	1.72	0.28	0.06	0.06	0.03	0.08
Chahar Mahaal and Bakhtiari	0.2	0.39	1.22	0.08	0.02	0.04	0.02	0.03
East Azarbayejan	0.36	0.52	1.42	0.17	0.05	0.07	0.01	0.04
Fars	0.31	0.32	1.19	0.15	0.04	0.06	0.03	0.01
Gilan	0.13	0.31	0.88	0.1	0.05	0.04	0.02	0.01
Golestan	0.29	0.42	1.19	0.19	0.12	0.08	0.02	0.02
Hamadan	0.4	0.42	1.48	0.23	0.04	0.13	0.02	0.01
Hormozgan	0.6	0.2	1.51	0.62	0.13	0.1	0.05	0.03
Ilam	0.2	0.34	1.34	0.23	0.05	0.06	0.02	0.04
Iran (Islamic Republic of)	0.39	0.38	1.34	0.2	0.08	0.08	0.02	0.02
Isfahan	0.27	0.43	1.2	0.11	0.03	0.05	0.01	0.02
Kerman	0.47	0.24	1.36	0.3	0.08	0.07	0.03	0.02
Kermanshah	0.51	0.5	2.04	0.21	0.09	0.09	0.02	0.04
Khorasan-e-Razavi	0.57	0.55	2.03	0.35	0.13	0.1	0.02	0.04
Khuzestan	0.51	0.27	0.9	0.15	0.09	0.07	0.03	0.02
Kohgiluyeh and Boyer-Ahmad	0.45	0.47	1.37	0.26	0.04	0.17	0.04	0.02
Kurdistan	0.45	0.6	2.19	0.21	0.06	0.12	0.01	0.03
Lorestan	0.37	0.2	0.98	0.2	0.08	0.08	0.03	0.02
Markazi	0.42	0.34	1.55	0.16	0.08	0.08	0.01	0.01
Mazandaran	0.2	0.43	0.82	0.09	0.04	0.04	0.02	0.01
North Khorasan	0.67	0.44	1.54	0.41	0.06	0.11	0.01	0.02
Qazvin	0.34	0.4	1.58	0.21	0.03	0.07	0.01	0.01
Qom	0.51	0.4	1.29	0.16	0.08	0.07	0.01	0.01
Semnan	0.5	0.48	1.84	0.17	0.07	0.06	0.02	0.02
Sistan and Baluchistan	0.78	0.35	1.37	0.54	0.27	0.16	0.06	0.05
South Khorasan	1.01	0.36	1.74	0.42	0.13	0.11	0.01	0.02
Tehran	0.27	0.19	0.98	0.09	0.13	0.06	0.04	0.01
West Azarbayejan	0.28	0.5	1.48	0.19	0.05	0.06	0.01	0.02
Yazd	0.44	0.44	1.52	0.22	0.09	0.06	0.01	0.04
Zanjan	0.33	0.42	1.5	0.21	0.05	0.1	0.02	0.01

LRI=Lower respiratory infections

Table S20. Contribution of cardiovascular diseases to change in life expectancy from 1990 to 2023 in both sexes combined

Province	Ischemic Heart Disease	Stroke	Hypertensive Heart Disease	Other cardiovascular and circulatory diseases	Atrial fibrillation and flutter	Cardiomyopathy and myocarditis
Alborz	1.51	1.42	0.19	0.03	-0.01	0.02
Ardebil	1.85	1.33	0.17	0.14	-0.01	0.02
Bushehr	2.14	1.97	0.24	0.01	0	0.01
Chahar Mahaal and Bakhtiari	2.28	1.22	0.21	0	-0.01	0.01
East Azarbayejan	1.95	1.32	0.1	-0.01	-0.01	0.02
Fars	4.11	1.63	0.28	0.05	0	0.01
Gilan	3.37	1.81	0.14	0.05	0	0.01
Golestan	3.05	1.77	0.11	-0.02	-0.01	0.01
Hamadan	2.84	1.63	0.18	0.01	-0.01	0.02
Hormozgan	2.72	1.71	0.32	0.02	-0.01	0.01
Ilam	2.56	0.86	0.13	0	-0.01	0.01
Iran (Islamic Republic of)	2.61	1.63	0.24	0.04	0	0.02
Isfahan	3.09	1.45	0.4	0.03	0	0.02
Kerman	2.89	1.23	0.09	0.02	-0.01	0.01
Kermanshah	2.51	1.75	0.41	0.05	0	0.02
Khorasan-e-Razavi	2.69	1.68	0.22	0.01	0	0.02
Khuzestan	2.25	1.7	0.4	0.17	0	0.05
Kohgiluyeh and Boyer-Ahmad	2.36	1.55	0.25	0.12	-0.01	0.04
Kurdistan	1.82	1.29	0.06	-0.02	-0.02	0.01
Lorestan	1.96	1.78	0.13	0.08	-0.01	0.11
Markazi	2.44	1.79	0.28	0	0	0.01
Mazandaran	3.32	1.72	0.16	0.09	0	0.03
North Khorasan	1.96	1.56	0.45	0.14	-0.01	0.03
Qazvin	1.72	1.24	0.18	0.04	-0.01	0.01
Qom	1.33	1.54	0.28	0.06	0	0.02
Semnan	1.67	1.45	0.23	-0.01	0	0.01
Sistan and Baluchistan	2.84	1.62	0.37	0.05	-0.01	0.01
South Khorasan	1.79	1.45	0.34	0.16	0	0.02
Tehran	2.6	2.07	0.26	0.04	0.01	0.04
West Azarbayejan	2.02	1.13	0.1	-0.01	-0.01	0.02
Yazd	1.98	1.63	0.17	0	-0.01	0.01
Zanjan	1.72	1.02	0.09	0.02	-0.01	0.02

Table S21. Contribution of cardiovascular diseases to change in life expectancy from 1990 to 2023 in females

Province	Ischemic Heart Disease	Stroke	Hypertensive Heart Disease	Other cardiovascular and circulatory diseases	Atrial fibrillation and flutter	Cardiomyopathy and myocarditis
Alborz	1.38	1.41	0.07	0.02	-0.01	0.02
Ardebil	2.29	1.49	0.05	0.13	-0.01	0.02
Bushehr	2.61	1.88	0.11	-0.01	0	0
Chahar Mahaal and Bakhtiari	2.56	1.44	0.08	0.02	-0.01	0.02
East Azarbayejan	2.18	1.64	-0.04	0.01	-0.01	0.02
Fars	4.39	2.01	0.28	0.06	0.01	0.01
Gilan	3.22	2.03	0.05	0.04	0	0.01
Golestan	2.88	2.02	-0.02	-0.04	-0.01	0
Hamadan	2.35	1.65	0.02	0.01	-0.01	0.01
Hormozgan	2.99	1.87	0.16	0.02	-0.01	0.01
Ilam	2.87	0.99	0.03	0.02	-0.01	0.01
Iran (Islamic Republic of)	2.84	1.95	0.18	0.05	0	0.02
Isfahan	3.18	1.78	0.43	0.04	0	0.02
Kerman	2.7	1.38	0.08	-0.01	0	0
Kermanshah	2.49	2.04	0.28	0.04	0	0.01
Khorasan-e-Razavi	3.21	2.09	0.22	0.02	0	0.01
Khuzestan	2.6	2.08	0.33	0.15	0	0.04
Kohgiluyeh and Boyer-Ahmad	2.4	1.76	0.13	0.13	-0.02	0.04
Kurdistan	2.09	1.53	-0.06	-0.01	-0.02	0.01
Lorestan	2.42	1.93	0.06	0.08	-0.01	0.13
Markazi	2.53	2.07	0.17	0	0	0.01
Mazandaran	3.39	1.9	0.09	0.08	0	0.03
North Khorasan	2.03	1.76	0.29	0.1	0	0.03
Qazvin	1.92	1.47	0.11	0.04	-0.01	0.01
Qom	1.38	1.67	0.22	0.05	0	0.02
Semnan	2.11	1.89	0.21	0	-0.01	0.01
Sistan and Baluchistan	3.24	2.05	0.35	0.05	0	0.01
South Khorasan	2.25	1.97	0.38	0.17	0.01	0.02
Tehran	3.09	2.44	0.25	0.05	0.02	0.04
West Azarbayejan	1.89	1.31	-0.04	-0.01	-0.01	0.01
Yazd	2.04	1.91	0.1	0	-0.01	0.01
Zanjan	1.89	1.19	0.01	0.06	-0.01	0.03

Table S22. Contribution of cardiovascular diseases to change in life expectancy from 1990 to 2023 in males

Province	Ischemic Heart Disease	Stroke	Hypertensive Heart Disease	Other cardiovascular and circulatory diseases	Atrial fibrillation and flutter	Cardiomyopathy and myocarditis
Alborz	1.53	1.26	0.28	0.04	0	0.02
Ardebil	1.55	1.16	0.25	0.15	0	0.02
Bushehr	1.79	1.4	0.18	-0.01	0	0.01
Chahar Mahaal and Bakhtiari	2.18	0.97	0.47	0.01	-0.01	0.02
East Azarbayejan	1.76	1.11	0.17	-0.02	-0.01	0.02
Fars	3.83	1.31	0.29	0.05	0	0.02
Gilan	3.44	1.66	0.2	0.05	0	0.01
Golestan	3.08	1.55	0.2	0.01	0	0.01
Hamadan	3.08	1.58	0.3	0.02	0	0.02
Hormozgan	2.38	1.55	0.4	0.03	-0.01	0.01
Ilam	2.26	0.67	0.18	-0.01	-0.01	0
Iran (Islamic Republic of)	2.38	1.41	0.29	0.05	-0.01	0.03
Isfahan	3.06	1.24	0.44	0.03	0	0.02
Kerman	2.74	0.99	0.19	0.01	0	0.01
Kermanshah	2.43	1.56	0.45	0.06	0	0.03
Khorasan-e-Razavi	2.24	1.35	0.23	0	-0.01	0.02
Khuzestan	1.85	1.42	0.44	0.19	0	0.05
Kohgiluyeh and Boyer-Ahmad	2.32	1.36	0.4	0.12	-0.01	0.04
Kurdistan	1.54	1.1	0.15	-0.03	-0.01	0
Lorestan	1.61	1.39	0.22	0.07	0	0.08
Markazi	2.28	1.46	0.33	-0.01	0	0.01
Mazandaran	3.19	1.54	0.26	0.09	0	0.03
North Khorasan	1.83	1.37	0.55	0.16	-0.01	0.04
Qazvin	1.53	1.03	0.25	0.04	-0.01	0.01
Qom	1.27	1.4	0.33	0.07	0	0.03
Semnan	1.3	1.1	0.24	-0.01	0	0.01
Sistan and Baluchistan	2.68	1.34	0.35	0.07	-0.01	0.02
South Khorasan	1.54	1.2	0.36	0.17	0	0.02
Tehran	2.1	1.67	0.26	0.03	0	0.03
West Azarbayejan	2.07	1.01	0.22	-0.01	0	0.02
Yazd	1.92	1.39	0.23	0	-0.01	0.01
Zanjan	1.57	0.88	0.17	-0.01	-0.01	0.01

Table S23. Contribution of neoplasms to change in life expectancy from 1990 to 2023 in both sexes combined

Province	Stomach cancer	Lung cancer	Colorectal cancer	Leukemia	Prostate cancer	Brain cancer	Breast cancer	Pancreatic cancer	Esophageal cancer	Liver cancer
Alborz	0.11	-0.02	-0.03	0.04	0	-0.01	-0.05	-0.03	0.01	0.01
Ardebil	0.27	-0.06	-0.06	0.1	0.01	-0.02	-0.05	-0.03	0.05	0.03
Bushehr	0.13	-0.01	-0.03	0.06	-0.02	-0.01	-0.05	-0.03	0.01	0.02
Chahar Mahaal and Bakhtiari	0.21	-0.02	-0.04	0.1	0	0	-0.03	-0.02	0.01	0.03
East Azarbayejan	0.23	-0.03	-0.04	0.14	0	0.03	-0.04	-0.04	0.05	0.02
Fars	0.3	0.1	0.02	0.12	0.03	0.03	-0.01	-0.01	0.03	0.06
Gilan	0.4	0.1	0.03	0.08	0.04	0	-0.01	0	0.06	0.05
Golestan	0.26	0.03	-0.02	0.05	0.02	-0.02	-0.05	-0.02	0.13	0.06
Hamadan	0.28	0.03	-0.02	0.13	0.02	0.01	-0.04	-0.02	0.02	0.05
Hormozgan	0.14	-0.01	-0.02	0.07	0.01	-0.01	-0.05	-0.01	0.01	0.02
Ilam	0.28	-0.04	-0.05	0.06	0.01	0	-0.04	-0.04	0.05	0.03
Iran (Islamic Republic of)	0.27	0.04	0	0.11	0.02	0.02	-0.02	-0.02	0.05	0.04
Isfahan	0.23	0.06	0.01	0.1	0.03	0.02	-0.01	-0.02	0.03	0.05
Kerman	0.2	0.06	-0.01	0.08	0.02	0.02	-0.04	-0.02	0.02	0.04
Kermanshah	0.28	0.07	0	0.11	0.03	0.03	-0.04	-0.03	0.05	0.06
Khorasan-e-Razavi	0.38	0.05	0	0.14	0.01	0.03	-0.02	-0.02	0.09	0.06
Khuzestan	0.12	-0.02	-0.03	0.05	0	-0.02	-0.06	-0.03	0.02	0.03
Kohgiluyeh and Boyer-Ahmad	0.23	-0.04	-0.03	0.09	0.01	-0.03	-0.04	-0.03	0.01	0.02
Kurdistan	0.32	-0.04	-0.07	0.13	-0.01	0.01	-0.05	-0.04	0.03	0.04
Lorestan	0.21	-0.05	-0.05	0.07	0	-0.02	-0.05	-0.04	0.03	0.03
Markazi	0.33	0.06	0	0.11	0.03	0.02	-0.03	-0.02	0.04	0.05
Mazandaran	0.38	0.07	0.03	0.09	0.04	0.01	-0.01	0	0.07	0.05
North Khorasan	0.35	0.01	-0.02	0.07	0.01	-0.02	-0.05	-0.02	0.08	0.05
Qazvin	0.16	-0.03	-0.06	0.07	-0.01	-0.01	-0.06	-0.04	0.02	0.03
Qom	0.22	0.03	-0.01	0.08	0.02	-0.01	-0.06	-0.02	0.04	0.03
Semnan	0.22	0	-0.04	0.08	0	0.01	-0.04	-0.04	0.04	0.02
Sistan and Baluchistan	0.33	0.07	0	0.12	0.03	0.01	-0.04	-0.01	0.06	0.08
South Khorasan	0.29	0.02	0	0.09	0.03	-0.01	-0.04	-0.01	0.06	0.04
Tehran	0.25	0.08	0.08	0.12	0.03	0.06	0.08	0.02	0.04	0.04
West Azarbayejan	0.25	-0.02	-0.06	0.11	0.01	0.01	-0.07	-0.05	0.04	0.03
Yazd	0.21	0.07	0	0.1	0.02	0.02	-0.02	-0.03	0.04	0.04
Zanjan	0.23	-0.02	-0.04	0.14	0.01	0.01	-0.05	-0.03	0.03	0.02

Table S24. Contribution of neoplasms to change in life expectancy from 1990 to 2023 in females

Province	Stomach cancer	Breast cancer	Lung cancer	Colorectal cancer	Leukemia	Brain cancer	Pancreatic cancer	Liver cancer	Esophageal cancer
Alborz	0.09	-0.09	-0.03	-0.02	0.04	-0.01	-0.02	0	0.01
Ardebil	0.22	-0.11	-0.06	-0.05	0.15	-0.01	-0.03	0.03	0.03
Bushehr	0.13	-0.09	-0.02	-0.01	0.07	-0.01	-0.02	0.03	0.02
Chahar Mahaal and Bakhtiari	0.16	-0.06	-0.04	-0.04	0.12	0.01	-0.01	0.03	0.01
East Azarbayejan	0.21	-0.08	-0.03	-0.04	0.18	0.03	-0.03	0.03	0.05
Fars	0.27	-0.01	0.06	0.03	0.13	0.03	0	0.06	0.03
Gilan	0.31	-0.03	0.03	0.03	0.09	0	-0.01	0.03	0.04
Golestan	0.2	-0.1	-0.01	-0.03	0.06	-0.02	-0.02	0.04	0.11
Hamadan	0.17	-0.08	-0.03	-0.03	0.15	0.02	-0.02	0.03	0.01
Hormozgan	0.11	-0.11	-0.03	-0.02	0.07	-0.01	-0.01	0.01	0.01
Ilam	0.28	-0.08	-0.03	-0.04	0.07	0	-0.04	0.04	0.06
Iran (Islamic Republic of)	0.24	-0.03	0.01	0.02	0.12	0.02	-0.01	0.05	0.04
Isfahan	0.19	-0.01	0.03	0.02	0.1	0.02	-0.02	0.06	0.03
Kerman	0.15	-0.08	0.01	-0.01	0.07	0.01	-0.02	0.04	0.02
Kermanshah	0.22	-0.07	0.01	0	0.11	0.03	-0.03	0.06	0.04
Khorasan-e-Razavi	0.37	-0.03	0.05	0.02	0.17	0.04	-0.01	0.07	0.1
Khuzestan	0.12	-0.11	-0.02	-0.02	0.06	-0.01	-0.03	0.03	0.02
Kohgiluyeh and Boyer-Ahmad	0.16	-0.09	-0.06	-0.03	0.11	-0.02	-0.03	0.02	0.01
Kurdistan	0.29	-0.09	-0.04	-0.05	0.17	0.02	-0.03	0.04	0.03
Lorestan	0.21	-0.09	-0.05	-0.02	0.09	-0.01	-0.03	0.04	0.04
Markazi	0.27	-0.05	0.01	0	0.12	0.02	-0.02	0.05	0.03
Mazandaran	0.31	-0.02	0.03	0.04	0.09	0.02	0	0.04	0.06
North Khorasan	0.24	-0.08	-0.02	-0.01	0.07	-0.02	-0.02	0.04	0.06
Qazvin	0.15	-0.11	-0.03	-0.04	0.1	0	-0.04	0.03	0.02
Qom	0.16	-0.12	-0.02	-0.01	0.09	-0.01	-0.03	0.02	0.02
Semnan	0.18	-0.08	0	-0.02	0.08	0.01	-0.03	0.03	0.04
Sistan and Baluchistan	0.26	-0.07	0.01	-0.01	0.13	0.01	-0.01	0.08	0.06
South Khorasan	0.22	-0.07	-0.01	0.01	0.08	-0.02	-0.01	0.04	0.05
Tehran	0.28	0.19	0.09	0.16	0.14	0.08	0.05	0.06	0.06
West Azarbayejan	0.13	-0.13	-0.07	-0.07	0.13	0.02	-0.05	0.01	0.01
Yazd	0.16	-0.05	0.02	0.01	0.11	0.02	-0.03	0.04	0.04
Zanjan	0.17	-0.09	-0.05	-0.05	0.19	0.02	-0.03	0.02	0.02

Table S25. Contribution of neoplasms to change in life expectancy from 1990 to 2023 in males

Province	Stomach cancer	Lung cancer	Prostate cancer	Colorectal cancer	Leukemia	Brain cancer	Pancreatic cancer	Esophageal cancer	Liver cancer
Alborz	0.12	-0.01	0	-0.04	0.03	-0.01	-0.03	0.01	0.01
Ardebil	0.25	-0.08	-0.01	-0.07	0.05	-0.03	-0.03	0.04	0.03
Bushehr	0.13	0.02	0.01	-0.03	0.05	-0.01	-0.03	0.01	0.02
Chahar Mahaal and Bakhtiari	0.22	-0.05	-0.05	-0.06	0.06	-0.01	-0.04	0.01	0.02
East Azarbayejan	0.21	-0.04	-0.02	-0.06	0.1	0.02	-0.04	0.05	0.02
Fars	0.31	0.13	0.05	0.01	0.12	0.02	-0.01	0.03	0.05
Gilan	0.47	0.16	0.07	0.03	0.07	0	0	0.07	0.06
Golestan	0.28	0.05	0.02	-0.02	0.04	-0.02	-0.02	0.15	0.07
Hamadan	0.33	0.05	0.02	-0.01	0.11	0.01	-0.02	0.03	0.06
Hormozgan	0.14	-0.02	-0.01	-0.03	0.06	0	-0.02	0.01	0.02
Ilam	0.25	-0.06	-0.01	-0.06	0.05	-0.01	-0.05	0.04	0.02
Iran (Islamic Republic of)	0.28	0.04	0.01	-0.02	0.09	0.01	-0.03	0.05	0.04
Isfahan	0.26	0.08	0.04	0	0.09	0.01	-0.02	0.03	0.05
Kerman	0.23	0.1	0.03	0	0.09	0.02	-0.02	0.02	0.05
Kermanshah	0.29	0.08	0.03	-0.01	0.1	0.04	-0.03	0.05	0.06
Khorasan-e-Razavi	0.36	0.04	0	-0.02	0.13	0.03	-0.03	0.08	0.05
Khuzestan	0.11	-0.03	-0.01	-0.05	0.04	-0.03	-0.04	0.01	0.02
Kohgiluyeh and Boyer-Ahmad	0.27	-0.03	-0.03	-0.04	0.07	-0.04	-0.04	0.01	0.03
Kurdistan	0.29	-0.07	-0.05	-0.08	0.09	0.01	-0.05	0.02	0.04
Lorestan	0.17	-0.07	-0.02	-0.06	0.04	-0.03	-0.05	0.02	0.02
Markazi	0.38	0.09	0.05	0.01	0.11	0.02	-0.02	0.04	0.05
Mazandaran	0.42	0.1	0.07	0.02	0.09	0.01	0	0.08	0.06
North Khorasan	0.4	0.03	0.02	-0.03	0.07	-0.02	-0.02	0.1	0.05
Qazvin	0.15	-0.05	-0.03	-0.07	0.05	-0.02	-0.04	0.02	0.02
Qom	0.27	0.07	0.04	0	0.08	-0.01	-0.02	0.06	0.04
Semnan	0.23	0	-0.01	-0.05	0.08	0	-0.05	0.03	0.01
Sistan and Baluchistan	0.36	0.09	0.03	0.01	0.09	0.01	-0.01	0.05	0.08
South Khorasan	0.33	0.03	0.03	-0.02	0.09	-0.01	-0.02	0.06	0.03
Tehran	0.22	0.07	0.03	0.01	0.11	0.04	-0.01	0.02	0.02
West Azarbayejan	0.3	0	-0.01	-0.04	0.08	0.01	-0.04	0.05	0.03
Yazd	0.25	0.11	0.03	0	0.1	0.02	-0.03	0.04	0.04
Zanjan	0.25	-0.01	0	-0.04	0.09	0.01	-0.04	0.03	0.02

Table S26. Contribution of major non-communicable diseases to change in life expectancy from 1990 to 2023 in both sexes combined

Province	CKD	COPD	Diabetes	Cirrhosis	Drug use disorders
Alborz	0.01	0.01	-0.15	0.16	-0.03
Ardebil	0.04	-0.02	-0.09	0.15	-0.02
Bushehr	0.04	-0.08	-0.31	0.21	0.01
Chahar Mahaal and Bakhtiari	0.03	0.06	-0.13	0.14	-0.01
East Azarbayejan	0.07	-0.01	-0.1	0.17	0.01
Fars	0.17	0.01	-0.1	0.28	0.08
Gilan	0.14	-0.01	-0.16	0.15	0.01
Golestan	0.07	-0.05	-0.15	0.22	0
Hamadan	0.1	0.01	-0.06	0.25	0.01
Hormozgan	0.14	0.04	-0.12	0.34	-0.01
Ilam	0	0.04	-0.16	0.24	-0.01
Iran (Islamic Republic of)	0.12	0.02	-0.09	0.23	0.02
Isfahan	0.15	0.02	-0.13	0.18	0.02
Kerman	0.09	0.09	-0.13	0.31	0.01
Kermanshah	0.14	0.11	-0.08	0.24	0.08
Khorasan-e-Razavi	0.18	0	-0.11	0.32	0.02
Khuzestan	0.08	0.04	-0.17	0.2	-0.01
Kohgiluyeh and Boyer-Ahmad	0.04	0.09	-0.07	0.14	-0.01
Kurdistan	0.1	0.04	-0.14	0.21	-0.01
Lorestan	0.07	0.02	-0.09	0.23	-0.01
Markazi	0.13	0	-0.13	0.27	0.01
Mazandaran	0.11	0.03	-0.12	0.17	0.01
North Khorasan	0.09	-0.03	-0.08	0.29	-0.01
Qazvin	0.03	0	-0.1	0.39	-0.04
Qom	0.11	0.03	-0.06	0.25	0
Semnan	0.04	0	-0.17	0.26	-0.01
Sistan and Baluchistan	0.33	0.1	-0.14	0.31	0.11
South Khorasan	0.15	0	-0.06	0.33	0
Tehran	0.18	0.04	0.15	0.21	0.04
West Azarbayejan	0.05	0.01	-0.13	0.17	0
Yazd	0.11	0.08	-0.23	0.28	0.02
Zanjan	0.03	0.03	-0.11	0.29	-0.02

CKD=Chronic Kidney Disease; COPD=Chronic Obstructive Pulmonary Disease.

Table S27. Contribution of major non-communicable diseases to change in life expectancy from 1990 to 2023 in females

Province	CKD	Diabetes	COPD	Cirrhosis	Drug use disorders
Alborz	-0.04	-0.06	-0.06	0.14	0
Ardebil	-0.07	-0.06	-0.07	0.18	0
Bushehr	0.02	-0.15	-0.07	0.18	0.01
Chahar Mahaal and Bakhtiari	-0.03	-0.07	-0.04	0.15	0
East Azarbayejan	0	-0.05	-0.12	0.19	0.02
Fars	0.12	-0.04	-0.08	0.23	0.04
Gilan	0.05	-0.13	-0.06	0.14	0.01
Golestan	-0.02	-0.12	-0.12	0.18	0.01
Hamadan	-0.03	-0.03	-0.08	0.24	0
Hormozgan	0.03	-0.06	-0.06	0.33	0
Ilam	-0.1	-0.1	-0.09	0.27	0
Iran (Islamic Republic of)	0.06	-0.02	-0.09	0.22	0.01
Isfahan	0.07	-0.09	-0.11	0.17	0.01
Kerman	0.02	-0.05	-0.17	0.24	0
Kermanshah	0.06	-0.03	-0.06	0.2	0.01
Khorasan-e-Razavi	0.14	-0.03	-0.11	0.3	0.02
Khuzestan	0.04	-0.02	-0.05	0.18	0
Kohgiluyeh and Boyer-Ahmad	-0.03	-0.06	-0.06	0.12	0
Kurdistan	0.04	-0.09	-0.12	0.21	0
Lorestan	0	-0.02	-0.08	0.21	0
Markazi	0.05	-0.06	-0.1	0.26	0
Mazandaran	0.04	-0.02	-0.04	0.16	0.01
North Khorasan	0.01	-0.02	-0.14	0.26	0
Qazvin	-0.04	-0.04	-0.1	0.38	0
Qom	0.02	-0.02	-0.08	0.19	0
Semnan	0	-0.08	-0.1	0.23	0
Sistan and Baluchistan	0.33	-0.12	-0.1	0.29	0.07
South Khorasan	0.08	-0.07	-0.26	0.3	0.01
Tehran	0.18	0.28	-0.04	0.19	0.04
West Azarbayejan	-0.04	-0.11	-0.1	0.19	0
Yazd	0.04	-0.08	-0.11	0.27	0.01
Zanjan	-0.04	-0.08	-0.1	0.28	0

CKD=Chronic Kidney Disease; COPD=Chronic Obstructive Pulmonary Disease.

Table S28. Contribution of major non-communicable diseases to change in life expectancy from 1990 to 2023 in males

Province	COPD	Diabetes	CKD	Cirrhosis	Drug use disorders
Alborz	0.07	-0.17	0.06	0.16	-0.05
Ardebil	0	-0.12	0.1	0.12	-0.03
Bushehr	0.02	-0.27	0.1	0.21	0.01
Chahar Mahaal and Bakhtiari	0.11	-0.26	0.05	0.14	-0.02
East Azarbayejan	0.06	-0.14	0.11	0.16	0
Fars	0.08	-0.14	0.2	0.31	0.13
Gilan	0.04	-0.19	0.2	0.16	0.01
Golestan	-0.02	-0.18	0.14	0.23	0
Hamadan	0.07	-0.08	0.19	0.24	0.02
Hormozgan	0.1	-0.17	0.21	0.33	-0.01
Ilam	0.12	-0.17	0.05	0.21	-0.02
Iran (Islamic Republic of)	0.11	-0.16	0.16	0.24	0.02
Isfahan	0.09	-0.21	0.2	0.18	0.03
Kerman	0.34	-0.13	0.2	0.32	0.02
Kermanshah	0.19	-0.1	0.18	0.26	0.14
Khorasan-e-Razavi	0.07	-0.15	0.21	0.34	0.02
Khuzestan	0.11	-0.3	0.11	0.21	-0.03
Kohgiluyeh and Boyer-Ahmad	0.19	-0.12	0.07	0.15	-0.03
Kurdistan	0.11	-0.16	0.13	0.2	-0.01
Lorestan	0.09	-0.12	0.1	0.22	0
Markazi	0.08	-0.13	0.19	0.27	0.01
Mazandaran	0.08	-0.19	0.18	0.17	0.01
North Khorasan	0.06	-0.1	0.17	0.29	-0.01
Qazvin	0.06	-0.13	0.09	0.39	-0.07
Qom	0.12	-0.1	0.17	0.29	-0.01
Semnan	0.08	-0.25	0.08	0.28	-0.02
Sistan and Baluchistan	0.2	-0.19	0.31	0.31	0.14
South Khorasan	0.1	-0.09	0.19	0.35	-0.01
Tehran	0.12	0.05	0.17	0.22	0.03
West Azarbayejan	0.06	-0.13	0.11	0.15	0
Yazd	0.24	-0.36	0.17	0.28	0.03
Zanjan	0.11	-0.12	0.07	0.28	-0.04

CKD=Chronic Kidney Disease; COPD=Chronic Obstructive Pulmonary Disease.

Table S29. Contribution of injuries to change in life expectancy from 1990 to 2023 in both sexes combined

Province	Transport injuries	Self harm	Falls	Interpersonal violence	Fire, heat, and hot substances	Exposure to mechanical forces	Adverse effects of medical treatment	Drowning	Poisonings	Electrocution
Alborz	0.44	-0.01	0.06	0.06	0.01	0.05	0.03	0.09	0.08	0.02
Ardebil	0.73	0	0.07	0.01	0.04	0.12	0.09	0.2	0.14	0.03
Bushehr	0.61	0.02	0.07	0	0.04	0.06	0.07	0.25	0.02	0.03
Chahar Mahaal and Bakhtiari	1.44	0.04	0.13	0.01	0.13	0.14	0.08	0.35	0.07	0.04
East Azarbayejan	1.04	0.04	0.09	0.01	0.1	0.11	0.08	0.14	0.12	0.03
Fars	1.15	0.03	0.11	0.04	0.09	0.07	0.09	0.21	0.07	0.03
Gilan	1.01	0.03	0.06	0.01	0.01	0.08	0.06	0.25	0.04	0.02
Golestan	0.78	-0.01	0.07	0	0.03	0.13	0.06	0.18	0.06	0.02
Hamadan	1.24	0.05	0.13	0.02	0.06	0.16	0.09	0.16	0.11	0.03
Hormozgan	0.91	0.01	0.08	0.01	0.08	0.15	0.07	0.32	0.07	0.04
Ilam	1.08	0.06	0.09	0.01	0.07	0.15	0.1	0.3	0.04	0.02
Iran (Islamic Republic of)	0.88	0.02	0.09	0.02	0.07	0.14	0.07	0.19	0.08	0.03
Isfahan	1.01	0	0.09	0.02	0.04	0.15	0.06	0.16	0.06	0.02
Kerman	0.86	0.03	0.07	0.03	0.11	0.14	0.1	0.15	0.07	0.04
Kermanshah	0.96	0.1	0.12	0.04	0.1	0.11	0.11	0.16	0.08	0.03
Khorasan-e-Razavi	0.84	0.04	0.13	0.03	0.13	0.16	0.11	0.13	0.11	0.04
Khuzestan	0.58	-0.01	0.05	0	0.05	0.16	0.05	0.33	0.04	0.03
Kohgiluyeh and Boyer-Ahmad	0.98	0.03	0.12	0	0.13	0.21	0.08	0.34	0.11	0.04
Kurdistan	1	0.05	0.12	0.02	0.09	0.14	0.11	0.26	0.07	0.04
Lorestan	1.17	0.05	0.08	0	0.05	0.19	0.07	0.27	0.1	0.03
Markazi	0.75	0.03	0.11	0.01	0.07	0.13	0.09	0.19	0.07	0.02
Mazandaran	1.05	0.01	0.08	0.01	0.04	0.08	0.04	0.23	0.06	0.02
North Khorasan	0.81	0.01	0.1	0.01	0.04	0.15	0.09	0.14	0.14	0.03
Qazvin	0.63	-0.01	0.09	0	0.04	0.08	0.07	0.22	0.11	0.04
Qom	0.34	-0.01	0.07	0.01	0.03	0.29	0.05	0.11	0.05	0.03
Semnan	0.51	0.01	0.07	0	0.06	0.07	0.06	0.13	0.07	0.03
Sistan and Baluchistan	2.01	0.04	0.13	0.09	0.22	0.34	0.16	0.33	0.06	0.05
South Khorasan	0.57	0	0.09	0	0.1	0.13	0.08	0.1	0.11	0.03
Tehran	0.38	0.01	0.06	0.06	0.04	0.17	0.03	0.05	0.06	0.01
West Azarbayejan	0.73	0.05	0.08	0.03	0.1	0.14	0.08	0.21	0.06	0.03
Yazd	0.89	0.02	0.1	0	0.07	0.14	0.08	0.15	0.07	0.03
Zanjan	1.42	0.04	0.18	0.01	0.08	0.2	0.09	0.28	0.16	0.05

Table S30. Contribution of injuries to change in life expectancy from 1990 to 2023 in females

Province	Transport injuries	Self harm	Falls	Fire, heat, and hot substances	Interpersonal violence	Exposure to mechanical forces	Adverse effects of medical treatment	Poisonings	Drowning	Electrocution
Alborz	0.19	0.01	0.03	0	0.03	0.02	0.03	0.04	0.07	0
Ardebil	0.63	0.06	0.05	0.03	0.02	0.07	0.07	0.13	0.17	0.02
Bushehr	0.36	0.05	0.04	0.02	0	0.03	0.04	0.01	0.17	0.01
Chahar Mahaal and Bakhtiari	1.28	0.09	0.09	0.13	0.01	0.1	0.05	0.06	0.29	0.04
East Azarbayejan	0.89	0.08	0.07	0.08	0.01	0.07	0.06	0.12	0.12	0.02
Fars	0.65	0.09	0.06	0.07	0.01	0.03	0.05	0.05	0.16	0.02
Gilan	0.44	0.05	0.03	0.01	0.01	0.04	0.04	0.03	0.2	0.01
Golestan	0.41	0.04	0.03	0.01	0	0.06	0.04	0.04	0.09	0.01
Hamadan	0.67	0.06	0.07	0.05	0.02	0.07	0.05	0.07	0.11	0.02
Hormozgan	0.45	0.03	0.04	0.06	0	0.05	0.05	0.04	0.25	0.02
Ilam	0.98	0.16	0.06	0.06	0.02	0.08	0.07	0.04	0.21	0.02
Iran (Islamic Republic of)	0.57	0.06	0.06	0.06	0.01	0.07	0.05	0.06	0.13	0.01
Isfahan	0.45	0.02	0.05	0.02	0.01	0.07	0.04	0.04	0.08	0.01
Kerman	0.48	0.04	0.03	0.08	0	0.05	0.05	0.04	0.11	0.02
Kermanshah	0.49	0.16	0.07	0.07	0.02	0.04	0.08	0.06	0.08	0.01
Khorasan-e-Razavi	0.58	0.08	0.07	0.11	0.02	0.08	0.08	0.08	0.08	0.02
Khuzestan	0.38	0.05	0.03	0.04	0.01	0.08	0.03	0.03	0.26	0.02
Kohgiluyeh and Boyer-Ahmad	0.87	0.11	0.09	0.14	0.01	0.14	0.06	0.1	0.28	0.04
Kurdistan	0.9	0.1	0.09	0.08	0.02	0.09	0.08	0.06	0.2	0.03
Lorestan	0.89	0.13	0.06	0.03	0.01	0.09	0.06	0.09	0.16	0.02
Markazi	0.5	0.03	0.08	0.05	0.01	0.07	0.07	0.05	0.17	0
Mazandaran	0.47	0.03	0.04	0.02	0	0.03	0.03	0.04	0.1	0
North Khorasan	0.5	0.04	0.05	0.02	0.01	0.06	0.06	0.09	0.07	0.01
Qazvin	0.43	0.02	0.07	0.02	0.01	0.05	0.06	0.1	0.17	0.02
Qom	0.15	0.01	0.03	0.01	0.01	0.09	0.04	0.02	0.06	0.01
Semnan	0.31	0.02	0.04	0.04	0.01	0.04	0.05	0.05	0.1	0.01
Sistan and Baluchistan	1.65	0.14	0.08	0.2	0.05	0.17	0.09	0.05	0.23	0.05
South Khorasan	0.41	0.02	0.04	0.06	0	0.05	0.06	0.08	0.06	0.01
Tehran	0.21	0.01	0.06	0.02	0.02	0.07	0.03	0.03	0.02	0
West Azarbayejan	0.58	0.1	0.07	0.08	0.02	0.1	0.06	0.06	0.24	0.02
Yazd	0.47	0.03	0.05	0.04	0	0.05	0.05	0.04	0.12	0.01
Zanjan	1.65	0.08	0.18	0.07	0.02	0.18	0.05	0.19	0.28	0.04

Table S31. Contribution of injuries to change in life expectancy from 1990 to 2023 in males

Province	Transport injuries	Self harm	Falls	Interpersonal violence	Exposure to mechanical forces	Fire, heat, and hot substances	Drowning	Adverse effects of medical treatment	Electrocution	Poisonings
Alborz	0.65	-0.03	0.08	0.08	0.08	0.02	0.11	0.04	0.03	0.11
Ardebil	0.77	-0.06	0.09	0	0.16	0.05	0.23	0.11	0.04	0.14
Bushehr	0.84	-0.01	0.1	0	0.09	0.05	0.33	0.09	0.04	0.03
Chahar Mahaal and Bakhtiari	1.66	-0.02	0.16	0	0.16	0.12	0.4	0.1	0.04	0.07
East Azarbayejan	1.16	0.01	0.09	0.01	0.14	0.11	0.15	0.09	0.04	0.11
Fars	1.58	-0.02	0.16	0.06	0.11	0.11	0.26	0.12	0.04	0.08
Gilan	1.5	0.01	0.09	0.01	0.13	0.02	0.3	0.07	0.03	0.04
Golestan	1.16	-0.06	0.1	-0.01	0.18	0.04	0.27	0.07	0.03	0.07
Hamadan	1.69	0.03	0.18	0.03	0.23	0.08	0.21	0.11	0.04	0.14
Hormozgan	1.24	-0.01	0.11	0.02	0.22	0.1	0.39	0.09	0.07	0.09
Ilam	1.2	-0.03	0.1	0	0.19	0.08	0.38	0.13	0.03	0.04
Iran (Islamic Republic of)	1.17	-0.01	0.11	0.03	0.2	0.09	0.23	0.09	0.04	0.1
Isfahan	1.45	-0.02	0.12	0.02	0.22	0.06	0.22	0.07	0.03	0.08
Kerman	1.17	0.01	0.1	0.06	0.2	0.14	0.17	0.13	0.07	0.08
Kermanshah	1.3	0.05	0.15	0.05	0.17	0.13	0.23	0.13	0.05	0.1
Khorasan-e-Razavi	1.03	0.01	0.17	0.04	0.24	0.15	0.17	0.13	0.06	0.14
Khuzestan	0.77	-0.06	0.07	-0.01	0.23	0.07	0.4	0.06	0.04	0.05
Kohgiluyeh and Boyer-Ahmad	1.04	-0.04	0.15	0	0.28	0.11	0.39	0.09	0.04	0.11
Kurdistan	1.07	-0.01	0.14	0.02	0.18	0.1	0.31	0.14	0.06	0.07
Lorestan	1.37	-0.03	0.09	0	0.26	0.06	0.36	0.07	0.05	0.1
Markazi	0.95	0.02	0.14	0.02	0.2	0.09	0.21	0.11	0.05	0.09
Mazandaran	1.61	-0.02	0.12	0.02	0.13	0.05	0.35	0.05	0.03	0.08
North Khorasan	1.03	-0.02	0.15	0.02	0.23	0.06	0.2	0.12	0.05	0.19
Qazvin	0.8	-0.03	0.11	0	0.11	0.05	0.26	0.08	0.06	0.12
Qom	0.54	-0.02	0.09	0.01	0.45	0.05	0.14	0.05	0.04	0.07
Semnan	0.7	0	0.09	0	0.1	0.07	0.16	0.08	0.05	0.09
Sistan and Baluchistan	2.33	-0.05	0.16	0.12	0.48	0.23	0.42	0.21	0.04	0.06
South Khorasan	0.76	-0.01	0.13	0	0.21	0.13	0.15	0.1	0.05	0.15
Tehran	0.52	0	0.05	0.08	0.26	0.06	0.07	0.03	0.02	0.09
West Azarbayejan	0.84	0	0.09	0.03	0.18	0.11	0.19	0.1	0.04	0.06
Yazd	1.23	0.01	0.14	0.01	0.21	0.09	0.18	0.11	0.05	0.09
Zanjan	1.12	0	0.19	0	0.21	0.09	0.28	0.12	0.06	0.12
