# Supplementary material for: Burden of 292 causes of death and life expectancy decomposition in Iran, 1990–2023: a systematic analysis for the Global Burden of Disease Study 2023
Source: Lancet Glob Health. 2026 Apr 23;14(5):e734–48. doi: 10.1016/S2214-109X(26)00031-8 (PMC13122295; doi:10.1016/S2214-109X(26)00031-8)
Supplement: Supplementary appendix 3 [file mmc3.pdf]

THE LANCET
Global Health

Supplementary appendix 3

This appendix formed part of the original submission and has been peer reviewed. We post it as supplied by the authors.

Supplement to: GBD 2023 Iran Collaborators. Burden of 292 causes of death and life expectancy decomposition in Iran, 1990–2023: a systematic analysis for the Global Burden of Disease Study 2023. *Lancet Glob Health* 2026; **14:** e734–48.

Appendix 3: Authorship appendix to "Burden of 292 causes of death and life expectancy decomposition in Iran, 1990-2023: a systematic analysis for the Global Burden of Disease Study 2023"

This appendix provides further authorship detail for "Burden of 292 causes of death and life expectancy decomposition in Iran, 1990-2023: a systematic analysis for the Global Burden of Disease Study 2023"

Table of Contents

Appendix 3: Authorship appendix to "Burden of 292 causes of death and life expectancy decomposition in Iran, 1990-2023: a systematic analysis for the Global Burden of Disease Study 2023" 1

GBD 2023 Iran Collaborators 2

Affiliations 2

Authors' Contributions... 6

Providing data or critical feedback on data sources 6

Developing methods or computational machinery 6

Providing critical feedback on methods or results 6

Drafting the work or revising it critically for important intellectual content 7

Managing the estimation or publications process.. 7

GBD 2023 Iran Collaborators

Sadaf G Sepanlou, Hossein Rezaei Aliabadi, Madineh Abbasi, Hedayat Abbastabar, Arash Abdollahi, Hassan Abolhassani, Meysam Abolmaali, Dariush Abtahi, Seyed Mohammad Kazem Aghamir, Ali Ahmadi, Marjan Ajami, Mehran Alijanzadeh, Masoud Aman Mohammadi, Sohrab Amiri, Mohammad Hosein Amirzade-Iranaq, Saeid Anvari, Jalal Arabloo, Saeed Asgary, Seyyed Shamsadin Athari, Haleh Ayatollahi, Najmeh Bahmanziari, Mohammad-Mahdi Bastan, Maryam Bemanalizadeh, Milad Bonakdar Hashemi, Omid Dadras, Nicole Davis Weaver, Azizallah Dehghan, Shirin Djalalinia, Saeid Doaei, Ebrahim Eini, Sharareh Eskandarieh, Rana Ezzeddini, Aliasghar Fakhri-Demeshghieh, Majid Fasihi Harandi, Alireza Feizkhah, Behzad Foroutan, Fataneh Ghadirian, Sadegh Ghafarian, Fatemeh Ghaffarifar, Abolfazl Ghahramani, Sulmaz Ghahramani, Mahsa Ghajarzadeh, Amirhossein Ghaseminejad-Raeini, Ahmad Ghashghaee, Mahsa Ghorbani, Pouya Goleij, Mahdi Gouravani, Farrokh Habibzadeh, Zahra Hadian, Dariush Haghmorad, Nasrin Hanifi, Hamidreza Hasani, Ali Hasanpour- Dehkordi, Mahgol Sadat Hassan Zadeh Tabatabaei, Shokoufeh Hassani, Mohammad Heidari, Mojtaba Heydari, Mohammad-Salar Hosseini, Kiavash Hushmandi, Jalil Jaafari, Morteza Jafarinia, Kasra Jahankhani, Sepide Javankiani, Ali Kabir, Leila R Kalankesh, Samad Karkhah, Hengameh Kasraei, Sina Kazemian, Faham Khamesipour, Mohammad Khammarnia, Maryam Khayamzadeh, Sepehr Khosravi, Ali-Asghar Kolahi, Farzad Kompani, Razzagh Mahmoudi, Reza Malekzadeh, Vahid Mansouri, Hamid Reza Marateb, Hossein Masoumi-Asl, Elahe Meftah, Mojgan Mirghafourvand, Noushin Mohammadifard, Ibrahim Mohammadzadeh, Mohammad Mohseni, Ali H Mokdad, AmirAli Moodi Ghalibaf, Yousef Moradi, Mohammad Moradi-Joo, Amin Mousavi Khaneghah, Ayoub Nafei, Noureddin Nakhostin Ansari, Ali Nasrollahizadeh, Amir Nasrollahizadeh, Amir Nasrollahizadeh, Athare Nazri-Panjaki, Jalil Nejati, Ali Nikoobar, Hassan Okati-Aliabad, Reza Pourbabaki, Reza Rabiei, Hadi Raeisi Shahraki, Afarin Rahimi-Movaghar, Vafa Rahimi-Movaghar, Shayan Rahmani, Vahid Rahmanian, Ashkan Rasouli-Saravani, Ramin Ravangard, Nazila Rezaei, Negar Rezaei, Nima Rezaei, Peyman Rezaei Hachesu, Mohsen Rezaeian, Gholamreza Roshandel, Morteza Rostamian, Masoumeh Sadeghi, Maryam Saeedi, Sahar Saeedi Moghaddam, Mehdi Safari, Alireza Saghafi, Amirhossein Sahebkar, Leili Salehi, Amir Salek Farrokhi, Sohrab Salimi, Hossein Samadi Kafil, Mahan Shafie, Hamid R Shahsavari, Alireza Shakeri, Ali Shamekh, Mehran Shams-Beyranvand, Amin Sharifan, Amir Shiani, Reza Shirkoohi, Sina Shool, Seyed Afshin Shorofi, Soroush Soraneh, Seyed-Amir Tabatabaeizadeh, Ramin Tabibi, Alireza Tahamtan, Moslem Taheri Soodejani, Razieh Tavakoli Oliaee, Seyed Mohammad Tavangar, Omid Vakili, Sajad Yaghoubi, Habib Yaribeygi, Hamed Zandian, Alireza Zangeneh, Kourosh Zarea, and Mohsen Naghavi.

Affiliations

Digestive Diseases Research Institute (DDRI) (S G Sepanlou MD, Prof R Malekzadeh MD, V Mansouri MD), Advanced Diagnostic and Interventional Radiology Research Center (ADIR) (H Abbastabar PhD), Research Center for Immunodeficiencies (H Abolhassani PhD, Prof N Rezaei PhD), Urology Research Center (Prof S Aghamir PhD), Universal Scientific Education and Research Network (USERN) (M Amirzade-Iranaq DDS), School of Public Health (N Bahmanziari PhD), Non-communicable Diseases Research Center (M Bastan MD, S Rahmani MD, N Rezaei MD, N Rezaei PhD), Department of Pediatric Neurology (M Bemanalizadeh MD), Multiple Sclerosis Research Center (S Eskandarieh PhD), Department of Ophthalmology (S Ghafarian MD), School of Medicine (A Ghaseminejad-Raeini MD, M Gouravani MD), Sina Trauma and Surgery Research Center (M Hassan Zadeh Tabatabaei MD, Prof V Rahimi-Movaghar MD, S Shool MD), The Institute of Pharmaceutical Sciences (TIPS) (S Hassani PhD), Department of General Surgery (S Javankiani MD), Cardiac Primary Prevention Research Center (S Kazemian MD),

Department of Cardiac Electrophysiology (S Kazemian MD), Children's Medical Center (Prof F Kompani MD), Department of Physiotherapy (Prof N Nakhostin Ansari PhD), Research Center for War-affected People (Prof N Nakhostin Ansari PhD), Tehran Heart Center (A Nasrollahizadeh MD, A Nasrollahizadeh MD), Institute for Health Metrics and Evaluation (A Nasrollahizadeh MD, A Nasrollahizadeh MD), Iranian National Center for Addiction Studies (Prof A Rahimi-Movaghar MD), Endocrinology and Metabolism Research Institute (N Rezaei PhD), Endocrinology and Metabolism Population Sciences Institute (S Saeedi Moghaddam MSc), Non-communicable Diseases Research Center (NCDRC) (A Saghafi MD), Department of Neurology (M Shafie MD), Cancer Research Center (R Shirkoohi PhD), Cancer Biology Research Center (R Shirkoohi PhD), Department of Pathology (Prof S Tavangar MD), Tehran University of Medical Sciences, Tehran, Iran; Department of Biostatistics (H Rezaei Aliabadi PhD), School of Medicine (H Rezaei Aliabadi PhD), Health Policy Research Center (S Ghahramani MD, H Kasraei MD), Poostchi Ophthalmology Research Center (M Heydari PhD), Shiraz Neuroscience Research Center (M Jafarinia PhD), Non-communicable Disease Research Center (Prof R Malekzadeh MD), Clinical Research Development Center (E Meftah MD), Professor Alborzi Clinical Microbiology Research Center (E Meftah MD), Department of Health Services Management (Prof R Ravangard PhD), Basic Sciences in Infectious Diseases Research Center (R Tavakoli Oliaee PhD), Shiraz University of Medical Sciences, Shiraz, Iran; Infectious and Tropical Research Center (M Abbasi PhD), Research Center for Evidence-Based Medicine (M Hosseini MD), School of Management and Medical Informatics (L R Kalankesh PhD), Faculty of Nursing and Midwifery (Prof M Mirghafourvand PhD), Department of Health Information Technology (P Rezaei Hachesu PhD), Drug Applied Research Center (H Samadi Kafil PhD), Faculty of Medicine (A Shamekh MD), Aging Research Institute (A Shamekh MD), Tabriz University of Medical Sciences, Tabriz, Iran; Minimally Invasive Surgery Research Center (A Abdollahi MD, A Kabir MD), Department of Neurosurgery (M Abolmaali MD), Health Management and Economics Research Center (J Arabloo PhD, H Ayatollahi PhD, A Nasrollahizadeh MD), Department of Health Information Management (H Ayatollahi PhD), School of Medicine (M Bastan MD), Eye Research Center (H Kasraei MD), Department of Pediatrics (H Masoumi-Asl MD), Center for Technology and Innovation in Cardiovascular Informatics (S Shool MD), Iran University of Medical Sciences, Tehran, Iran; Department of Medical Biochemistry and Biophysics (H Abolhassani PhD), Karolinska Institute, Stockholm, Sweden; Khatam Al-anbia Hospital (M Abolmaali MD), Shefa Neuroscience Research Center, Tehran, Iran; Department of Anesthesiology (Prof D Abtahi MD, S Salimi MD, A Shakeri MD), Department of Epidemiology (Prof A Ahmadi PhD), National Nutrition and Food Technology Research Institute (Prof M Ajami PhD, Z Hadian PhD), Research Institute of Dental Sciences (Prof S Asgary MSc), Department of Urology (M Bonakdar Hashemi MD), Department of Community Nutrition (S Doaei PhD), School of Nursing and Midwifery (F Ghadirian PhD), Department of Immunology (K Jahankhani MSc, A Rasouli-Saravani PhD), Social Determinants of Health Research Center (Prof A Kolahi MD, A Nikoobar MD), Skull Base Research Center (I Mohammadzadeh MD), Department of Health Information Technology and Management (R Rabiei PhD), School of Medicine (S Rahmani MD), Research Institute for Health Sciences and Environment (M Safari PhD), Department of Health in Disaster & Emergency (M Safari PhD), Shahid Beheshti University of Medical Sciences, Tehran, Iran (M Khayamzadeh MD); Department of Epidemiology and Biostatistics (Prof A Ahmadi PhD, H Raeisi Shahraki PhD), Community-Oriented Nursing Midwifery Research Center (M Heidari PhD), Shahrekord University of Medical Sciences, Shahrekord, Iran; Social Determinants of Health Research Center (M Alijanzadeh PhD), School of Public Health (A Ghashghaee BSc), Department of Food Hygiene and Safety (Prof R Mahmoudi PhD), Qazvin University of Medical Sciences, Qazvin, Iran; Food and Beverages Safety Research Center (M Aman Mohammadi PhD), Department of Occupational Safety and Health (A

Ghahramani PhD), School of Medicine (S Soraneh MD), Urmia University of Medical Sciences, Urmia, Iran; Spiritual Health Research Centre (S Amiri PhD), Nephrology and Urology Research Center (K Hushmandi PhD), Baqiyatallah University of Medical Sciences, Tehran, Iran; Regenerative Medicine, Organ Procurement and Transplantation Multi-disciplinary Center (S Anvari MD), School of Health (S Doaei PhD), Department of Social Medicine and Epidemiology (A Feizkhah MD), Department of Environmental Health Engineering (J Jaafari PhD), Department of Medical-Surgical Nursing (S Karkhah MSc), Guilan University of Medical Sciences, Rasht, Iran; National Agency for Strategic Research in Medical Education (NASRME) (Prof S Asgary MSc), Development of Research and Technology Center (S Djalalinia PhD), Ministry of Health and Medical Education, Tehran, Iran; Department of Immunology (S Athari PhD), Department of Critical Care and Emergency Nursing (N Hanifi PhD), Zanjan University of Medical Sciences, Zanjan, Iran; Department of Pediatrics (M Bemanalizadeh MD), Isfahan Cardiovascular Research Center (N Mohammadifard PhD), Department of Health Services Management (M Mohseni PhD), Faculty of Medicine (A Nasrollahizadeh MD), Cardiac Rehabilitation Research Center (Prof M Sadeghi MD), Department of Clinical Biochemistry (O Vakili PhD), Isfahan University of Medical Sciences, Isfahan, Iran; Department of Health (O Dadras PhD), Northern Territory Government, Darwin, SA, Australia; Institute for Health Metrics and Evaluation (N Davis Weaver MPH, Prof A H Mokdad PhD, Prof M Naghavi PhD), Department of Health Metrics Sciences, School of Medicine (Prof A H Mokdad PhD, Prof M Naghavi PhD), University of Washington, Seattle, WA, USA; Department of Epidemiology and Community Medicine (A Dehghan PhD), Fasa University of Medical Sciences, Fasa, Iran; Independent Consultant, Ahvaz, Iran (E Eini MSD); Clinical Biochemistry (R Ezzeddini PhD), Department of Parasitology and Entomology (Prof F Ghaffarifar PhD), Tarbiat Modares University, Tehran, Iran; Department of Food Hygiene and Quality Control (A Fakhri-Demeshghieh PhD), University of Tehran, Tehran, Iran; Department of Medical Parasitology (Prof M Fasihi Harandi PhD), Department of Occupational Health and Safety Engineering (R Pourbabaki PhD), Kerman University of Medical Sciences, Kerman, Iran; Department of Pharmacology (Prof B Foroutan PhD), Iranshahr University of Medical Sciences, Iranshahr, Iran; Department of Radiology (M Ghajarzadeh PhD), Washington University in St. Louis, St. Louis, MO, USA; Cardiovascular Epidemiology Research Center (A Ghaseminejad-Raeini MD), Rajaie Cardiovascular Institute, Tehran, Iran; Orthodontics Department (M Ghorbani DDS), Biotechnology Research Center (Prof A Sahebkar PhD), Mashhad University of Medical Sciences, Mashhad, Iran (A Moodi Ghalibaf MD); Department of Genetics (P Goleij MSc), Sana Institute of Higher Education, Sari, Iran; Universal Scientific Education and Research Network (USERN) (P Goleij MSc), Department of Speech Therapy (A Shiani PhD), Social Development and Health Promotion Research Center (A Zangeneh MSc), Kermanshah University of Medical Sciences, Kermanshah, Iran; Global Virus Network, Middle East Region, Shiraz, Iran (F Habibzadeh MD); Department of Immunology (D Haghmorad PhD), Cancer Research Center (D Haghmorad PhD), Research Center of Physiology (H Yaribeygi PhD), Semnan University of Medical Sciences, Semnan, Iran; Department of Ophthalmology (H Hasani MD), Research Center for Health, Safety and Environment (Prof L Salehi PhD), School of Medicine (M Shams-Beyranvand MSc), Alborz University of Medical Sciences, Karaj, Iran; Department of Medical Surgical (Prof A Hasanpour- Dehkordi PhD), Shahroud University of Medical Sciences, Shahrekord, Iran; Food and Drug Research Center (F Khamesipour PhD), Iran Food and Drug Administration, Tehran, Iran; Health Promotion Research Center (M Khammarnia PhD, J Nejati PhD, H Okati-Aliabad PhD), Department of Health Promotion (A Nazri-Panjaki MSc), Zahedan University of Medical Sciences, Zahedan, Iran; Academy of Medical Science, Tehran, Iran (M Khayamzadeh MD); Manchester Centre for Clinical Neurosciences (S Khosravi MD), Northern Care Alliance NHS Foundation Trust, Salford, United Kingdom;

Department of Clinical Research (S Khosravi MD), Icahn School of Medicine at Mount Sinai, New York City, NY, USA; Department of Biomedical Engineering (H Marateb PhD), University of Isfahan, Isfahan, Iran; Institute for Research and Innovation in Health (IRIS) (H Marateb PhD), Universitat Politècnica de Catalunya (Barcelona Tech - UPC) (Polytechnic University of Catalonia), Barcelona, Spain; Department of Health Services Management (M Mohseni PhD), Iran University of Medical Sciences, Iran, Iran; Department of Epidemiology and Biostatistics (Y Moradi PhD), Kurdistan University of Medical Sciences, Sanandaj, Iran; Social Determinants of Health Research Center (M Moradi-Joo PhD), Yasuj University of Medical Sciences, Yasuj, Iran; Faculty of Biotechnologies (BioTech) (Prof A Mousavi Khaneghah PhD), ITMO University, Saint Petersburg, Russia; Elderly Health Research Center (A Nafei PhD), Research and Academic Institution, Tehran, Iran; Department of Public Health (V Rahmanian PhD), Torbat Jam Faculty of Medical Sciences, Torbat Jam, Iran; Network of Immunity in Infection, Malignancy and Autoimmunity (NIIMA) (Prof N Rezaei PhD), Universal Scientific Education and Research Network (USERN), Tehran, Iran; Department of Epidemiology and Biostatistics (Prof M Rezaeian PhD), Rafsanjan University of Medical Sciences, Rafsanjan, Iran; Golestan Research Center of Gastroenterology and Hepatology (G Roshandel PhD), Department of Microbiology (A Tahamtan PhD), Golestan University of Medical Sciences, Gorgan, Iran; School of Medicine (M Rostamian PhD), Gonabad University of Medical Sciences, Gonabad, Iran; Department of Nursing and Midwifery (M Saeedi PhD), Saveh University of Medical Sciences, Saveh, Iran; Global Health Economy Research Team (S Saeedi Moghaddam MSc), Kiel Institute for the World Economy, Kiel, Germany; Clinical Research Development Center (CRDC) (A Saghafi MD), Qom University of Medical Sciences, Qom, Iran; Center for Global Health Research (Prof A Sahebkar PhD), Saveetha University, Chennai, India; Department of Health Education & Promotion (Prof L Salehi PhD), A.C.S. Medical College and Hospital, Karaj, Iran; Department of Immunology (A Salek Farrokhi PhD), Pasteur Institute of Iran, Tehran, Iran; Department of Chemistry (H R Shahsavari PhD), Institute for Advanced Studies in Basic Sciences (IASBS), Zanjan, Iran; Department for Evidence-based Medicine and Evaluation (A Sharifan PharmD), University for Continuing Education Krems, Krems, Austria; Department of Medical-Surgical Nursing (S Shorofi PhD), Mazandaran University of Medical Sciences, Sari, Iran; Department of Nursing and Health Sciences (S Shorofi PhD), Flinders University, Adelaide, SA, Australia; School of Medicine (S Soraneh MD), Babol University of Medical Sciences, Babol, Iran; Department of Basic Medical Sciences (S Tabatabaeizadeh PhD), Department of Internal Medicine (S Tabatabaeizadeh PhD), Islamic Azad University, Mashhad, Iran; Department of Health, Safety, and Environmental Management (R Tabibi PhD), Abadan School of Medical Sciences, Abadan, Iran; Department of Biostatistics and Epidemiology (M Taheri Soodejani PhD), Shahid Sadoughi University of Medical Sciences, Yazd, Iran; Department of Basic Medical Sciences (S Yaghoubi PhD), Neyshabur University of Medical Sciences, Neyshabur, Iran; Centre for Public Health and Wellbeing (H Zandian PhD), University of the West of England, Bristol, United Kingdom; Nursing Care Research Center in Chronic Diseases (Prof K Zarea PhD), Ahvaz Jundishapur University of Medical Sciences, Ahvaz, Iran.

Authors' Contributions

Providing data or critical feedback on data sources

Madineh Abbasi, Hassan Abolhassani, Ali Ahmadi, Sohrab Amiri, Saeid Anvari, Jalal Arabloo, Seyyed Shamsadin Athari, Mohammad-Mahdi Bastan, Milad Bonakdar Hashemi, Ebrahim Eini, Sharareh Eskandarieh, Aliasghar Fakhri-Demeshghieh, Majid Fasihi Harandi, Alireza Feizkhah, Mahsa Ghajarzadeh, Ahmad Ghashghaee, Pouya Goleij, Dariush Haghmorad, Nasrin Hanifi, Ali Hasanpour- Dehkordi, Mahgol Sadat Hassan Zadeh Tabatabaei, Shokoufeh Hassani, Mohammad Heidari, Jalil Jaafari, Morteza Jafarinia, Sepide Javankiani, Leila R Kalankesh, Samad Karkhah, Faham Khamesipour, Mohammad Khammarnia, Razzagh Mahmoudi, Hossein Masoumi-Asl, Ali H Mokdad, Yousef Moradi, Mohammad Moradi-Joo, Mohsen Naghavi, Amir Nasrollahizadeh, Amir Nasrollahizadeh, Reza Pourbabaki, Hadi Raeisi Shahraki, Afarin Rahimi-Movaghar, Vafa Rahimi-Movaghar, Nima Rezaei, Hossein Rezaei Aliabadi, Maryam Saeedi, Mehdi Safari, Leili Salehi, Hossein Samadi Kafil, Mahan Shafie, Ali Shamekh, Mehran Shams-Beyranvand, Amin Sharifan, Soroush Soraneh, Alireza Tahamtan, Moslem Taheri Soodejani, Sajad Yaghoubi, Habib Yaribeygi, and Kourosh Zarea.

Developing methods or computational machinery

Ali H Mokdad and Mohsen Naghavi.

Providing critical feedback on methods or results

Madineh Abbasi, Hedayat Abbastabar, Arash Abdollahi, Hassan Abolhassani, Meysam Abolmaali, Dariush Abtahi, Seyed Mohammad Kazem Aghamir, Ali Ahmadi, Mehran Alijanzadeh, Masoud Aman Mohammadi, Sohrab Amiri, Saeid Anvari, Jalal Arabloo, Saeed Asgary, Seyyed Shamsadin Athari, Haleh Ayatollahi, Najmeh Bahmanziari, Mohammad-Mahdi Bastan, Maryam Bemanalizadeh, Milad Bonakdar Hashemi, Omid Dadras, Azizallah Dehghan, Shirin Djalalinia, Saeid Doaei, Ebrahim Eini, Sharareh Eskandarieh, Rana Ezzeddini, Aliasghar Fakhri-Demeshghieh, Alireza Feizkhah, Sadegh Ghafarian, Fatemeh Ghaffarifar, Abolfazl Ghahramani, Sulmaz Ghahramani, Amirhossein Ghaseminejad-Raeini, Ahmad Ghashghaee, Mahsa Ghorbani, Farrokh Habibzadeh, Nasrin Hanifi, Hamidreza Hasani, Ali Hasanpour- Dehkordi, Mahgol Sadat Hassan Zadeh Tabatabaei, Shokoufeh Hassani, Mohammad Heidari, Mojtaba Heydari, Mohammad-Salar Hosseini, Kiavash Hushmandi, Jalil Jaafari, Morteza Jafarinia, Sepide Javankiani, Ali Kabir, Leila R Kalankesh, Samad Karkhah, Sina Kazemian, Faham Khamesipour, Mohammad Khammarnia, Maryam Khayamzadeh, Ali-Asghar Kolahi, Farzad Kompani, Razzagh Mahmoudi, Vahid Mansouri, Hamid Reza Marateb, Ibrahim Mohammadzadeh, Mohammad Mohseni, Ali H Mokdad, AmirAli Moodi Ghalibaf, Yousef Moradi, Mohammad Moradi-Joo, Amin Mousavi Khaneghah, Ayoub Nafei, Mohsen Naghavi, Ali Nasrollahizadeh, Amir Nasrollahizadeh, Amir Nasrollahizadeh, Athare Nazri-Panjaki, Jalil Nejati, Ali Nikoobar, Hassan Okati-Aliabad, Reza Pourbabaki, Reza Rabiei, Hadi Raeisi Shahraki, Afarin Rahimi-Movaghar, Vafa Rahimi-Movaghar, Shayan Rahmani, Vahid Rahmanian, Ashkan Rasouli-Saravani, Ramin Ravangard, Nazila Rezaei, Negar Rezaei, Nima Rezaei, Peyman Rezaei Hachesu, Mohsen Rezaeian, Gholamreza Roshandel, Maryam Saeedi, Sahar Saeedi Moghaddam, Mehdi Safari, Alireza Saghafi, Leili Salehi, Amir Salek Farrokhi, Sohrab Salimi, Hossein Samadi Kafil, Sadaf G Sepanlou, Mahan Shafie, Hamid R Shahsavari, Alireza Shakeri, Ali Shamekh, Mehran Shams-Beyranvand, Amin Sharifan, Amir Shiani, Sina Shool, Seyed Afshin Shorofi, Soroush Soraneh, Seyed-Amir Tabatabaeizadeh, Ramin Tabibi, Alireza Tahamtan, Razieh Tavakoli Oliaee, Seyed Mohammad Tavangar, Omid Vakili, Hamed Zandian, and Kourosh Zarea.

Drafting the work or revising it critically for important intellectual content

Madineh Abbasi, Hedayat Abbastabar, Arash Abdollahi, Hassan Abolhassani, Meysam Abolmaali, Ali Ahmadi, Marjan Ajami, Mohammad Hosein Amirzade-Iranaq, Saeid Anvari, Jalal Arabloo, Saeed Asgary, Seyyed Shamsadin Athari, Mohammad-Mahdi Bastan, Maryam Bemanalizadeh, Milad Bonakdar Hashemi, Ebrahim Eini, Sharareh Eskandarieh, Rana Ezzeddini, Aliasghar Fakhri-Demeshghieh, Behzad Foroutan, Fataneh Ghadirian, Sadegh Ghafarian, Sulmaz Ghahramani, Amirhossein Ghaseminejad-Raeini, Mahsa Ghorbani, Mahdi Gouravani, Farrokh Habibzadeh, Zahra Hadian, Nasrin Hanifi, Ali Hasanpour- Dehkordi, Mahgol Sadat Hassan Zadeh Tabatabaei, Mohammad-Salar Hosseini, Kasra Jahankhani, Sepide Javankiani, Ali Kabir, Samad Karkhah, Hengameh Kasraei, Sina Kazemian, Mohammad Khammarnia, Sepehr Khosravi, Reza Malekzadeh, Vahid Mansouri, Hamid Reza Marateb, Elahe Meftah, Mojgan Mirghafourvand, Noushin Mohammadifard, Ali H Mokdad, AmirAli Moodi Ghalibaf, Yousef Moradi, Amin Mousavi Khaneghah, Ayoub Nafei, Mohsen Naghavi, Noureddin Nakhostin Ansari, Ali Nasrollahizadeh, Amir Nasrollahizadeh, Amir Nasrollahizadeh, Jalil Nejati, Hadi Raeisi Shahraki, Afarin Rahimi-Movaghar, Vafa Rahimi-Movaghar, Shayan Rahmani, Ashkan Rasouli-Saravani, Ramin Ravangard, Nima Rezaei, Hossein Rezaei Aliabadi, Gholamreza Roshandel, Morteza Rostamian, Masoumeh Sadeghi, Maryam Saeedi, Sahar Saeedi Moghaddam, Mehdi Safari, Alireza Saghafi, Amirhossein Sahebkar, Amir Salek Farrokhi, Hossein Samadi Kafil, Sadaf G Sepanlou, Mahan Shafie, Ali Shamekh, Mehran Shams-Beyranvand, Amin Sharifan, Reza Shirkoohi, Sina Shool, Seyed Afshin Shorofi, Soroush Soraneh, Seyed-Amir Tabatabaeizadeh, Alireza Tahamtan, Razieh Tavakoli Oliaee, Seyed Mohammad Tavangar, Alireza Zangeneh, and Kourosh Zarea.

Managing the estimation or publications process

Nicole Davis Weaver, Ali H Mokdad, and Mohsen Naghavi.
